# Linker-Dependent Folding Rationalizes PROTAC Cell Permeability

**DOI:** 10.1021/acs.jmedchem.2c00877

**Published:** 2022-09-28

**Authors:** Vasanthanathan Poongavanam, Yoseph Atilaw, Stephan Siegel, Anja Giese, Lutz Lehmann, Daniel Meibom, Mate Erdelyi, Jan Kihlberg

**Affiliations:** † Department of Chemistry—BMC, Uppsala University, Box 576, 75123 Uppsala, Sweden; ‡ Drug Discovery Sciences, Bayer AG, 13342 Berlin, Germany; § Drug Discovery Sciences, Bayer AG, 42113 Wuppertal, Germany

## Abstract

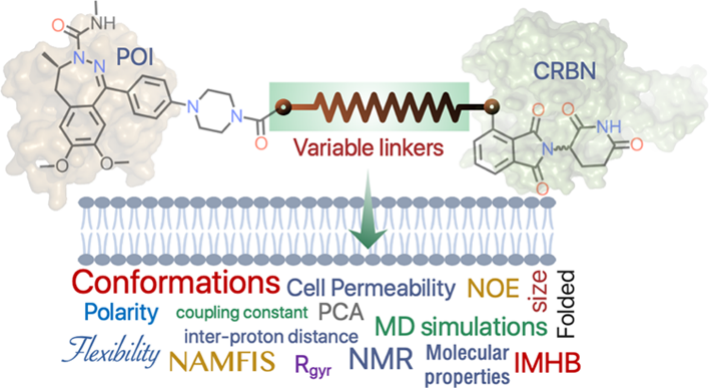

Proteolysis-targeting chimeras (PROTACs) must be cell permeable to reach their target proteins. This is challenging as the bivalent structure of PROTACs puts them in chemical space at, or beyond, the outer limits of oral druggable space. We used NMR spectroscopy and molecular dynamics (MD) simulations independently to gain insights into the origin of the differences in cell permeability displayed by three flexible cereblon PROTACs having closely related structures. Both methods revealed that the propensity of the PROTACs to adopt folded conformations with a low solvent-accessible 3D polar surface area in an apolar environment is correlated to high cell permeability. The chemical nature and the flexibility of the linker were essential for the PROTACs to populate folded conformations stabilized by intramolecular hydrogen bonds, π–π interactions, and van der Waals interactions. We conclude that MD simulations may be used for the prospective ranking of cell permeability in the design of cereblon PROTACs.

## Introduction

Proteolysis-targeting chimeras (PROTACs) are heterobifunctional compounds consisting of a ligand for a protein of interest (POI) connected via a linker to an E3 ubiquitin ligase ligand.^1^ Formation of a ternary complex, in which the PROTAC brings the POI and the E3 ligase in contact, results in ubiquitinylation and subsequent degradation of the POI by the proteasome. PROTACs are attracting significant interest due to their potential to modulate targets (POIs) considered as undruggable, for example, due to the lack of well-defined pockets or grooves that allow high-affinity binding of small-molecule ligands. To date, the majority of PROTACs are based either on a Von Hippel-Lindau (VHL) or a cereblon (CRBN) E3 ligase ligand.^2^ However, all but one of the PROTACs that have entered, or are about to enter, clinical trials contain a CRBN E3 ligase ligand.^1^

The mode of action of PROTACs requires that they permeate into target cells, and cell permeability is also necessary for them to display oral bioavailability. However, PROTACs reside in chemical space at the edge of oral druggable space,^3−5^ that is, at or beyond the outer limits of the oral beyond rule of 5 (bRo5) chemical space.^6,7^ Consequently, low cell permeability and/or other pharmacokinetic deficiencies may prevent PROTACs from reaching their targets and from being absorbed after oral administration. PROTACs based on a CRBN E3 ligase ligand populate the chemical space that has some overlap with parts of bRo5 space, while those based on VHL or other E3 ligase ligands occupy more distant space.^5^ Most likely, this explains why the majority of PROTACs in the clinic are based on CRBN.

For small-molecule drugs, cell permeability is often assessed using Caco-2 cell monolayers as the results allow estimation of the compound’s oral absorption.^8^ It has been pointed out that Caco-2 assay conditions need to be optimized for PROTACs^9,10^ and that results may be difficult to interpret since PROTACs with low permeability may still induce degradation.^11^ Conflicting conclusions have been reached regarding the use of chromatographically determined descriptors of lipophilicity and polarity for prediction of the permeability of PROTACs across Caco-2 cells.^12,13^ However, the combined use of the parallel artificial membrane permeability assay (PAMPA) and lipophilic permeability efficiency (LPE) provided insights into structure–permeability relationships, and it was also suggested that Alog *P* should be kept below 5 to increase the chances for PROTACs to be cell permeable.^14^ A study on JAK-degrading PROTACs used the drop-off in potencies of a biochemical Janus kinase assay to the cell as a permeability surrogate.^15^

Since the POI and E3 ubiquitin ligase ligands usually provide little room for modification in the development of PROTACs, the linker remains as the most interesting opportunity for optimization of degradation potency, selectivity, as well as physicochemical and pharmacokinetic properties.^16^ Linear alkyl and ethylene glycol chains are the most common types of linkers but are being complemented by more rigid linkers that may contain functionalities that modulate physicochemical properties. To date, only a handful of studies, each based on a few examples, of linker–property relationships for PROTACs have been published. Thus, macrocyclization of the linker resulted in improved selectivity in the degradation of homologous POIs,^17^ while the increased plasticity of ethylene glycol as compared to an alkyl linker enhanced ternary complex formation in another study.^18^ Cell permeability increased when switching from ethylene glycol to an alkyl linker^15^ or for amide-to-ester substitutions in the linker,^19^ that is, permeability increased with increasing lipophilicity provided that it was kept within the drug-like range. Herein, we have used NMR spectroscopy and unrestrained molecular dynamics (MD) simulations independently to provide a unique insight into the origin of the differences in cell permeability displayed by three closely related PROTACs. The PROTACs are based on thalidomide as a CRBN E3 ligase ligand and target degradation of bromodomain-containing protein 4 (BRD4) but differ in the length and structure of their linkers.

## Results and Discussion

### PROTACs

**Figure 1:**
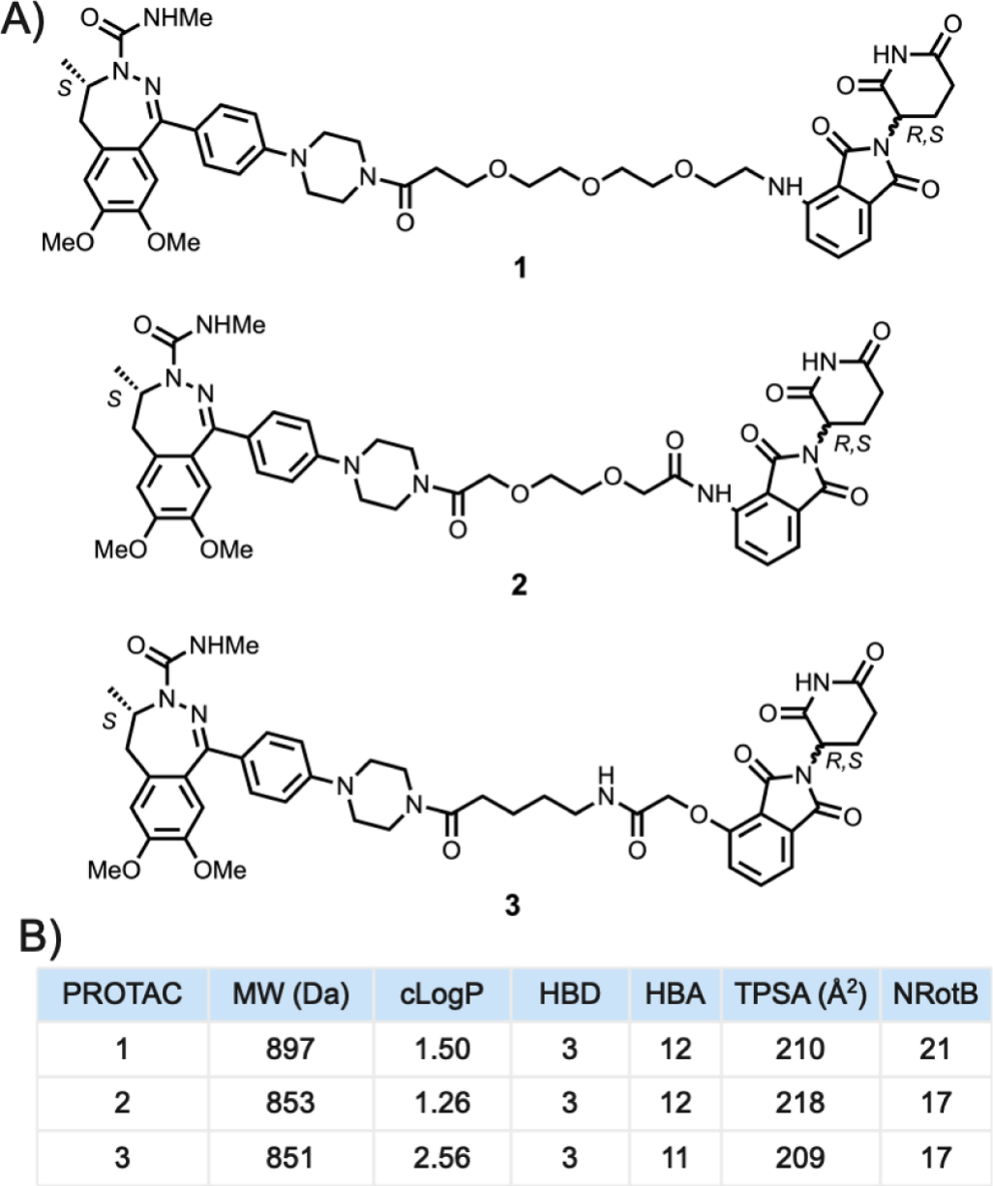
(A) Structures of PROTACs **1–3**. The three PROTACs have the same BRD4 and E3 ligase (CRBN) ligands but differ in the structures of their linkers. (B) Descriptors of Lipinski’s rule of 5^27^ and Veber’s rule^28^ were calculated for PROTACs **1–3** using MOE (version 2019.01). MW, molecular weight; cLog *P*, calculated lipophilicity; HBD, hydrogen bond donor; HBA, hydrogen bond acceptor; TPSA, topological polar surface area; and NRotB, number of rotatable bonds.

Inhibition of BRD4 is of interest for the treatment of diseases in which cell proliferation is dysregulated, for instance, cancer and viral infections.^20−22^ Small-molecule inhibitors of BRD4 can suffer from drawbacks such as inefficient reversible inhibition, BRD4 accumulation, and broad tissue distribution, which makes PROTACs that induce effective, sustained, and, in principle, tissue-specific degradation of BRD4 an interesting alternative.^23,24^ PROTACs **1–3** target the degradation of BRD4 and differ only in the structure of the linker which connects the BRD4 ligand to the thalidomide moiety (Figure 1A).^25,26^ The linker of **1** is four atoms longer than that of **2** and **3**, and **1–3** also differ in the chemical nature of the linker and in its connection to the thalidomide moiety. The three PROTACs have MW, HBA, TPSA, and NRotB outside the Ro5^27^ and Veber’s rule^28^ and consequently reside in the bRo5 chemical space (Figure 1B),^6,7^ where it may be difficult to achieve drug-like physicochemical properties and pharmacokinetics.^3−5^

PROTACs **1–3** have a low but sufficient aqueous solubility that allows them to be evaluated in in vitro assay systems (Table 1). They bind potently to the BD1 and BD2 domains of BRD4, indicating their potential for degrading this target. The ratio between the potencies for binding of a PROTAC to CRBN in a cell-based and in a biochemical assay is a surrogate for passive cell permeability,^15,29^ which may be used to select PROTACs to be progressed in drug projects. Interestingly, PROTACs **1–3** show large differences in this permeability surrogate, with permeabilities ranging from high (low ratio) via intermediate to low (high ratio) for **1**, **2,** and **3**, respectively (Table 2). As the cell/biochemical ratio for CRBN binding may be affected by intracellular binding to macromolecules and organelles, we also determined the permeabilities of **1** and **3** in the PAMPA assay, which confirmed a higher passive permeability for **1**. In addition, estimation of the passive permeability across Caco-2 cell monolayers ranked the permeabilities of the three PROTACs in the same order as the cell/biochemical ratio for binding to CRBN. There is no obvious correlation between the differences in cell permeability of **1–3** and their calculated descriptors. In fact, the PROTAC predicted to be most lipophilic (**3**) is the least permeable, while the most permeable (**1**) has a somewhat higher MW and NRotB count than **2** and **3**. We were intrigued by the consistent differences in permeability displayed by **1–3** and by the lack of obvious correlation to their structures. Therefore, we used solution-phase NMR spectroscopy and MD simulations to investigate if different conformational preferences between the PROTACs could rationalize the permeability differences.

**Table 1:** Aqueous Solubility and In Vitro Potency for PROTACs **1–3**^a^

PROTAC	solubility^b^ (mg/L)	BRD4 (BD1) IC_50_ (nM)	BRD4 (BD2) IC_50_ (nM)
**1**	56 ± 13	38 ± 4.6	188 ± 12
**2**	31 ± 12	10 ± 0.35	108 ± 4.5
**3**	63 ± 2.7	34 ± 1.6	118 ± 41

a The values for solubility are mean values ± SEM from ≥three repeats. The potencies for binding to BRD4 are mean values ± SEM originating from two or four repeats.

b Determined in PBS at pH 6.5.

**Table 2:** Permeabilities for PROTACs **1–3**^a^

PROTAC	CRBN (cell) IC_50_ (μM)	CRBN (bio) IC_50_ (μM)	CRBN, ratio cell/bio^b^	PAMPA (−log *P*_e_, cm/s)	*P*_passive_^c^ (nm/s)
**1**	0.924 ± 0.119	0.244 ± 0.028	4	6.56 ± 004	30 ± 1.5
**2**	16.9 ± 1.41	1.41 ± 0.207	12	n.d.^d^	11 ± 1.7
**3**	18.0 ± 2.07	0.667 ± 0.066	27	>7.37	6 ± 1.4

a The potencies for binding to CRBN are mean values ± SEM from five or six repeats. PAMPA data are mean values ± SEM from three repeats. Permeabilities across Caco-2 cell monolayers are mean values ± SEM from two or three repeats.

b Highly permeable PROTACs have a low cell/bio ratio, while the opposite is true for low-permeable ones.

c Passive permeability (*P*_passive_) across Caco-2 cell monolayers at pH 7.4 was calculated as the geometric mean of *P*_app_ AB and *P*_app_ BA (Table S1), i.e. *P*_passive_ = (*P*_app_AB × *P*_app_BA)^0.5^.

d Not determined.

### Determination of Conformational Ensembles

The solution conformational ensembles of the PROTACs were determined using the NMR analysis of molecular flexibility in solution (NAMFIS) algorithm, which deconvolutes time-averaged NMR data into individual conformations.^30^ NAMFIS has been used successfully to determine the solution ensembles of flexible, linear compounds including a PROTAC, as well as more rigid macrocycles.^31−35^ The selection of conformers is driven by experimental data, that is, by proton–proton distances obtained from highly accurate NOE buildup measurements. Accurate interatomic distances cannot be reliably determined from a single NOESY spectrum with an arbitrary mixing time. Use of ROESY buildups is cumbersome as these need correction for offset effects and may suffer from difficult-to-observe single-intensity alterations due to TOCSY-type artifacts for strongly coupled proton pairs. We therefore acquired NOESY buildups with seven mixing times between 100 and 700 ms and used only the strictly linear part of the initial buildups, excluding distorted peak intensities due to interference with other relaxation mechanisms or noise for weak NOEs. Theoretical conformational ensembles that cover the conformational space populated by the investigated compounds are also required as inputs for NAMFIS.^31^ These were generated by unrestrained Monte Carlo conformational searches using different force fields and implicit solvent models. The NAMFIS algorithm then identifies the conformations from the theoretical ensemble and their population that provide the best fit of the back-calculated distances to the experimentally determined values. Finally, the resulting conformational ensembles are validated as described previously.^31^

**Figure 2:**
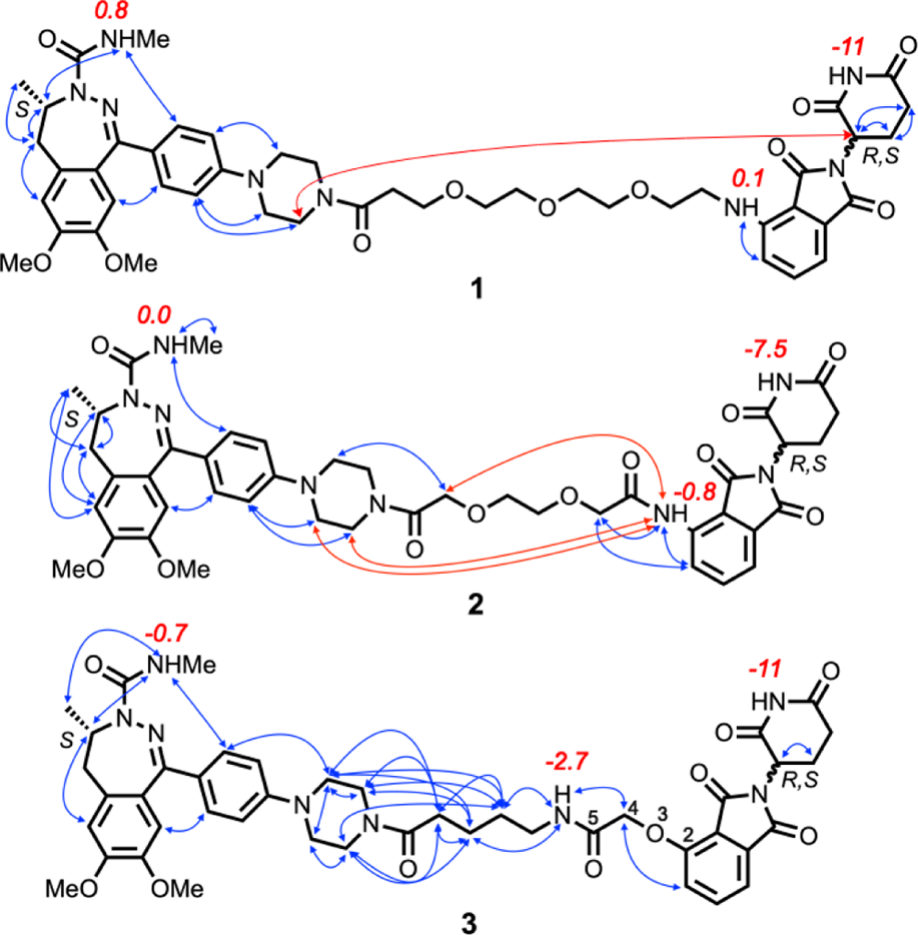
Overview of experimentally determined proton–proton distances that were used to determine the solution conformations of PROTACs **1–3**. Red arrows indicate long-range NOEs between protons in the BRD4 and CRBN ligands, while all other NOEs are indicated by blue arrows. Temperature coefficients (Δδ/*T*, ppb/K) for the NH protons of **1–3** in CDCl_3_ are given in red adjacent to each NH proton. An absolute value for Δδ/*T* of <3 indicates the NH to be involved in a strong intramolecular hydrogen bond (IMHB) or shielded from the solvent, while larger values indicate that the environment of the NH varies with the temperature.^37^ Atoms 2–5 have been labeled for PROTAC **3**.

Chloroform was used in the NMR studies of **1–3** since it has a dielectric constant (ε = 4.8) similar to that of a lipid bilayer (ε = 3.0).^36^ The proton resonances of the piperazine moiety of **1–3** were broad at room temperature, most likely due to a slow conformational exchange of this moiety. However, sharp resonances were observed at −35 °C, indicating the slower dynamics of the piperazine ring, and spectra acquired at this temperature were therefore used for the NAMFIS analysis. Compounds **1** and **2** show long- and medium-range NOEs between the thalidomide moiety and the BRD4 ligand or linker, indicating that they adopt folded conformations in solution (Figure 2). PROTAC **3** lacks such NOEs and also has too few NOEs to define the conformations about the three rotatable bonds adjacent to the thalidomide moiety, that is, the bonds connecting atoms 2–5 (Figure 2). This prevented the determination of the solution ensemble for **3**. However, the lack of long- and medium-range NOEs for **3** indicates that it adopts more elongated and less-folded conformations in chloroform than **1** and **2**.

Variable-temperature (VT) NMR spectroscopy^37^ showed that the urea-type NH of the BRD4 ligand is shielded from the surrounding solution or involved in a strong IMHB in all three PROTACs (Figure 2). This is also the case for the anilinic NH in **1** and **2**, whereas the amide NH in the linker of **3** appears to be involved in a moderately strong IMHB. The temperature coefficients indicate the thalidomide NH of all three PROTACs is involved in weak IMHBs that are broken up with increasing temperature or that shielding is reduced as the temperature increases.

### Description of Solution Ensembles

The thalidomide moiety was incorporated in a racemic form in **1–3,** resulting in them being studied as 1:1 diastereomeric mixtures (Figure 2; confirmed by chiral chromatography for **1** and **2**, Supporting Information Figures S2 and S4). The ^1^H and ^13^C NMR spectra of **1–3** showed only one set of resonances (Supporting Information Figures S13–S31), revealing that the environment around one chiral center was not affected by the other, distant center. The affinity of the *S*-form of thalidomide is 1 order of magnitude higher for CRBN than that of the *R*-form,^38^ suggesting the *S*,*S*-form of **1–3** to be more important for biological activity. To avoid neglecting any important information, we still determined the solution ensembles for both the *S*,*S*- and *S*,*R*-forms of **1** and **2** by fitting theoretical ensembles to the experimentally determined interproton distances. Akaike information criteria^39^ (AIC) analyses suggested that the two diastereomeric ensembles of **1** and **2** fit equally well with the NMR data (Table S14). This is in line with the two chiral units not influencing each other’s orientation due to the flexibility of the linker that connects them.

**Figure 3:**
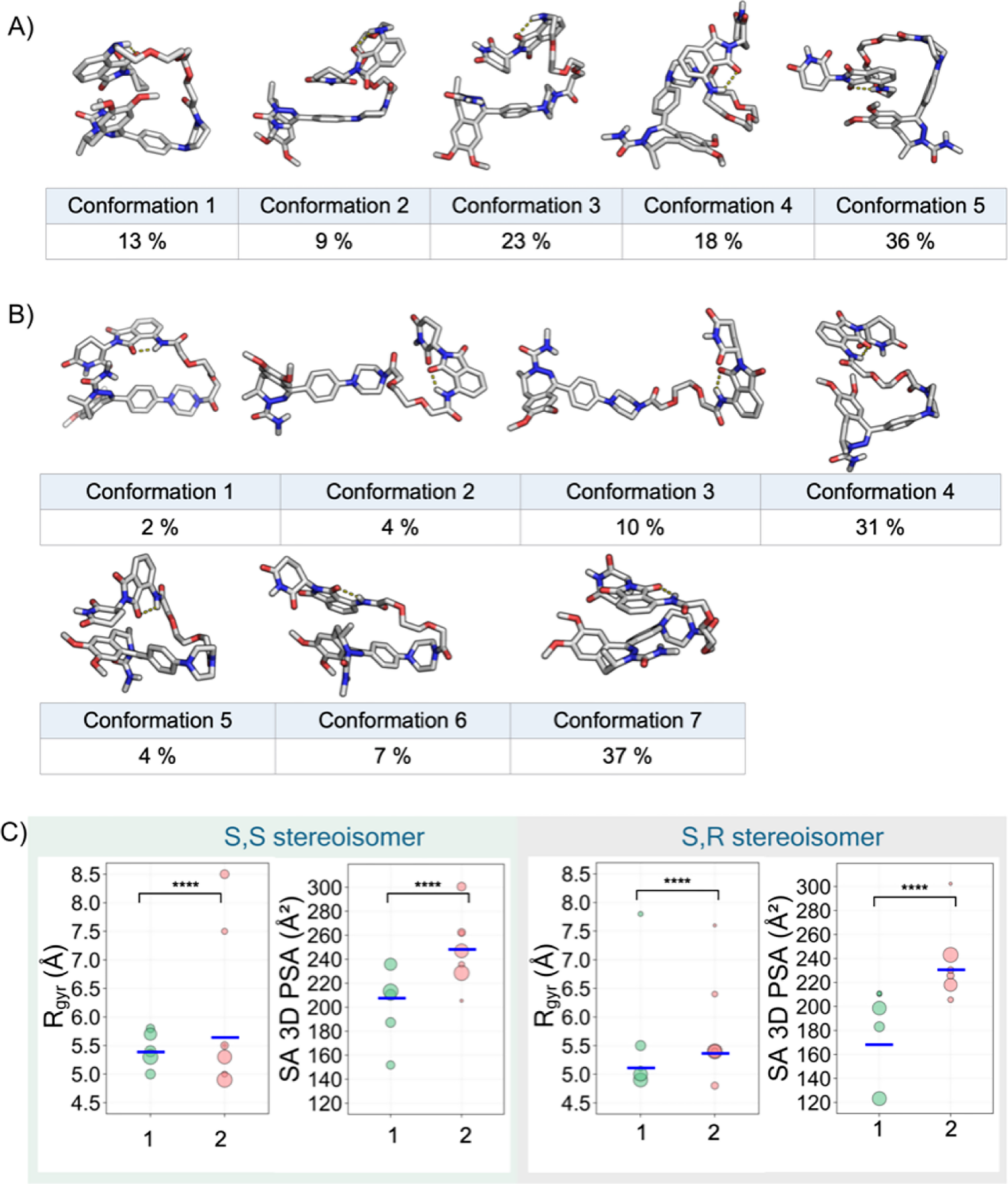
(A,B) Conformational ensembles of the *S*,*S*-stereoisomers of PROTACs **1** and **2** in CDCl_3_ determined by NAMFIS analysis. The number and population (in %) are provided for each conformation. IMHBs are indicated by yellow dotted lines. (C) Radius of gyration (*R*_gyr_) and solvent-accessible 3D polar surface area (SA 3D PSA) for the solution ensembles of the *S*,*S*- and *S*,*R*-stereoisomers of **1** and **2** in CDCl_3_. The area of each circle is proportional to the population (in %) of the corresponding conformation. Population-weighted mean values are shown as blue horizontal bars. Wilcoxon test *p*-values: **** ≤ 0.0001.

The chloroform ensemble of *S*,*S*-**1** determined by using the NAMFIS algorithm was represented by five conformations, with populations ranging from 9 to 36% (Figure 3A, cf. Figure S9 for the ensemble of *S*,*R*-**1**). The conformations of *S*,*S*-**1** were all folded with the backbone adopting two turns. For *S*,*S*-**2**, the solution ensemble consisted of seven conformations, four of which were minor (<10%), while the population of the three major conformations ranged from 10 to 37% (Figure 3B, cf. Figure S10 for the ensemble of *S*,*R*-**2**). The ensemble of *S*,*S*-**2** displayed greater structural diversity than that of *S*,*S*-**1**, with *S*,*S*-**2** having conformations which were folded with one (no. 1 and 6) or two turns (no. 4, 5, and 7) or were essentially linear (no. 2 and 3). All conformations of *S*,*S*-**1** and *S*,*S*-**2** had an IMHB between the NH at the linker attachment point and the adjacent carbonyl oxygen in the thalidomide moiety. This agrees with the low-temperature coefficients observed for these amide protons by VT NMR spectroscopy (Figure 2). In addition, conformations 5 (36%) of *S*,*S*-**1** and 6 (7%) of *S*,*S*-**2** were stabilized by π–π interactions between the thalidomide moiety and the dimethoxylated phenyl ring of the BRD4 ligand. Further analysis^40^ of the non-covalent intramolecular interactions revealed that all conformations of *S*,*S*-**1** and all but the two linear conformations of *S*,*S*-**2** were also stabilized by van der Waals interactions between the aromatic moieties in the ligands and aliphatic and/or polar groups in the linker or in the other ligand (Figures S11 and S12). The solution ensembles of *S*,*R*-**1** and *S*,*R*-**2** were very similar to those of the *S*,*S*-forms of **1** and **2** (Figures S9 and S10). Again, the conformations of *S*,*R*-**1** had a higher degree of folding than those of *S*,*R*-**2,** and all conformations possessed the IMHB between the linker NH and the adjacent thalidomide carbonyl group. The two major conformations of *S*,*R*-**1** and the major one of *S*,*R*-**2** were stabilized by π–π interactions, while van der Waals interactions involving the aromatic groups were frequent just as in the *S*,*S*-forms.

The polarity and the size of the permeating conformation(s) are two key properties that determine the permeability of a compound across a cell membrane.^41^ The SA 3D PSA is an established descriptor of polarity,^31^ while the size is approximated by the radius of gyration (*R*_gyr_).^42^ The larger degree of folding of the conformations of *S*,*S*-**1** resulted in an ensemble characterized by lower values of *R*_gyr_ and SA 3D PSA than for the structurally more diverse ensemble of *S*,*S*-**2** (Figure 3C). Population-weighted mean values for *R*_gyr_ and SA 3D PSA were 5.42 Å and 209 Å^2^ for *S*,*S*-**1** and 5.58 Å and 246 Å^2^ for *S*,*S*-**2**, respectively (Table S16). The ensembles of the *S*,*R*-forms of **1** and **2** displayed similar differences (Figure 3C, Table S16). The descriptors calculated for the solution ensembles of **1** and **2** in chloroform thus rationalize the differences in permeability observed for these two PROTACs. PROTAC **3** can be expected to populate even more elongated conformations, having even higher values for *R*_gyr_ and SA 3D PSA. In conclusion, the ability to adopt folded conformations, which minimize *R*_gyr_ and SA 3D PSA, appears to be important for these CRBN PROTACs to enter cells.

### MD Simulations

We performed unrestrained MD simulations to obtain further insights into the conformational space populated by PROTACs **1–3** in addition to that obtained by NMR spectroscopy for **1–2**. The simulations were carried out for 100 ns after initial energy minimization, thermalization, and equilibration. Explicit chloroform was used as the dielectric constant of chloroform is close to that of the interior of a cell membrane^36^ and to allow comparison to the solution ensembles determined by NAMFIS for **1** and **2**. The MD simulations were performed only for the *S*,*S*-stereoisomers of **1–3** as the higher affinity of *S*-thalidomide for CRBN makes them more likely to be more important for the PROTAC’s biological activity than the *S*,*R*-enantiomers. Three replicates were performed for each PROTAC to avoid any incorrect conclusions that can be drawn from single simulations (Figure S32).^43^ The simulations converged within 5–10 ns, and the variation between the replicates was small for PROTACs **2** and **3**, which have shorter linkers, and somewhat larger for **1**, reflecting its longer linker (Figure S32).

**Figure 4:**
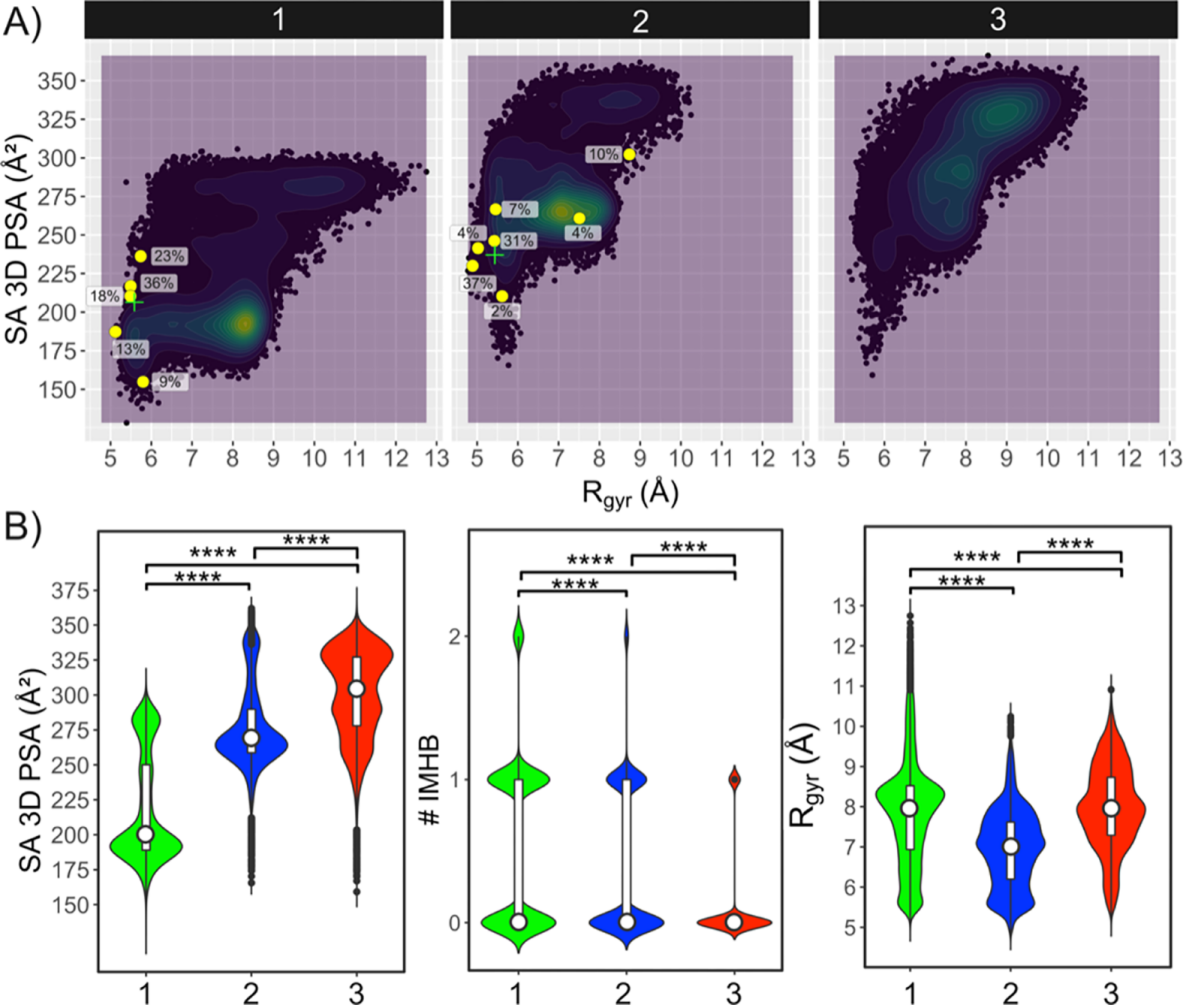
Descriptors calculated from the MD simulations for the *S*,*S*-stereoisomers of PROTACs **1–3** in explicit chloroform. (A) Chemical property space populated by **1–3** as revealed by plotting of the SA 3D PSA vs the *R*_gyr_ of the conformations from the MD simulations. Densely populated property space is colored green to yellow. The conformations determined by NAMFIS for *S*,*S*-**1** and *S*,*S*-**2** are shown as yellow circles, and their population in % is given adjacent to each circle. The green cross signs indicate the population-weighted mean values of the SA 3D PSA and *R*_gyr_ in the ensembles determined by NAMFIS for *S*,*S*-**1** and *S*,*S*-**2**. (B) Distribution of the SA 3D PSA (left), the number of IMHBs (# IMHB, center), *R*_gyr_ (right) of the conformations from MD simulations. White bars and circles indicate the 25th to 75th percentiles and the mean values, respectively. Wilcoxon test *p*-values: **** ≤ 0.0001.

The analysis of the MD simulations for the three PROTACs revealed that the SA 3D PSA of the most populated conformational regions increased from 190 to 265 Å^2^ for **1** and **2**, respectively, to 290 and 330 Å^2^ for **3**, which has two highly populated regions (Figures 4, S33 and S34). The exposure of the larger SA 3D PSA was inversely correlated to the number of IMHBs formed by the PROTACs (Figure 4B, Table S20, Figure S35). The trends displayed by both descriptors are thus in excellent agreement with the decreasing cell permeability observed for **1–3** (Table 2). PROTAC **1** mainly populated conformations characterized by a *R*_gyr_ just over 8 Å (Figures 4B, S33). A local minimum with more compact conformations having a *R*_gyr_ of 5.6 Å and a SA 3D PSA similar to that of the global minimum at 190 Å^2^ was also observed in the simulations (Figures 4, S33). PROTAC **2** had a wide global minimum centered at a *R*_gyr_ just over 7 Å and a significant local minimum at 5.5 Å both at a SA 3D PSA of 265 Å (Figure 4B). The higher *R*_gyr_ found for **1** in its simulated minimum, as compared to that of **2**, most likely originates from the fact that the linker of **1** is close to 50% longer than that of **2** and could also be a result of the somewhat larger variation between the three replicate simulations for **1** (Figure S32). According to the MD simulations, PROTAC **3** populated an extended region of conformational space with conformations that were more elongated (*R*_gyr_ 7–9.5 Å, Figures 4B and S33) than those of **2** (which has an equally long linker). The simulations thus agree well with the fact that no long- or medium-range NOEs were observed for **3**, in contrast to those observed for **1** and **2** (Figure 2). As discussed in greater detail below, inspection of the MD trajectories of **1–3** showed that extended conformations may have higher SA 3D PSAs and fewer IMHBs, while the opposite could be seen for folded conformations (Figures S37–S39). Just as for the experimentally determined ensembles of **1** and **2,** the MD simulations revealed that some conformations of **1–3** were stabilized by π–π interactions.

The conformations determined for PROTACs **1** and **2** by NAMFIS analysis fall within the chemical property space predicted for the two PROTACs by the MD simulations (Figures 4A, S33). In addition, the population-weighted mean SA 3D PSA values for the experimentally determined ensembles of **1** and **2**, which constitute the center of gravity of these ensembles, match the PSA values of the densely populated regions in the MD simulations reasonably well. The *R*_gyr_ for the local minima in the simulated ensembles of **1** and **2** come close to the population-weighted mean values from NAMFIS for **1** and **2**, whereas the global minima from MD have higher *R*_gyr_. In conclusion, just as for the NMR studies, the MD simulations found that the propensity to adopt folded conformations with a low SA 3D PSA correlated with the differences in cell permeability displayed by PROTACs **1–3**. However, the more densely populated regions found by MD had higher *R*_gyr_ than those identified by NAMFIS for **1** and **2**.

### Structural Analysis of the Conformations from MD Simulations

**Figure 5:**
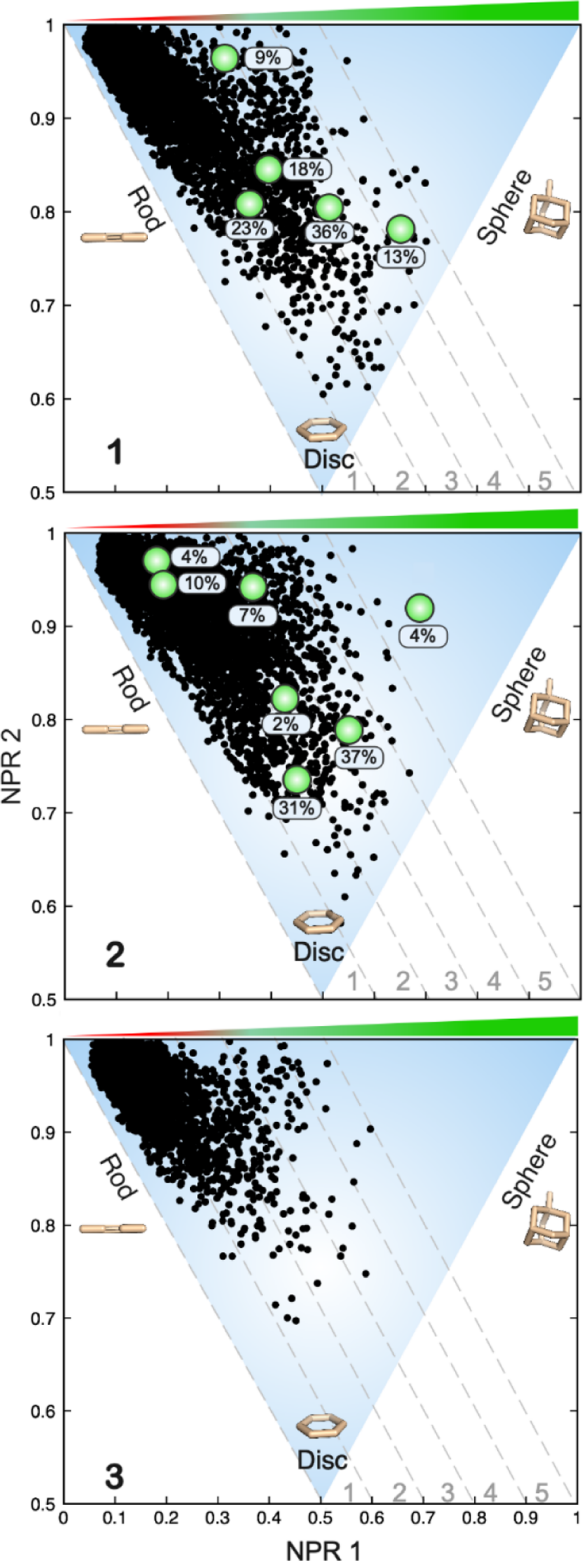
PMI plots characterizing the degree to which conformations from the MD simulations of **1–3** adopt rod-, disc-, and sphere-like shapes. The green circles in the PMI plots for **1** and **2** indicate the shape of each of the conformations determined by NAMFIS for **1** and **2**. The population in % is given adjacent to each green circle. NPR1: normalized PMI ratio 1; NPR2: normalized PMI ratio 2.

Principal moments of inertia (PMI) analysis of the conformations from the MD simulations of **1–3** indicated that the high- and medium-permeable PROTACs **1** and **2** adopt conformations that are more disc-like and spherical than those of the low-permeable **3** (Figure 5, Table S21). However, the longer linker of **1** as compared to **2** complicates the comparison of the shapes of these two PROTACs. It is notable that the experimentally determined conformations of **1** and **2** are located within the PMI space described by the MD simulations, with the exception of one minor conformation (4%) for PROTAC **2**.

**Figure 6:**
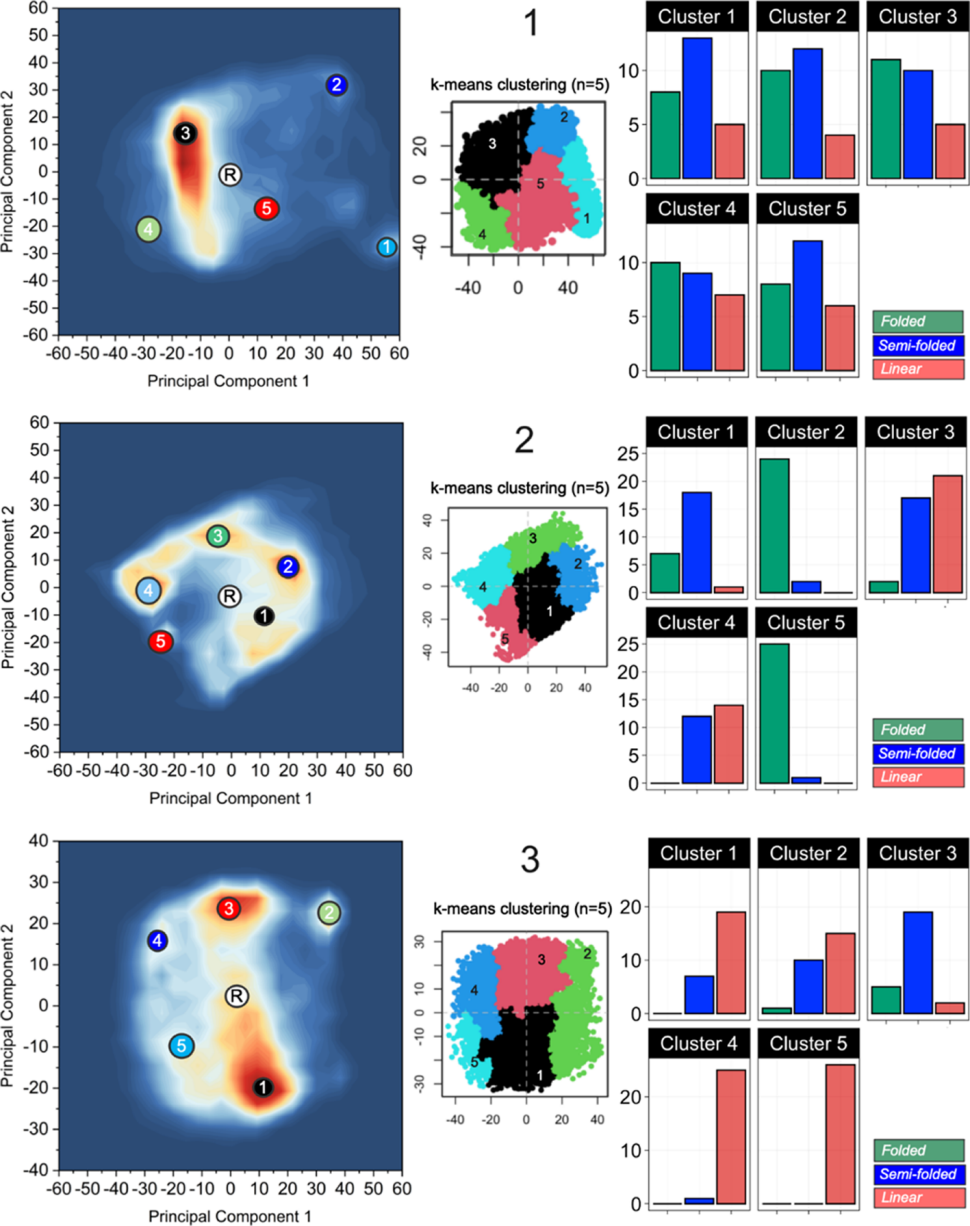
Clustering of conformations from the MD simulations of **1–3** by PCA of their 3D structures. Densely populated regions of conformational space are indicated in red, less populated regions are in white, and regions not populated are indicated in blue. The regions belonging to each cluster are visualized in the center of each panel. The bar charts inserted at the right of each panel show the classification of 26 representative conformations from each cluster as being folded, semi-folded, or linear. The starting conformation for the MD simulations of each PROTAC is indicated by an encircled R.

A more in-depth analysis of the structural relationship of all the conformations from the MD simulations of **1–3** was performed by principal component analysis (PCA) to provide five structural clusters for each PROTAC (Figures 6, S40–S42). A subset of 26 diverse conformations was then selected from each cluster in an additional PCA, followed by manual analysis and classification of each conformation as being folded with the backbone adopting two turns, semi-folded with one turn, or linear (Figures 6, S43, Table S22). This analysis provided a representative description of the structural diversity of the conformations found in the conformational space sampled by the MD simulations for each PROTAC. It revealed that PROTAC **1** predominantly adopted folded and semi-folded conformations in each of the five clusters (Figure 6A). In the most populated cluster (no 3), the proportion of folded conformations, which resemble those determined experimentally by NAMFIS analysis, was somewhat larger than the semi-folded conformations. PROTAC **2** was described by four equally populated clusters and one minor one (Figure 6B). These clusters showed greater structural variation than **1**, with two clusters being dominated by folded conformations, one by semi-folded, and the remaining two having equal populations of semi-folded and linear conformations. This conformational diversity agrees well with the experimental ensemble of **2**. Linear conformations dominated in the major and the three minor clusters of PROTAC **3**, while the medium-populated cluster (no 3) was characterized by semi-folded conformations. The conformations simulated by MD for **3** are thus more elongated than those in the ensembles of **1** and **2**, as expected from the lack of long- or medium-range NOEs in the NMR spectra of **3**.

**Figure 7:**
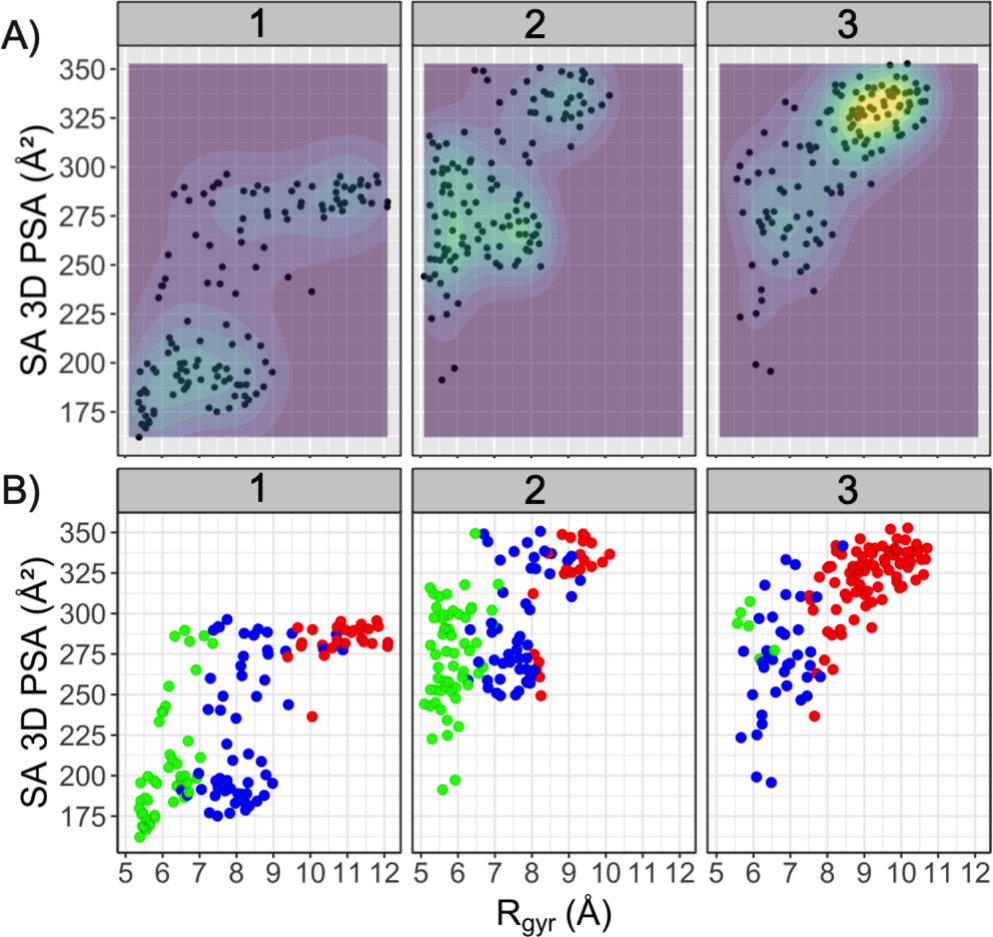
(A) Overview of the distribution of the diverse subset of 26 conformations in the five structural clusters of each PROTAC in chemical property space defined by the *R*_gyr_ and SA 3D PSAs. Regions of property space that display greater structural diversity, that is, which are more densely populated by the selected conformations, are indicated by green-yellow color and by the contour lines. (B) Distribution of conformations with different folds in the chemical property space. Folded conformations are in green, semi-folded in blue, and linear conformations are in red, just as in Figure 6. Note that some conformations populate identical property space and are therefore superimposed in panels A and B.

Recent studies of relatively rigid drugs in the bRo5 space and a more flexible PROTAC have shown that relationships between the type of folding of different conformations and *R*_gyr_ on the one hand and SA 3D PSAs on the other hand exist but can be complex, in particular for correlations to the SA 3D PSA.^35,44^ We calculated the *R*_gyr_ and SA 3D PSAs for all 26 conformations in the five clusters for each PROTAC to investigate (i) to what extent there is a relationship between the five structural clusters of each PROTAC and chemical property space, and (ii) in what property space the three different folds of each PROTAC are located (Figures 7, S44). As might be expected from the structural heterogeneity displayed by many of the five clusters, different clusters were often located in overlapping regions of property space defined by *R*_gyr_ and SA 3D PSAs (Figure S44). This is, for instance, the case for the five clusters of PROTAC **1**, all of which are composed of a mixture of folded, semi-folded, and linear conformations. However, clusters mainly composed of folded conformations, such as clusters 2 and 5 of PROTAC **2**, had lower *R*_gyr_ and SA 3D PSAs than clusters 3 and 4, which consisted of semi-folded and linear conformations. A similar trend was observed for the semi-folded cluster 3 of PROTAC **3** as compared to the linear clusters 4 and 5. However, a clearer relationship was found between the three types of folding of the conformations of each PROTAC and their location in property space (Figure 7). Conformations classified as folded were found at low *R*_gyr_, while semi-folded and linear conformations had intermediate and high *R*_gyr_, respectively. As observed recently,^35^ the relationship between folding and SA 3D PSA was less clear; conformations having the same fold displayed major differences in the SA 3D PSA.

The location of the three classes of conformations in chemical property space revealed that the most densely populated region in the MD simulations of PROTAC **1** consists of semi-folded conformations (Figures 7B and 4A), while folded conformations populate the local minimum (*R*_gyr_ 5.6 Å, SA 3D PSA 190 Å^2^). Similarly, semi-folded and folded conformations populate the global and local minima of PROTAC **2**, respectively. However, even though the conformations in the minima of **2** have the same overall fold as that of **1**, the conformations of **2** have a much higher SA 3D PSA. In contrast, the conformations of the two most populated regions of property space for **3** mainly consist of linear conformations that differ in *R*_gyr_ and SA 3D PSAs. As judged by their location in chemical property space and overall folding, the conformations in the simulated local minima of **1** and **2** resemble the ensembles determined by the NAMFIS algorithm for these PROTACs. The more densely populated regions of **1** and **2** from the MD simulations differ in the overall folding as compared to the conformations from NAMFIS, but both methods rank the polarity of the ensembles in the same order.

### Origins of PROTAC Folding

Comparison of the root mean square fluctuation (RMSF) of the atoms in PROTACs **2** and **3**, which have linkers of equal length, suggested the linker in **3** to be somewhat more flexible than the one in **2** (Figure S45). Presumably, this increase in flexibility constitutes one likely explanation for why **3** adopts a larger proportion of entropically favored, linear conformations than **2**. In addition, the gauche effect^45^ will favor turns in the PEG-like linker of **2** in contrast to **3** for which anti-conformations will be preferred for the single bonds of the alkyl linker. The PEG linker of PROTAC **1** displayed a flexibility similar to that of **3** (Figure S45), which most likely originates from the fact that it is longer than the linker in **2**. We propose that this flexibility, in combination with the gauche effect, allows **1** to adopt the highest degree of folded conformations with a low SA 3D PSA. Last but not least, the propensity of the conformations of **1** to be stabilized by a higher degree of IMHBs (Figure 4B) and van der Waals interactions (Figures S11 and S12) than **2** and **3** contributes to the high degree of folding displayed by **1**.

## Conclusions

We have investigated the origin of differences in cell permeability displayed by three highly flexible CRBN-based PROTACs that differ in the length and composition of their linkers but otherwise have identical structures. Independently of each other, NMR spectroscopy and MD simulations revealed that the propensity of the PROTACs to adopt folded and semi-folded conformations with a low SA 3D PSA in chloroform correlated to higher cell permeability. Conformational ensembles determined by NMR spectroscopy for **1** and **2** had a lower *R*_gyr_, that is, they were more folded than those from the MD simulations where semi-folded conformations dominated in the densely populated regions. Both methods suggested that semi-folded and folded conformations were stabilized by IMHBs, π–π interactions, and van der Waals interactions.

The length, chemical nature, and flexibility of the linker were essential for allowing the PROTACs to adopt folded conformations with a low SA 3D PSA that correlate to high cell permeability. The studies of **1–3** allow us to propose guidelines for choosing linkers in order to enhance cell permeability. Linkers that contain centrally located amide bonds may enforce a higher degree of elongated conformations having a high SA 3D PSA to a PROTAC and thus appear less suitable for incorporation into PROTACs. The gauche effect of PEG-type linkers most likely contributes to a larger proportion of folded conformations, making them more attractive than alkyl linkers, which induce a higher proportion of elongated anti-conformations.

Our results are of particular relevance for the design of cell-permeable CRBN-based PROTACs, which is the first class of PROTACs to reach the clinic. However, it is likely that cell permeability will also be elevated for members of other classes of PROTACs that can adopt folded conformations with a low SA 3D PSA. Our work also suggests that MD simulations in explicit chloroform can be used for the prospective, qualitative ranking of cell permeability in the design of PROTACs.

## Experimental Section

### Synthesis and Characterization of PROTACs **1–3**

The synthesis of PROTACs **1–3** has been reported previously,^25^ and their purity was determined to be >95% by liquid chromatography–mass spectrometry prior to use (Figures S1, S3 and S5). PROTACs **1** and **2** were determined to be 1:1 mixtures of the *S*,*S*- and *S*,*R*-diastereomers by chiral high-performance liquid chromatography (Figures S2 and S4).

### Aqueous Solubility and log *D*

The aqueous solubility and log *D*_7.5_ of **1–3** were determined as reported previously.^46^

### BRD4 Bromodomain Interaction Assays

The potency (IC_50_) of **1–3** as inhibitors of the BD1 and BD2 domains of BRD4 in a biochemical assay was determined as reported previously.^25^

### Biochemical CRBN Assay

Compound binding to the CRBN–DDB1 complex was measured in a TR-FRET assay format using FLAG-tagged DDB1 and His-tagged CRBN in the complex at a concentration of 5 nM. Final concentrations of 20 nM of the Cy5-labeled thalidomide tracer and 0.25 nM of LANCE Eu-W1024-anti-6× HIS antibody were used for detection. Compounds were tested in duplicates at up to 11 concentrations, and black assay plates (Greiner) were predispensed with the respective compound [total dimethyl sulfoxide (DMSO) concentration below 1% vol/vol, typically at 50 nL]. An antibody–enzyme solution mix was prepared at a 0.625 and 12.5 nM concentration (2.5-fold higher concentration with respect to the final concentration), respectively, in 1× assay buffer (50 mM 4-(2-hydroxyethyl)-1-piperazineethanesulfonic acid pH 7.5; 150 mM NaCl; 1 mM dithiothreitol ; 0.005% Tween-20; 0.01% bovine serum albumin) and left on ice until further use. In addition, a tracer solution was prepared at a 33.3 nM concentration (1.67-fold) in 1× assay buffer. The final assay volume was 5 μL and consisted of 2 μL of the antibody–enzyme mix and 3 μL of the tracer solution added to the predispensed compound plates per well. The mix was incubated for 60 min prior to measuring the FRET signal with an appropriate HTRF module using a BMG Pherastar plate reader. As an inhibitor control, the appropriate amount of DMSO was added instead of the compound, and the enzyme was omitted in the antibody–enzyme mix. As a neutral control, the appropriate amount of DMSO was added instead of the compound, whereas all other reagents remained the same. For analysis, the data were normalized against controls and analyzed using the GeneData software.

### Cell-Based CRBN Assay

Compound binding to CRBN in cells was determined using the NanoBRET in-cell CRBN kit from Promega as described previously.^25^

### PAMPA Permeability

The permeability of **1** and **3** in the PAMPA was determined at Pharmaron.^47^

### Caco-2 Cell Permeability

The permeability of **1–3** across Caco-2 cell monolayers in the AB and BA directions was determined as described previously.^46^

### NMR Spectroscopy

The NMR spectra of PROTACs **1–3** were recorded in CDCl_3_ at −35 °C on an 800 MHz Bruker Avance III HD NMR spectrometer equipped with a TXO cryogenic probe. The compounds were assigned using ^1^H, ^13^C, TOCSY, NOESY, HSQC, and HMBC NMR spectra (Figures S13–S31). NOESY buildups were recorded with mixing times of 100, 200, 300, 400, 500, 600, and 700 ms, with 16 transients and 512 and 2048 points collected in the indirect (F1) and direct (F2) dimensions, respectively. The relaxation delay *d*_1_ was set to 2.5 s, and the spectra were processed using the software MestReNova version 14.2.1. Normalized NOE peak intensities were calculated by the normalization of both cross peaks to both diagonal peaks of the protons showing NOE transfer according to the equation ([cross peak1 × cross peak2]/[diagonal peak1 × diagonal peak2])^0.5^.^48^ To calculate the interproton distances, initial rate approximation^49^ was used. Thus, NOE buildup rates were calculated from the NOEs that showed the linear intensity increase as a function of the mixing time, as a rule for at least four consecutive mixing times (*r*^2^ > 0.95). The distances were calculated according to the equation *r*_*ij*_ = *r*_ref_(σ_ref_/σ_*ij*_)^(1/6)^ using the distance between geminal methylene protons (1.78 Å) as the internal distance reference. Further details are provided in the Supporting Information.

### Theoretical Conformational Ensembles

Theoretical conformational ensembles of the *SR* and *SS* stereoisomers of PROTACs **1–3** were generated using unrestrained Monte Carlo conformational sampling. To ensure that the entire conformational space available for the compounds was sampled, the conformational search was done in parallel using five different force fields OPLS, OPLS-2005, OPLS4, Amber*, and MMFF, each in combination with the GB/SA implicit solvation models for water and chloroform. Each conformation was minimized using a maximum of 5000 iterative steps using the Polack–Ribiere conjugate gradient minimization scheme, as implemented in the BatchMin algorithm of MacroModel v 9.1 (Schrödinger Inc.).^50^ The number of torsion angles allowed to vary during each Monte Carlo step ranged from 1 to *n* – 1, where *n* equals the total number of rotatable bonds. Amide bonds were fixed in the trans configuration. All conformations within a 42 kJ/mol energy window from the global minimum were retained. The conformational searches fulfilled the equation 1 – (1 – (1/*N*))^*M*^ as an estimate of the probability that the conformational search is complete, where *N* is the total number of conformers and *M* is the number of search steps. The conformations obtained from the different conformational searches for each stereoisomer of **1–3** were combined, and redundant conformations were eliminated by applying a 3 Å RDMS cut-off (Tables S8–S10). The resulting ensembles were then used as theoretical input ensembles in the NAMFIS analysis.

### NAMFIS Analysis

The conformational ensembles of the *SR* and *SS* stereoisomers of compounds **1** and **2** were determined using the NAMFIS algorithm by fitting population-weighted, back-calculated interproton distances from conformations in the theoretical ensembles to those experimentally determined, following previously described protocols.^30^ Methylene (CH_2_) signals were treated according to the equation *d* = (((*d*_1_^–6^) + (*d*_2_^–6^))/2)^−1/6^ and methyl (CH_3_) signals according to *d* = (((*d*_1_^–6^) + (*d*_2_^–6^) + (*d*_3_^–6^))/3)^−1/6^. The output conformational ensembles were validated by comparison of the experimentally observed and back-calculated distances in terms of RMSD and by detection of no significant change in the ensembles by the addition of 10% random noise to the experimental data or upon random removal of individual experimental restraints. Further details of the NAMFIS analysis are provided in part 3 of the Supporting Information.

### MD Simulations and Trajectory Analysis

The structures of PROTACs **1–3** were built using the Maestro module of the Schrödinger suite.^50^ Correct chirality and protonation states were checked and fixed with the Epik tool (Schrödinger Release 2020),^51^ and the resulting structures were used as inputs for the geometry optimization and MD simulations.

The MD simulations were performed in triplicate for each PROTAC with the Amber software (version 18).^52,53^ Prior to MD simulations, the geometry of PROTACs (**1–3**) was optimized using Gaussian (version 16)^54^ with the HF method using 6-31G** basis sets. The geometries were further optimized using the M06-2X functional using 6-31+G** basis sets. The atomic charges for the PROTACs were assigned based on the electrostatic potential (ESP) fitting, using the RESP procedure as implemented in the Merz–Singh–Kollman scheme^55^ in the Antechamber tool.^56^ The ESP calculations for PROTACs (**1–3**) were carried out using the B3LYP/cc-pvTZ level of theory in a chloroform solvent environment. Chloroform was described using the integral equation formalism variant of the polarizable continuum model, which is called by the IEFPCM keyword in the Gaussian 16 (Rev. C.01) software.^54^ The force field parameters for the PROTACs were based on the general Amber force field (GAFF), and GAFF atom types were assigned by Antechamber. Solvent molecules (chloroform, ε = 4.8, frcmod.chcl3) were added to the PROTACs with a 30 Å buffering distance between the edges of the truncated octahedron box (approximately 3000 chloroform solvent molecules were added in the box). Subsequently, the tleap tool^53^ from the Amber package was used to build topology parameters and coordinate input files. Periodic boundary conditions were used to eliminate edge effects during MD simulations. MD simulations were performed in four stages—minimization, thermalization, equilibration, and production run. Energy minimization was performed in two steps. First, the system (ligand and explicit chloroform) was minimized using the steepest descent with all heavy atoms restricted for up to 1000 cycles. The second step, involving energy minimization of the entire system with no positional constraints, was applied for 200 cycles. Thermalization was initiated by generating starting velocities at 100 K from a Maxwell–Boltzmann distribution and progressively increasing the temperature to 300 K at a constant volume throughout a 200 ps MD simulation. After thermalization, the system was equilibrated at constant temperature (300 K) and pressure (1 bar) using the Berendsen coupling algorithm^57^ before performing another 500 ps MD simulation. Following the equilibration process, a 100 ns MD production cycle was initiated, and a total of 10,000 snapshots were retrieved and analyzed. All bonds involving hydrogen atoms were constrained using the SHAKE algorithm.^58^

Trajectories were analyzed using the *CPPTRAJ* module^59^ from the Amber tool. Trajectory analysis included the root mean square deviation (RMSD), *R*_gyr_, SA 3D PSA, IMHB analysis, and RMSF; cf. parts S6.1 and S6.2 of the Supporting Information. All plots from the trajectory analysis were created using the RStudio (version 1.3.959) and Origin Pro (version 9.8.0.200) software.

### Molecular Properties’ Calculations

The *R*_gyr_ and IMHBs were calculated using the *CPPTRAJ* module.^59^ The SA 3D PSA was calculated using PyMol (version 2) using a solvent probe radius of 1.4 Å and a partial charge threshold of >1.0, as previously described.^31^

### Principal Moments of Inertia

PMI plots were generated for the conformations from MD simulations (for **1–3**) and the conformations from NMR spectroscopy (for **1-2**). The 3D-descriptor-normalized PMI ratio 1 (NPR1) and normalized PMI ratio 2 (NPR2) were calculated from the MOE suite (version 2020.09).^60^

### Principal Component Analysis

PCA from the Bio3D tool^61^ was used to further investigate the relationship between the conformations from the MD trajectories of **1–3**.^61^ To this end, the K-mean clustering approach,^62^ which finds patterns in data by clustering similar data points together, was used for conformational clustering. The number of clusters was set to 5 for each PROTAC in order to provide a clear description of the populated conformational space (Supporting Information, part S6.4).

### Conformation Subset Selection

To further investigate the folding of conformations in each of the five clusters generated for each PROTAC, a subset of 26 conformations from each cluster was chosen based on the Diverse Subset tool from the MOE suite.^60^ Principal components (PC1 and PC2) from PCA were chosen as optional descriptors during the subset selection. Each conformation was manually analyzed and classified into any of the following categories: folded, semi-folded, and linear (Supporting Information, part S6.5).

## Supporting Information Available

Purity analyses, Caco-2 cell permeability data. NMR data, Monte Carlo molecular mechanic conformational search for **1–3**, NAMFIS conformational analysis of **1** and **2,** variable temperature NMR studies of **1–3,** NMR spectra for **1–3**, computational methods, and analyses of the MD simulations of **1–3**, including analyses of MD trajectories, property descriptors, molecular shapes, and noncovalent interaction analyses (PDF)
Molecular formula strings document with structures (SMILES codes), solubility, POI, and CRBN binding, as well as permeability data for **1–3** (CSV)

## Abbreviations

AIC: akaike information criteria
BRD4: bromodomain-containing protein 4
bRo5: beyond rule of 5
CRBN: cereblon
IMHB: intramolecular hydrogen bond
LPE: lipophilic permeability efficiency
MD: molecular dynamics
NAMFIS: NMR analysis of molecular flexibility in solution
NRotB: number of rotatable bonds
PAMPA: parallel artificial membrane permeability assay
PCA: principal component analysis
PMI: principal moments of inertia
POI: protein of interest
PROTAC: proteolysis-targeting chimera
*R*_gyr_: radius of gyration
SA 3D PSA: solvent-accessible 3D polar surface area
TPSA: topological polar surface area
VHL: Von Hippel-Lindau
VT: variable temperature
