# Supplementary material for: Linker-Dependent Folding Rationalizes PROTAC Cell Permeability
Source: J Med Chem. 2022 Sep 28;65(19):13029–40. doi: 10.1021/acs.jmedchem.2c00877 (PMC9574858; doi:10.1021/acs.jmedchem.2c00877)
Supplement: Supplementary file 1 — jm2c00877_si_001.pdf [file jm2c00877_si_001.pdf]

Supporting Information

Linker-dependent folding rationalizes PROTAC cell permeability

Vasanthanathan Poongavanam,[a,#] Yoseph Atilaw,[a,#] Stephan Siegel,[b] Anja Giese,[b] Lutz Lehmann,[c] Daniel Meibom,[c] Mate Erdelyi[a] and Jan Kihlberg[a,*]

[a] Department of Chemistry - BMC, Uppsala University, Box 576, 75123 Uppsala, Sweden

[b] Bayer AG, Drug Discovery Sciences, 13342 Berlin, Germany

[c] Bayer AG, Drug Discovery Sciences, 42113 Wuppertal, Germany

[#] V.P. and Y.A. made equal contributions to the manuscript.

Corresponding author

Jan Kihlberg, jan.kihlberg@kemi.uu.se, ORCID: 0000-0002-4205-6040

Table of Contents

1. Purity reports for PROTACs 1–3 3

2. Permeabilities across Caco-2 cell monolayers for PROTACs 1–3 6

3. Conformational analysis of PROTACs 1–3 in $CDCl_3$ solution 7

3.1. ^1H-NMR chemical shift assignment 7

3.2. Interproton distances 10

3.3. Monte Carlo molecular mechanics (MCMM) conformational search 13

3.4. Identification and characterization of solution ensembles using the NAMFIS algorithm 15

3.5. Akaike information criterion (AIC) analyses 22

3.6. Structural comparison of conformers in solution ensembles 23

4. Variable temperature NMR studies 25

5. NMR Spectra of PROTACs 1-3 27

6. Computational methods and analyses of MD simulations of 1–3 38

6.1. Trajectory analysis of conformational space for PROTACs 1-3. 38

6.2. Conformation-dependent molecular property space 38

6.3. Principal Moments of Inertia (PMI) analysis 45

6.5. Conformation subset selection 49

6.6. RMSF analysis 51

7. References 52

1. Purity reports for PROTACs 1–3

1.1. PROTAC 1

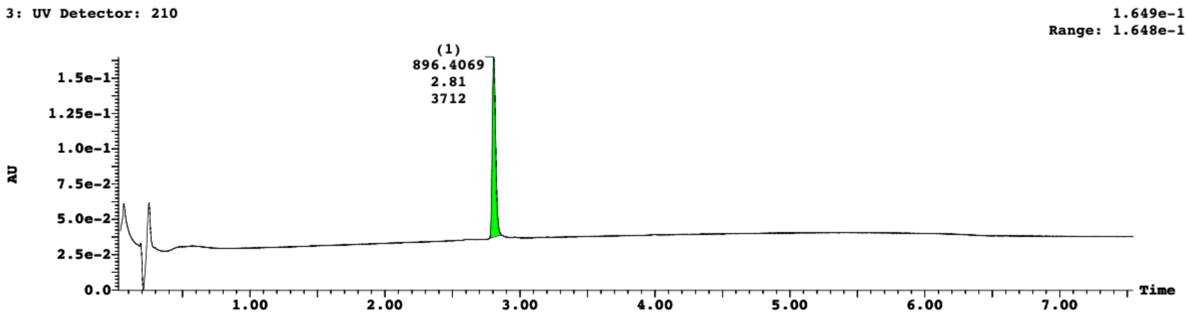

#	Spectrum	RT	Area%	Pos Neg UV	Mass Found
1	---	2.81	100.00	897, 208	896.4069

Figure S1. By reversed-phase HPLC. System MS: Waters TOF instrument; System UPLC: Waters Acquity I-CLASS; Column: Waters Acquity UPLC HSS T3 1.8 µm 50 x 1 mm; eluent A: 1 l water + 0.100 ml 99 % formic acid, eluent B: 1 l acetonitrile + 0.100 ml 99 % formic acid; gradient: 0.0 min 95 % A → 6.0 min 5 % A → 7.5 min 5 % A. Oven: 50 °C; flow: 0.35 ml/min; UV-Detection: 210 nm.

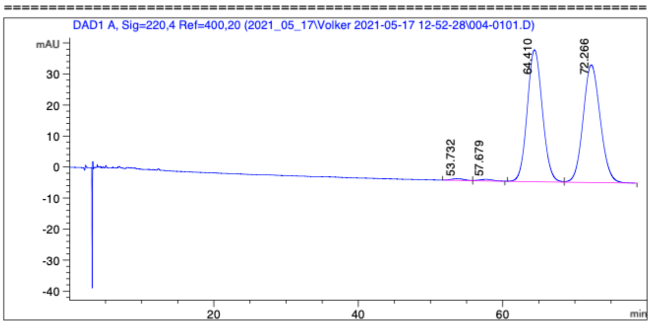

Report:

# Peak	RetTime [Min]	Area [mAU*s]	Area % [%]	Height [mAU]	Height [%]	Width [Min]	Signal Desc.
1	53.732	68.11	0.54	0.57	0.69	2.01	DAD1 A, Sig=220
2	57.679	69.79	0.55	0.53	0.65	2.20	DAD1 A, Sig=220
3	64.410	6202.28	49.27	42.54	52.16	2.43	DAD1 A, Sig=220
4	72.266	6247.77	49.63	37.92	46.50	2.75	DAD1 A, Sig=220

Figure S2. By chiral HPLC. Column : 250 x 4.6 mm, Daicel Chiralpak IC, 5 µm, flow : 1.0 ml/min, temperature: 70 °C, eluent : 0 % iso-hexane 100 % ethanol + 1 % H_2O + 0.2 % TFA; UV-Detection: 220 nm.

1.2. PROTAC 2

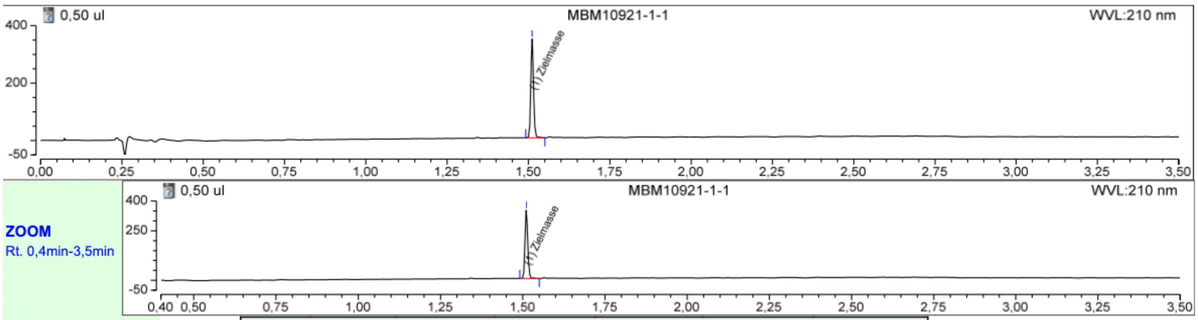

#	Spectrum	RT min	Area% 210nm	Area % UVMax	Zielmasse gefunden [M+H]+ Pos	[M-H]- Neg
1	Y	1,51	100,0	100,0	853,3516	851,3365

Figure S3. By reversed-phase HPLC. System MS: Thermo Scientific FT-MS; System UHPLC+: Thermo Scientific UltiMate 3000; column: Waters, HSST3, 2.1 x 75 mm, C18 1.8 µm; eluent A: 1 l water + 0.01 % formic acid; eluent B: 1 l acetonitrile + 0.01 % formic acid; gradient: 0.0 min 10 % B → 2.5 min 95 % B → 3.5 min 95 % B; oven: 50°C; flow: 0.90 ml/min; UV-Detection: 210 nm/ Optimum Integration Path 210-300 nm

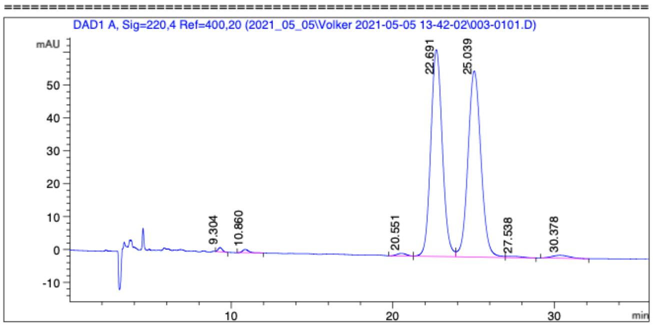

Report:

# Peak	RetTime [Min]	Area [mAU*s]	Area % [%]	Height [mAU]	Height [%]	Width [Min]	Signal Desc.
1	9.304	22.32	0.35	1.28	1.03	0.27	DAD1 A, Sig=220
2	10.860	26.81	0.42	1.02	0.82	0.44	DAD1 A, Sig=220
3	20.551	31.67	0.49	0.78	0.63	0.67	DAD1 A, Sig=220
4	22.691	3118.49	48.37	62.92	50.79	0.83	DAD1 A, Sig=220
5	25.039	3155.29	48.94	56.59	45.68	0.93	DAD1 A, Sig=220
6	27.538	27.85	0.43	0.41	0.33	1.12	DAD1 A, Sig=220
7	30.378	64.27	1.00	0.88	0.71	1.22	DAD1 A, Sig=220

Figure S4. By chiral HPLC. Column: 250 x 4.6 mm; Daicel Chiral ID, 5 µm; flow: 10 ml/min; temperature: 70 °C; eluent : 0 % iso-hexane 100 % ethanol + 1 % H_2O + 0.2 % TFA; UV-Detection: 220 nm.

1.3. PROTAC 3

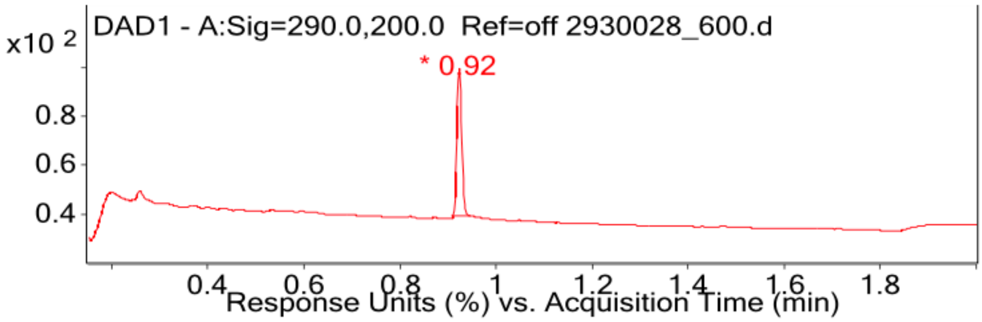

Integration Peak List

Peak	RT	Height	Area	AreaSumPercent
1	0.92	20.12	15	100

Figure S5. By reversed-phase HPLC. Instrument: Agilent 1290 UPLCMS 6230 TOF; column: BEH C 18 1.7 µm, 50x2.1mm; Eluent A: water + 0.05 % formic acid (99%); Eluent B: acetonitrile + 0.05 % formic acid (99%); gradient: 0-1.7 2-90% B, 1.7-2.0 90% B; flow 1.2 ml/min; temperature: 60°C; DAD scan: 190-400 nm.

2. Permeabilities across Caco-2 cell monolayers for PROTACs 1–3

Table S1. Caco-2 cell permeabilities for PROTACs **1–3**[a]

PROTAC	P_{app} AB[b] (nm/s)	P_{app} BA[c] (nm/s)	ER[d]
**1**	2.6 ±0.40	370 ±99	309 ±72
**2**	1.3 ±0.24	96 ±2.5	78 ±14
**3**	1.0 ±0.31	42 ±7.0	48 ±15

[a]Permeabilities across Caco-2 cell monolayers were determined a pH 7.5 and are mean values ±SEM from two or three repeats.

[b]Permeability in the apical-to-basolateral (AB) direction.

[c]Permeability in the basolateral-to-apical (BA) direction.

[d]Efflux ratio (P_{app} BA/P_{app} AB)

3. Conformational analysis of PROTACs 1–3 in $CDCl_3$ solution

3.1. ^{1}H-NMR chemical shift assignment

The assignment of PROTAC **1-3** were derived from ^{1}H, ^{13}C, TOCSY, NOESY, HSQC and HMBC NMR specta (Figures S11-S29), recorded at -35 °C on a 800 MHz BRUKER Avance III HD NMR spectrometer equipped with a TCI cryogenic probe using $CDCl_3$ as a solvent. ^{1}H NMR chemical shifts are listed in Table S2-S4.

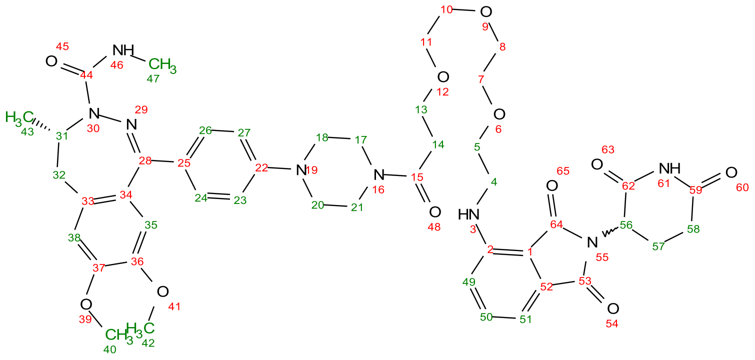

Figure S6: Structure and numbering of **1**

Table S2: ^{1}H-NMR chemical shift assignment (δ, ppm) for PROTAC **1** in $CDCl_3$

Proton no.	δ, $CDCl_3$	Proton no.	δ, $CDCl_3$
3	6.49	31	5.26
4	3.46	32`	2.73
5	3.69	32``	2.91
7	3.69	35	6.57
8	3.69	38	6.75
10	3.69	43	1.09
11	3.69	46	6.09
13	3.82	47	2.84
14	2.72	49	7.10
17	3.27	50	7.50
18	3.65	51	6.88
20	3.81	56	4.90
21	3.24	57`	2.09
23/27	6.91	57``	2.71
24/26	7.50	58`	2.73

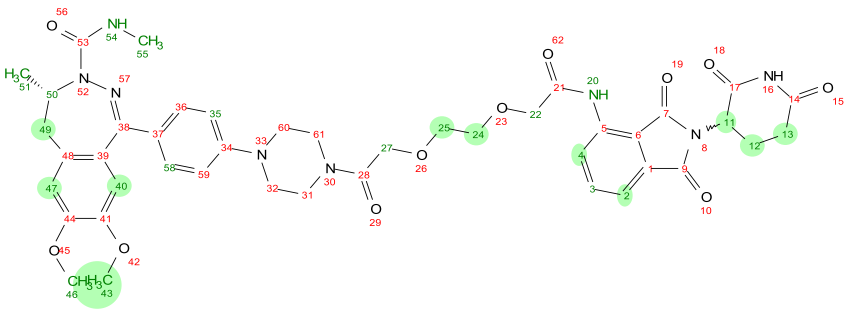

Figure S7: Structure and numbering of **2**

Table S3: ^{1}H-NMR chemical shift assignment (δ, ppm) for PROTAC **2** in $CDCl_3$

Proton no.	δ, $CDCl_3$	Proton no.	δ, $CDCl_3$
2	7.57	32	3.77
3	7.71	60	3.69
4	8.84	61	3.21
11	4.92	35/59	6.89
12`	2.16	36/58	7.45
12``	2.73	40	6.60
13`	2.74	47	6.74
13``	2.88	49`	2.74
20	10.47	49``	2.97
22	4.32	50	5.31
24	3.86	51	1.08
25	3.86	54	6.22
27	4.20	55	2.86
31	3.31		

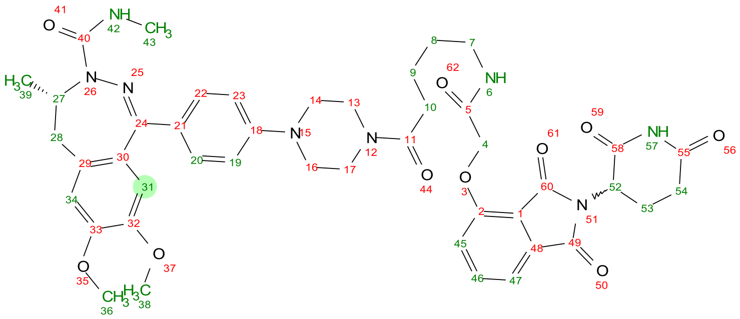

Figure S8: Structure and numbering of **3**

Table S4: ^{1}H-NMR chemical shift assignment (δ, ppm) for PROTAC **3** in $CDCl_3$

Proton no.	δ, $CDCl_3$	Proton no.	δ, $CDCl_3$
4`	4.58	27	5.27
4``	4.70	28`	2.72
6	7.77	28``	2.91
7	1.63	31	6.58
8	1.73	34	6.74
9	1.63	39	1.05
10`	2.38	42	6.14
10``	2.54	43	1.06
13	3.54	45	7.56
14`	3.13	46	7.77
14``	3.41	47	7.21
16	4.08	52	4.92
17	3.7	53`	1.93
19/23	6.97	54`	2.44
20/22	7.49		

3.2. Interproton distances

NOE build-ups were recorded without solvent suppression using mixing times of 100, 200, 300, 400, 500, 600 and 700 ms. The relaxation delay was set to 2.5 s, and 16 scans were recorded with 8192 and 512 data points in the direct (F2) and indirect (F1) dimensions, respectively. Interproton distances (r_{ij}) were calculated according to the equation $r_{ij}=r_{ref}(\sigma_{ref}/\sigma_{ij})^{(1/6)}$ using germinal methylene protons (ref=1.78 Å) as reference (Tables S5-S7). The NOE peak intensities were calculated according to the equation $([\text{cross peak1} \times \text{cross peak2}]/[\text{diagonal peak1} \times \text{diagonal peak2}])^{0.5}$. A minimum of 4 mixing times giving a linear ($R^2 \geq 0.94$) build-up rate (σ_{ij}) were used for quantification.

Table S5. Interproton distances (Å) for PROTAC **1** derived from NOE build-up measurements in $CDCl_3$ (δ in ppm)

No.	Proton A	Proton B	δA	δA	σ	R^2	Dis. r_{ij}(Å)
1	23/27	20	6.91	3.81	5.2247E-05	0.96	2.59
2	23/27	18	6.91	3.65	4.32237E-05	0.97	2.67
3	24/26	35	7.50	6.57	5.55108E-05	0.99	2.56
4	24/26	46	7.50	6.09	8.38775E-05	0.99	2.39
5	23/27	21	6.91	3.24	4.66834E-05	0.95	2.64
6	49	3	7.10	6.49	6.58365E-06	0.99	3.65
7	38	32``	6.75	2.91	0.000178969	0.99	2.11
8	38	32`	6.75	2.73	0.000127594	0.97	2.23
9	31	32``	5.26	2.91	0.000156402	0.98	2.16
10	31	32`	5.26	2.73	0.000173642	0.99	2.12
11	32`	43	2.73	1.09	3.34217E-05	0.95	2.79
12	46	31	6.09	5.26	3.90542E-06	0.96	3.99
13	56	58``	4.90	2.91	1.06529E-05	0.98	3.37
14	56	58`	4.90	2.73	4.14901E-05	0.99	2.69
15	56	57`	4.90	2.09	7.77696E-05	0.99	2.42
16	58``	57`	2.91	2.09	9.13102E-05	0.98	2.36
17	56	21	4.90	3.24	8.77092E-07	0.96	5.11

Table S6. Interproton distances (Å) for PROTAC **2** derived from NOE build-up measurements in $CDCl_3$ (δ in ppm)

No.	Proton A	Proton B	δA	δA	σ	R^2	Dis. r_{ij}(Å)
1	20	27	10.47	4.20	7.20505E-07	0.99	4.38
2	20	4	10.47	8.84	2.14324E-06	0.99	3.66
3	20	22	10.47	4.32	7.80124E-06	0.99	2.95
4	4	22	8.84	4.32	7.01187E-07	0.98	4.40
5	36/58	40	7.45	6.60	1.81244E-05	0.99	2.56
6	36/58	54	7.45	6.22	2.10097E-06	0.95	3.67
7	54	55	6.22	2.86	1.87303E-05	0.98	2.55
8	49``	50	2.97	5.31	1.86129E-05	0.96	2.55
9	49``	47	2.97	6.74	2.01649E-05	0.99	2.52
10	49``	51	2.97	1.08	7.3956E-06	0.99	2.97
11	47	51	6.74	1.08	1.79774E-06	0.99	3.76
12	47	50	6.74	5.31	2.47632E-06	0.94	3.57
13	35/59	32	6.89	3.77	3.59341E-06	0.99	3.35
14	35/59	31	6.89	3.31	3.95544E-06	0.99	3.30
15	27	60	4.20	3.69	9.5574E-06	0.96	2.85
16	20	31	10.47	3.31	6.77547E-07	0.95	4.43
17	20	32	10.47	3.77	9.76086E-07	0.99	4.17

Table S7. Interproton distances (Å) for PROTAC **3** derived from NOE build-up measurements in $CDCl_3$ (δ in ppm)

No.	Proton A	Proton B	δA	δA	σ	R^2	Dis. r_{ij}(Å)
1	6	4`	7.77	4.58	1.92872E-05	0.99	3.29
2	6	4``	7.77	4.70	1.68141E-05	0.98	3.36
3	6	9	7.77	1.63	3.05744E-05	0.99	3.04
4	6	8	7.77	1.73	2.96117E-05	0.99	3.06
5	45	4`	7.56	4.58	0.000164824	0.99	2.30
6	45	4``	7.56	4.70	0.00017094	0.99	2.28
7	52	53	4.92	1.93	0.00013531	0.99	2.37
8	20/22	31	7.49	6.58	4.16922E-05	0.99	2.89
9	20/22	42	7.49	6.14	5.77858E-05	0.99	2.74
10	34	27	6.74	5.27	1.75528E-05	0.99	3.34
11	42	27	6.14	5.27	6.98055E-06	0.99	3.89
12	42	39	6.14	1.05	2.21648E-06	0.99	4.71
13	10``	8	2.54	1.73	6.30554E-05	0.99	2.70
14	10`	8	2.38	1.73	8.0521E-05	0.99	2.59
15	10`	9	2.38	1.63	5.53285E-05	0.99	2.76
16	10``	9	2.54	1.63	5.09226E-05	0.99	2.79
17	20/22	14`	7.49	3.13	2.97886E-05	0.94	3.06
18	20/22	14``	7.49	3.41	2.31428E-05	0.95	3.19
19	13	10`	3.13	2.54	0.00014715	0.99	2.34
20	13	10``	3.54	2.54	0.000155142	0.99	2.32
21	14`	9	3.13	1.63	5.26644E-05	0.99	2.78
22	17	10`	3.70	2.38	8.64862E-05	0.99	2.56
23	16	14``	4.08	3.41	0.000147307	0.99	2.34
24	16	17	4.08	3.70	3.00994E-05	0.97	3.05
25	13	14``	3.54	3.41	0.000166079	0.99	2.29
26	13	14`	3.54	3.13	0.000201072	0.99	2.22
27	17	10``	3.70	2.54	0.000102	0.99	2.49
28	13	9	3.54	1.63	8.30332E-06	0.99	3.78
29	17	9	3.70	1.63	3.63067E-05	0.97	2.96
30	14`	10`	3.13	2.38	8.27767E-06	0.98	3.78
31	13	8	3.54	1.73	2.27E-05	0.97	3.20
32	17	8	3.70	1.73	2.83E-05	0.99	3.08
33	14`	8	3.13	1.73	2.89E-05	0.99	3.07

3.3. Monte Carlo molecular mechanics (MCMM) conformational search

The theoretical conformation ensembles of PROTAC **1-3** were identified by performing careful Monte Carlo conformational analysis using five different (OPLS, OPLS-2005, OPLS4, AMBER* and MMFF) force fields, each with the GB/SA solvation models chloroform and water.[1] These conformational searches were done using the Monte Carlo algorithm with intermediate torsion sampling, 50 000 Monte Carlo steps and a RMSD cut-off set to 2.0 Å. A Molecular Mechanics energy minimization was performed as implemented in the Macromodel BatchMin V12.1 of the Schrödinger Package. Each conformation was energy minimized using Polak-Ribière type conjugate gradient (PRCG) with a maximum of 5000 iterative steps. All conformations within 42 kJ/mol from the global minimum were saved. Results of all the different conformational searches are given in Table S8-S10. All ensembles generated by the conformational searches were combined and elimination of redundant conformations was performed by comparison of heavy atom coordinates applying an RMSD cutoff set to 3.0 Å giving the final ensemble used for NAMFIS-analysis.

Table S8. Result of the MCMM conformational analysis of **PROTAC 1**

	SS isomer				SR isomer		
Force fields	Solvent		RCE*	Force fields	Solvent		RCE
	$CHCl_3$	H_2O			$CHCl_3$	H_2O	
OPLS	274	502		OPLS	114	475	
OPLS4	564	895		OPLS4	800	1095	
OPLS 2005	706	1176	91	OPLS 2005	331	931	118
MMFF	1158	661		MMFF	1025	603	
AMBER*	516	695		AMBER*	622	717	
Final ensemble used			91	Final ensemble used			118

*RCE (Redundant Conformer Elimination)

Table S9. Result of the MCMM conformational analysis of **PROTAC 2**

SS isomer				SR isomer			
Force fields	Solvent		RCE	Force fields	Solvent		RCE
	$CHCl_3$	H_2O			$CHCl_3$	H_2O	
OPLS	347	255		OPLS	123	239	
OPLS4	580	555		OPLS4	367	468	
OPLS 2005	377	372	125	OPLS 2005	405	356	120
MMFF	408	525		MMFF	441	522	
AMBER*	398	329		AMBER*	304	357	
Final ensemble used			125	Final ensemble used			120

*RCE (Redundant Conformer Elimination)

Table S10. Result of the MCMM conformational analysis of **PROTAC 3**

SS isomer				SR isomer			
Force fields	Solvent		RCE	Force fields	Solvent		RCE
	$CHCl_3$	H_2O			$CHCl_3$	H_2O	
OPLS	184	505		OPLS	151	338	
OPLS4	660	711		OPLS4	513	550	
OPLS 2005	460	478	120	OPLS 2005	342	513	111
MMFF	650	586		MMFF	636	663	
AMBER*	537	634		AMBER*	484	460	
Final ensemble used			120	Final ensemble used			111

*RCE (Redundant Conformer Elimination)

3.4. Identification and characterization of solution ensembles using the NAMFIS algorithm

Solution ensembles were determined by fitting the experimentally measured distances and coupling constants to those back-calculated from computationally predicted conformations using the NAMFIS algorithm.[2-3] Methylene protons were treated according to the equation $d = (((d_1^{-6}) + (d_2^{-6}))/2)^{-1/6}$, and methyl protons according to $d = (((d_1^{-6}) + (d_2^{-6}) + (d_3^{-6}))/3)^{-1/6}$. The NAMFIS output solution ensembles were validated using standard methods, i.e. through evaluation of the reliability of the conformational restraints by the addition of 10% random noise to the experimental data, by the random removal of individual restraints, and by comparison of the experimentally observed and back-calculated distances.

Additional validation step was performed by comparing the solution ensembles for specific parts of PROTACs **1** and **2**, when feeding the NAMFIS algorithm with specific sets of NOE proton-proton distances, which describe certain parts of the molecule. Thus, the NAMFIS output obtained using all distances was compared to the NAMFIS outputs obtained using distances from the POI-ligand part only and the CRBN ligand part only. Using this, we evaluated whether long-range restraints (distances from POI ligand to CRBN ligand) over-restrained the compounds, possibly falsifying the conformation of parts of the molecule that are well-described by local NOE. Comparison of RMSD values and dihedral angles of these specific regions of the molecule ij ensembles derived from NAMFIS analysis including either restraints for a specific region only, or for the entire molecule indicated that the long-range restraints did not distort the conformation of the well-described (NOE, *J*) POI and CRBN parts of the molecule. The interatomic distances and populations of the validated conformational ensembles are given in Tables S11-S13.

Table S11. Experimentally determined and back-calculated distances (NAMFIS output) interproton distances (Å)

Interproton distances PROTAC **1**				Interproton distances PROTAC **2**			
**SS Isomer**		**SR Isomer**		**SS Isomer**		**SR Isomer**	
Exp.$_{dist(Å)}$	Calc.$_{dist(Å)}$	Exp.$_{dist(Å)}$	Calc.$_{dist(Å)}$	Exp.$_{dist(Å)}$	Calc.$_{dist(Å)}$	Exp.$_{dist(Å)}$	Calc.$_{dist(Å)}$
2.59	2.78	2.59	2.63	4.38	4.42	4.38	4.37
2.67	2.68	2.67	2.8	3.66	3.68	3.66	3.68
2.56	2.71	2.56	2.88	2.95	2.97	2.95	2.95
2.39	2.71	2.39	2.6	4.4	4.76	4.4	4.67
2.64	2.9	2.64	2.77	2.56	2.68	2.56	2.71
3.65	3.68	3.65	3.67	3.67	3.67	3.67	3.69
2.11	2.5	2.11	2.51	2.55	2.58	2.55	2.58
2.23	2.59	2.23	2.61	2.55	2.54	2.55	2.48
2.16	2.48	2.16	2.52	2.52	2.55	2.52	2.49
2.12	2.39	2.12	2.39	2.97	2.87	2.97	2.88
2.79	2.87	2.79	2.93	3.76	3.97	3.76	4.04
3.99	4.31	3.99	4.33	3.57	3.91	3.57	3.93
3.37	3.8	3.37	3.74	3.35	3.19	3.35	3.18
2.69	2.79	2.69	2.88	3.3	3.4	3.3	3.41
2.42	2.35	2.42	2.68	2.85	2.83	2.85	2.76
2.36	2.44	2.36	2.45	4.43	4.44	4.43	4.43
5.11	5.22	5.11	5.09	4.17	4.34	4.17	4.36
RMSD	0.24	RMSD	0.25	RMSD	0.15	RMSD	0.15

Table S12. Conformational populations derived by NAMFIS-analysis of PROTAC **1** in $CDCl_3$ solution.

**SS Isomer**		**SR Isomer**	
Conf. No.	Popu. (%)	Conf. No.	Popu. (%)
1	13	1	38
2	9	2	3
3	23	3	36
4	18	4	3
5	36	5	17

Table S13. Conformational populations derived by NAMFIS-analysis of PROTAC **2** in $CDCl_3$ solution.

**SS Isomer**		**SR Isomer**	
Conf. No.	Popu. (%)	Conf. No.	Popu. (%)
1	2	1	45
2	4	2	7
3	10	3	6
4	31	4	32
5	4	5	4
6	7	6	2
7	37		

SS Conformations

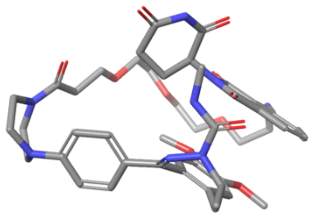

Conf. 1
Population 13%

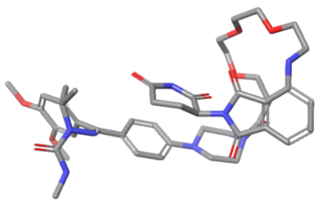

Conf. 2
Population 9%

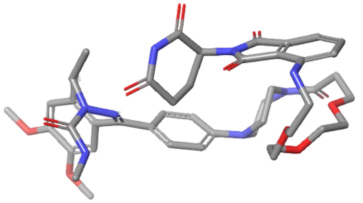

Conf. 3
Population 23%

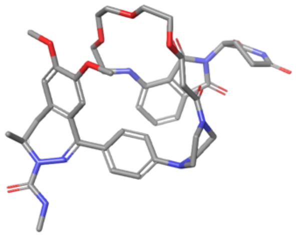

Conf. 4
Population 18%

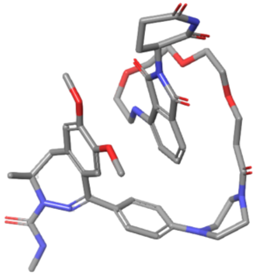

Conf. 5
Population 36%

SR Conformations

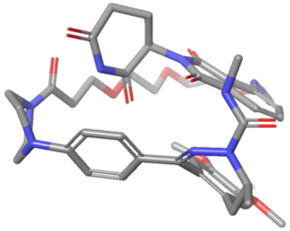

Conf. 1
Population 38%

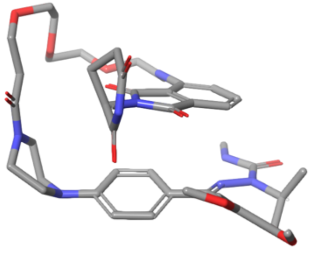

Conf. 2
Population 3%

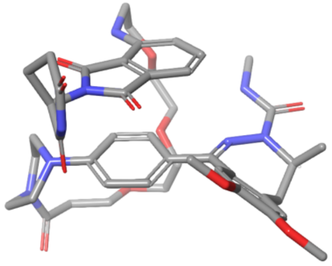

Conf. 3
Population 36%

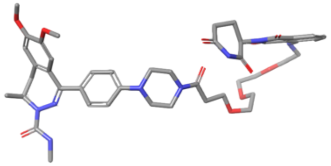

Conf. 4
Population 3%

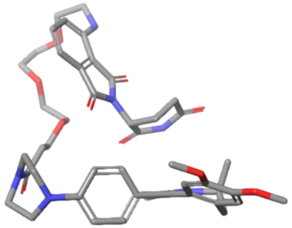

Conf. 5
Population 17%

Figure S9. Structures of the conformations in the ensembles adopted by PROTAC **1** in $CDCl_3$

SS Conformations

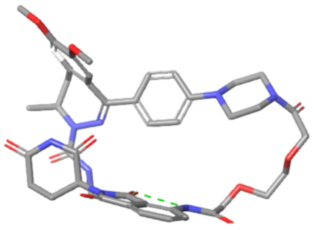

Conf. 1
Population 2%

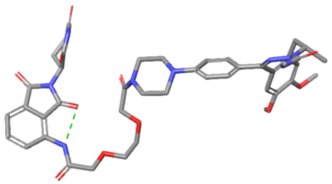

Conf. 2
Population 4%

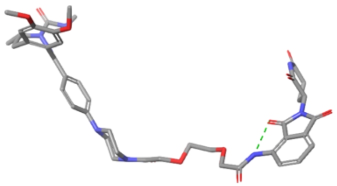

Conf. 3
Population 10%

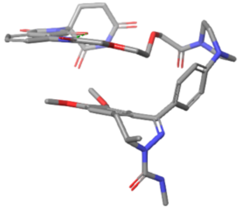

Conf. 4
Population 31%

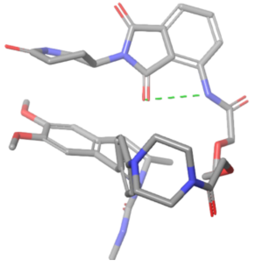

Conf. 5
Population 17%

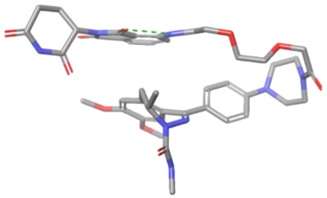

Conf. 6
Population 7%

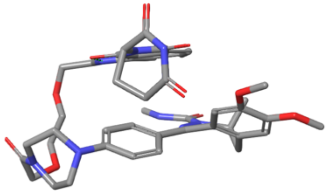

Conf. 7
Population 37%

SR Conformations

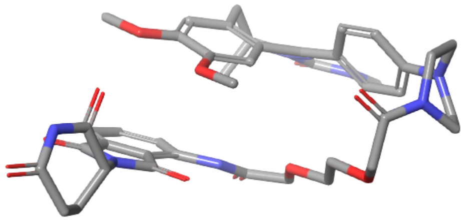

Conf. 1
Population 45%

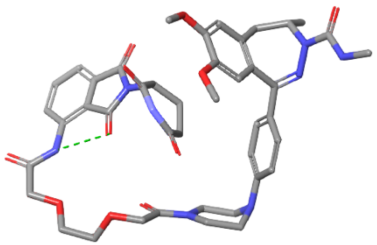

Conf. 2
Population 7%

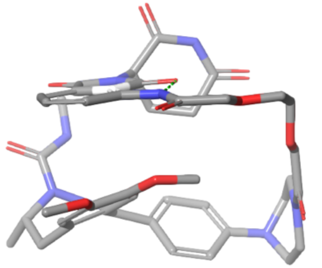

Conf. 3
Population 6%

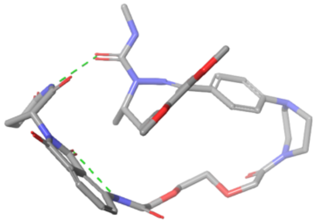

Conf. 4
Population 32%

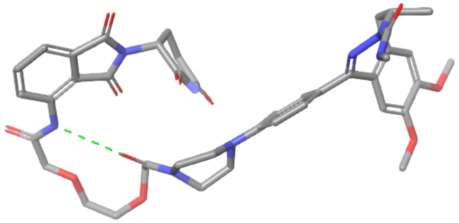

Conf. 5
Population 4%

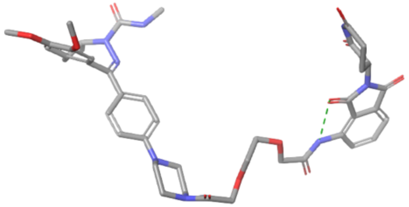

Conf. 6
Population 2%

Figure S10. Structures of the conformations in the ensembles adopted by PROTAC **2** in $CDCl_3$

Noncovalent interaction analysis (NCI) plots were generated for the solution ensembles of PROTACs **1** and **2** as reported.[4] NCI isosurfaces were obtained from a cube of electron density values as implemented in the open source program Jmol.[5] The isosurfaces in Figures S11 and S12 were accessed using Rzepa's web implementation.[6]

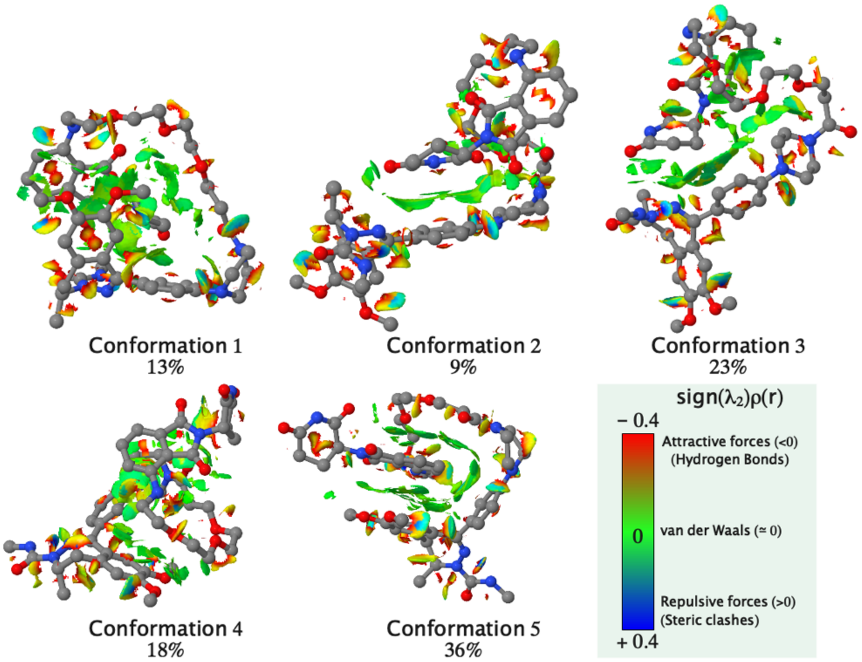

Figure S11. NCI plot analysis of PROTAC S,S-**1**.

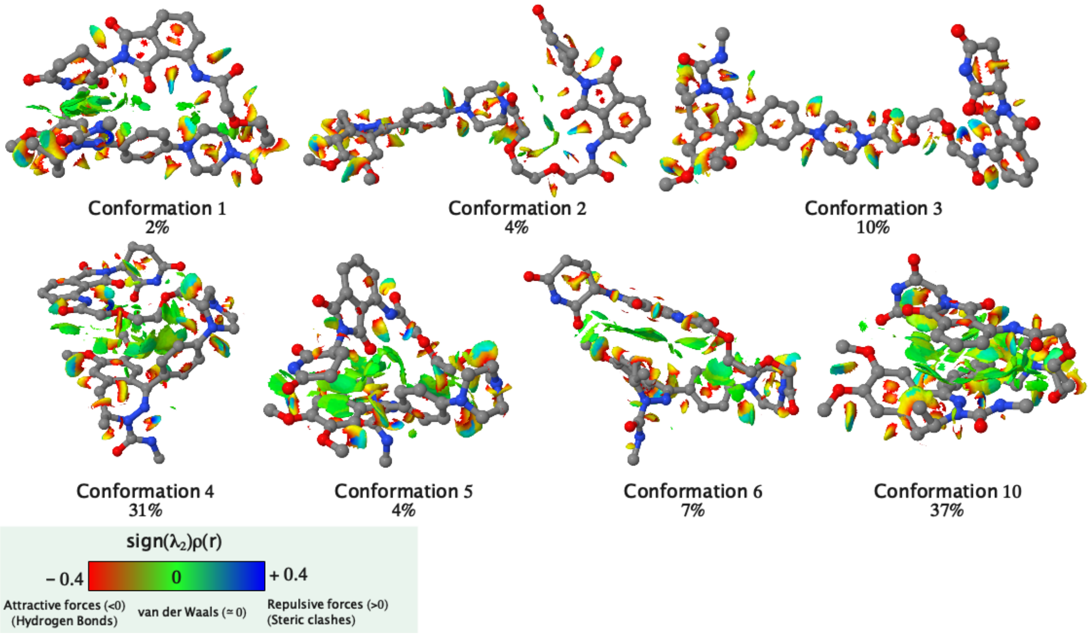

Figure S12. NCI plot analysis of PROTAC S,S-**2**.

3.5. Akaike information criterion (AIC) analyses

In addition to the NAMFIS analysis the two isomers were compared by performing Akaike information criterion (AIC) analyses.[7] AIC is a statistical method which is used to compare how well different models define the reality. The summary of AIC results for the isomers of PROTACs **1** and **2** are given below (Table **S14**).

Table S14. Akaike information criterion (AIC) analyses conformations[a]

		SSD (ERRLimit)	AIC (ERRLimit)	AICc (ERRLimit)	**ΔAICc**	**Wi**	**Eri**	**LERi**
Compound **1** $CDCl_3$	**SS** isomer	21.1	31.1	31.8	0.0	0.79	1.00	0.00
	**SR** isomer	23.3	33.3	33.9	2.1	0.26	2.86	0.46
Compound **2** $CDCl_3$	**SS** isomer	3.0	17	17.9	1.7	0.30	2.34	0.37
	**SR** isomer	3.4	15.4	16.2	0.0	0.70	1.00	0.00

[a]SSD is the sum of square differences between the measured and modeled distances[7]

AIC is the Akaike information criterion, AICc is the small sample size corrected AIC that has a compensation for overfitting for systems with small sample size, ΔAICc is the difference in AIC of an individual model as compared to the best model where w_{best} is the weight of the best model, w_i the weight of other individual models, and LER_i the Logarithmic Evidence Ratio of a model.[7]

The best model has the lowest AIC value, the ER_i of 1 and a LER_i value of 0. The LER_i difference is interpreted as 'weak' when LER_i 0−0.5 , as 'substantial' 0.5−1, as 'strong' 1−2 and as 'decisive' >2.

3.6. Structural comparison of conformers in solution ensembles

Table S15. RMSD matrix for all conformations

SS Conformations of PROTAC **1** in $CDCl_3$

Conf no.	1	2	3	4
1	0			
2	5.944	0		
3	5.214	3.601	0	
4	4.154	6.092	5.609	0
5	5.288	5.311	5.823	4.204

SR Conformations of PROTAC **1** in $CDCl_3$

Conf. no	1	2	3	4
1	0			
2	4.302	0		
3	4.537	3.23	0	
4	6.601	6.271	6.578	0
5	5.236	3.919	4.243	5.734

SS Conformations of PROTAC **2** in $CDCl_3$

Conf. no.	1	2	3	4	5	6
1	0					
2	6.785	0				
3	7.012	3.678	0			
4	5.39	5.715	5.701	0		
5	5.3	6.593	6.979	4.97	0	
6	3.568	7.365	7.462	4.735	4.817	0
7	3.579	6.155	7.036	4.299	4.621	4.368

SR Conformations of PROTAC **2** in $CDCl_3$

Conf. no.	1	2	3	4	5
1	0				
2	4.575	0			
3	4.499	4.932	0		
4	4.594	3.65	4.436	0	
5	6.467	4.954	6.33	4.735	0
6	6.563	6.352	6.366	6.361	4.059

Table S16. Overview of solution conformations and calculated descriptors for each conformation of PROTACs **1–2** in the ensembles obtained by NAMFIS analysis.

PROTACs	Conf. No	Stereoisomers	Popul. (%)	SA 3D PS (Å^2)[a]	R_{gyr} (Å)[b]
**1**	1	S,S	13	187.3	5.0
	2	S,S	9	151.8	5.8
	3	S,S	23	235.8	5.7
	4	S,S	18	210.3	5.4
	5	S,S	36	213.3	5.3
Population weighted mean				***209***	***5.40***
**1**	1	S,R	38	123.2	4.9
	2	S,R	3	210.3	5.1
	3	S,R	36	198.6	5
	4	S,R	3	211	7.8
	5	S,R	17	183.1	5.5
Population weighted mean				***167***	***5.16***
**2**	1	S,S	2	205.4	5.5
	2	S,S	4	261.6	7.5
	3	S,S	10	300.5	8.5
	4	S,S	31	247.0	5.3
	5	S,S	4	235.6	5.0
	6	S,S	7	262.4	5.5
	7	S,S	37	228.5	4.9
Population weighted mean				***232.26***	***5.44***
**2**	1	S,R	45	243	5.4
	2	S,R	7	225.7	4.8
	3	S,R	6	230.4	5.4
	4	S,R	32	218	5.4
	5	S,R	4	205.6	6.4
	6	S,R	2	302.3	7.6
Population weighted mean				***245.84***	***5.60***

[a]SA 3D PSA = solvent accessible 3D polar surface area. [b]R_{gyr} = radius of gyration.

4. Variable temperature NMR studies

The variable temperature NMR studies for PROTACs **1-3** were conducted by recording ^{1}H NMR spectra at different temperatures on a 500 MHz BRUKER Avance III HD NMR spectrometer equipped with a Z150347_0001 (CP TXO 500S2 C/ N-H-D-05 Z) probe. ^{1}H spectra were recorded using two different solvents (DMSO-d_6 and $CDCl_3$) with a relaxation delay of 0.7 s, 16 scans and 32768 points direct dimension.

The amide temperature coefficients ($\Delta\delta_{NH}/\Delta T$, ppb K^{-1}) were obtained from ($\delta_{T,high}$-$\delta_{T,low}$)/(T_{high}-T_{low}). A value of $\Delta\delta_{NH}/\Delta T$; < 3 indicates a strong intramolecular hydrogen bond, between 3–5 indicates that the amide proton is in equilibrium between a solvent exposed and an intramolecular hydrogen bond and >5 indicates the amide proton is solvent exposed. The results are summarized in Table S17-S19.[8]

Table S17: Amide temperature coefficients, $\Delta\delta_{NH}/\Delta T$ (ppb K^{-1}) for PROTAC **1** in **DMSO-*d*$_6$** and **$CDCl_3$**

DMSO-*d*$_6$				$CDCl_3$			
°T	3-NH δ ppm	46-NH δ ppm	61-NH δ ppm	°T	3-NH δ ppm	46-NH δ ppm	61-NH δ ppm
25	6.59	6.31	11.08	10	4.49	6.176	8.95
35	6.58	6.29	11.04	20	4.491	6.202	8.85
45	6.56	6.27	10.99	30	4.492	6.206	8.72
55	6.55	6.26	10.94	40	4.493	6.207	8.61
65	6.54	6.24	10.9	50	4.493	6.207	8.51
$\Delta\delta_{NH}$	-0.05	-0.07	-0.18	$\Delta\delta_{NH}$	0.003	0.031	-0.44
**$\Delta\delta_{NH}/\Delta T$**	**-1.3**	**-1.7**	**-4.5**	**$\Delta\delta_{NH}/\Delta T$**	**0.1**	**0.8**	**-11.0**

Table S18: Amide temperature coefficients, $\Delta\delta_{NH}/\Delta T$ (ppb K^{-1}) for PROTAC **2** in **DMSO-*d*$_6$** and **$CDCl_3$**

DMSO-*d*$_6$				CDCl$_3$			
°T	20-NH δ ppm	54-NH δ ppm	16-NH δ ppm	°T	20-NH δ ppm	54-NH δ ppm	16-NH δ ppm
25	10.35	6.31	11.15	10	10.46	6.246	8.84
35	10.34	6.29	11.1	20	10.455	6.258	8.83
45	10.33	6.28	11.06	30	10.45	6.261	8.74
55	10.32	6.26	11.02	40	10.44	6.2558	8.64
65	10.31	6.24	10.94	50	10.43	6.248	8.54
$\Delta\delta_{NH}$	-0.04	-0.07	-0.21	$\Delta\delta_{NH}$	-0.03	0.002	-0.3
**$\Delta\delta_{NH}/\Delta T$**	**-1.0**	**-1.7**	**-5.3**	**$\Delta\delta_{NH}/\Delta T$**	**-0.8**	**0.0**	**-7.5**

Table S19: Amide temperature coefficients, $\Delta\delta_{NH}/\Delta T$ (ppb K^{-1}) for PROTAC **3** in **DMSO-*d*$_6$** and **$CDCl_3$**

DMSO-*d*$_6$				CDCl$_3$			
°T	6-NH δ ppm	42-NH δ ppm	57-NH δ ppm	°T	6-NH δ ppm	42-NH δ ppm	57-NH δ ppm
25	7.97	6.33	11.11	10	7.72	6.283	9.12
35	7.93	6.31	11.06	20	7.69	6.274	8.98
45	7.89	6.29	11.02	30	7.66	6.272	8.88
55	7.85	6.27	10.97	40	7.64	6.268	8.78
65	7.82	6.25	10.93	50	7.61	6.257	8.68
$\Delta\delta_{NH}$	-0.15	-0.08	-0.18	$\Delta\delta_{NH}$	-0.11	-0.026	-0.44
**$\Delta\delta_{NH}/\Delta T$**	**-3.7**	**-2.0**	**-4.5**	**$\Delta\delta_{NH}/\Delta T$**	**-2.7**	**-0.7**	**-11.0**

5. NMR Spectra of PROTACs 1-3

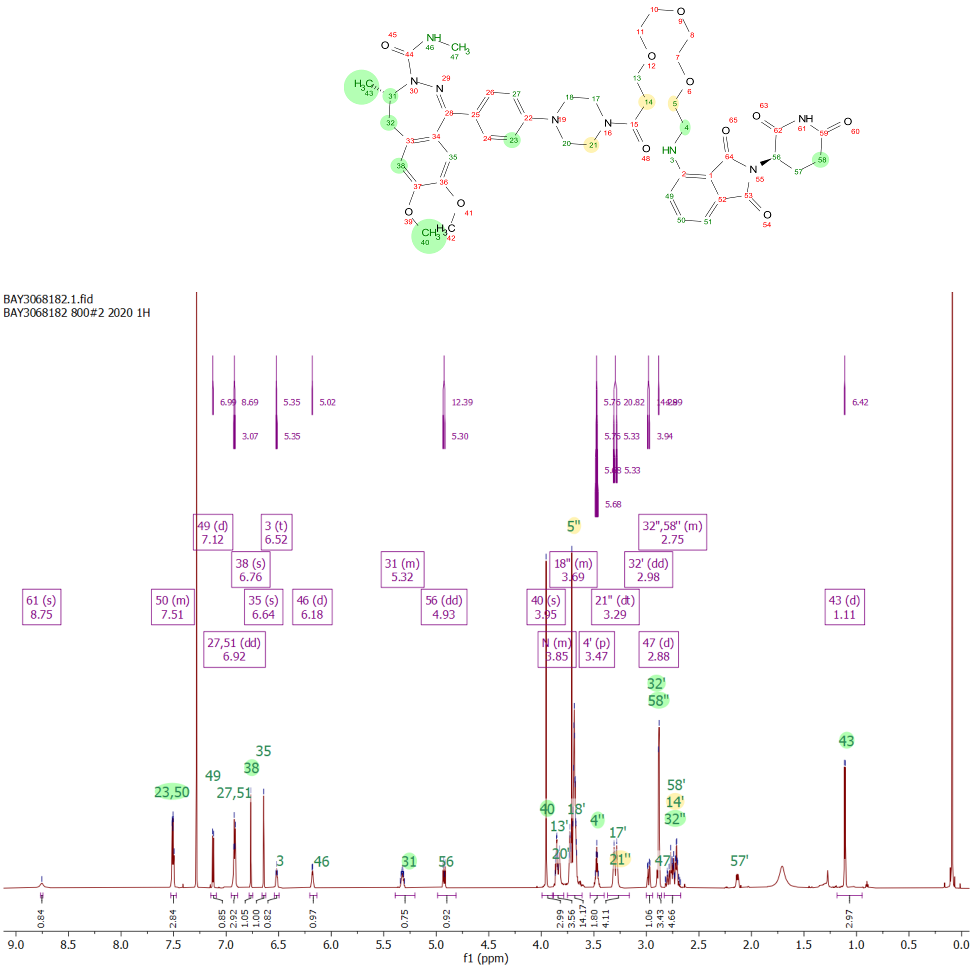

Figure S13. ^{1}H NMR Spectrum of PROTAC **1** ($CDCl_3$)

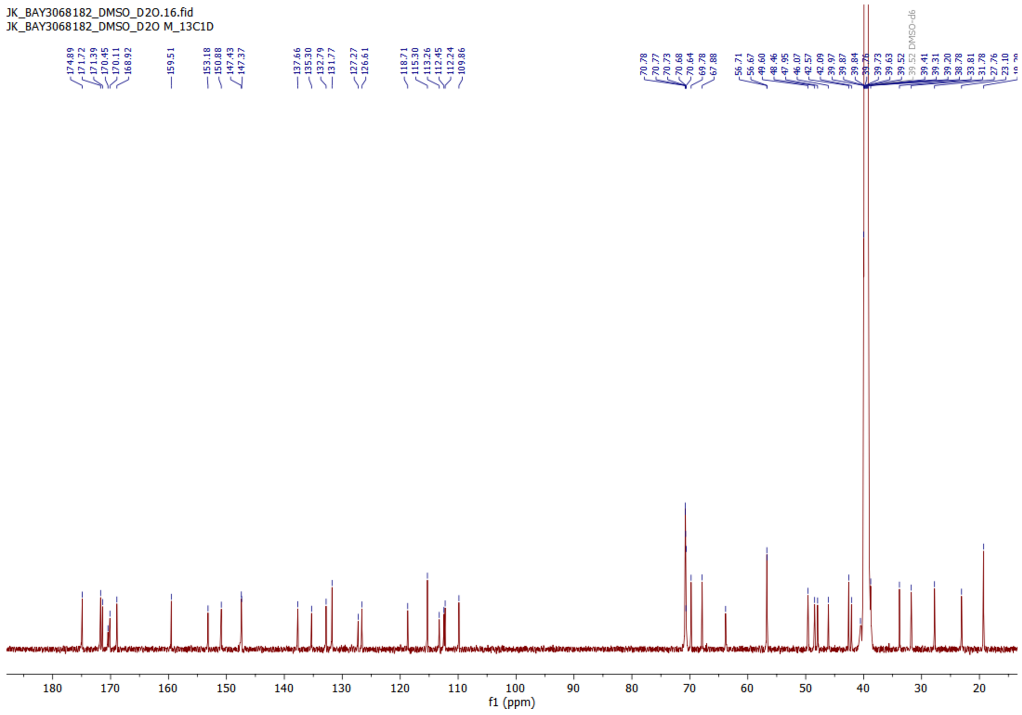

Figure S14. ^{13}C Spectrum of PROTAC **1** (DMSO-d_6:D_2O 2:1)

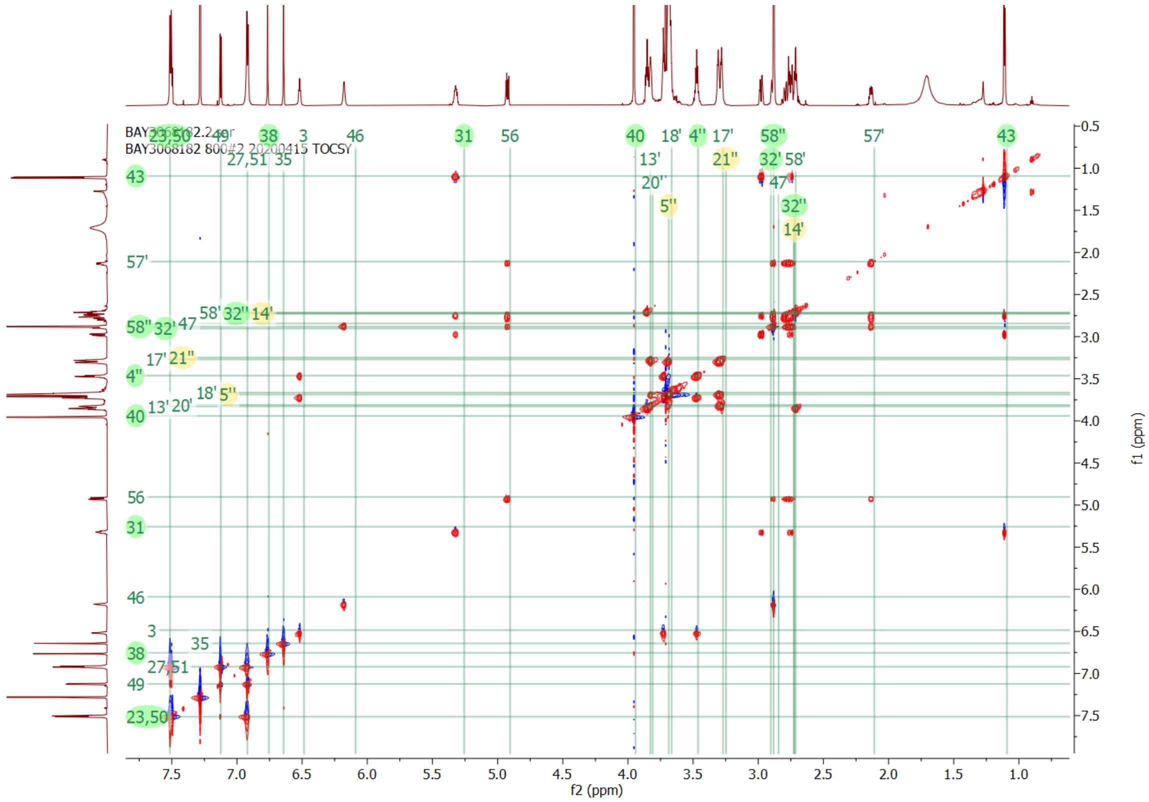

Figure S15. TOCSY Spectrum of PROTAC **1** ($CDCl_3$)

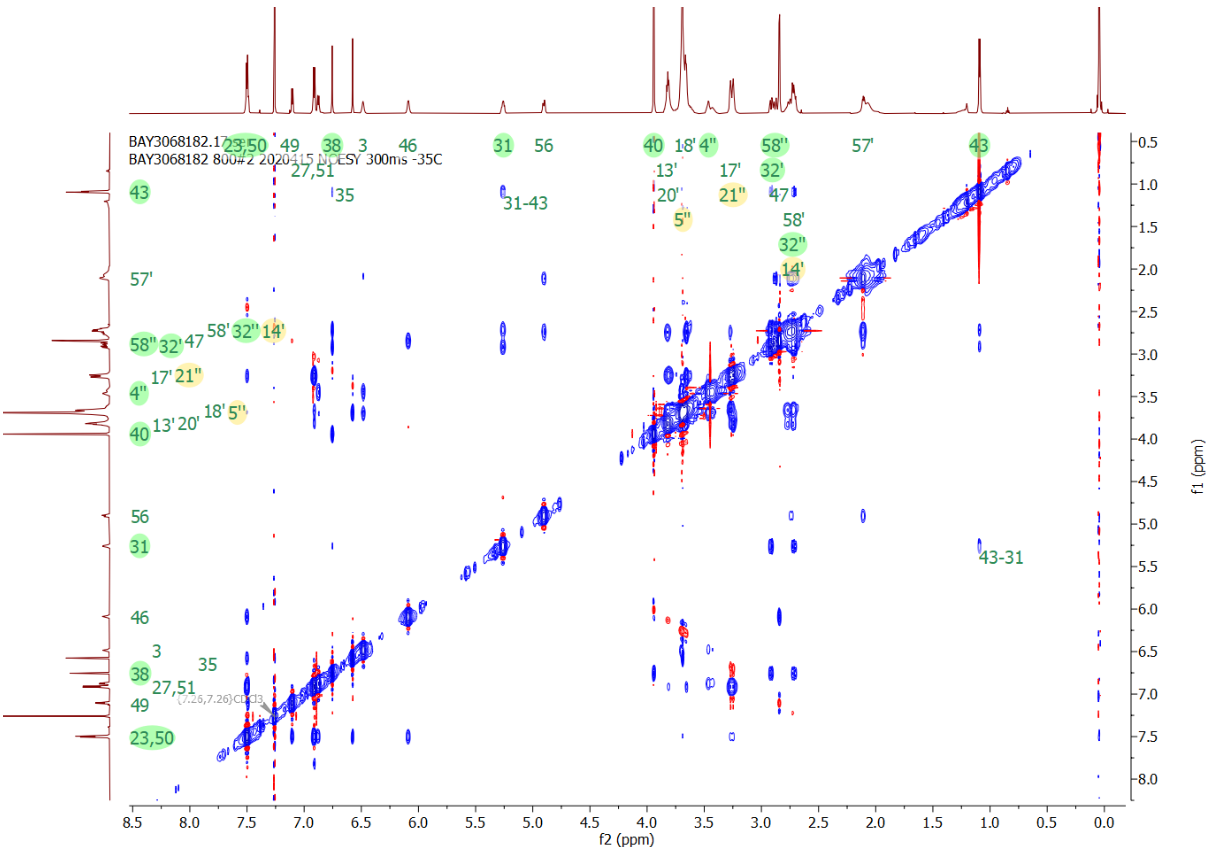

Figure S16. NOESY Spectrum of PROTAC **1** ($CDCl_3$)

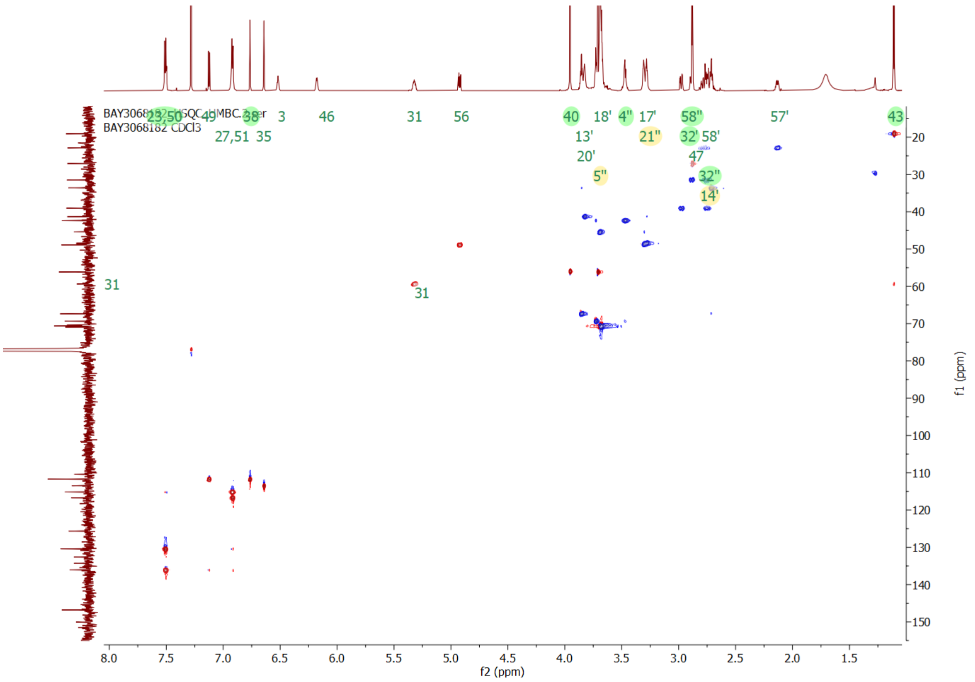

Figure S17. HSQC Spectrum of PROTAC **1** ($CDCl_3$)

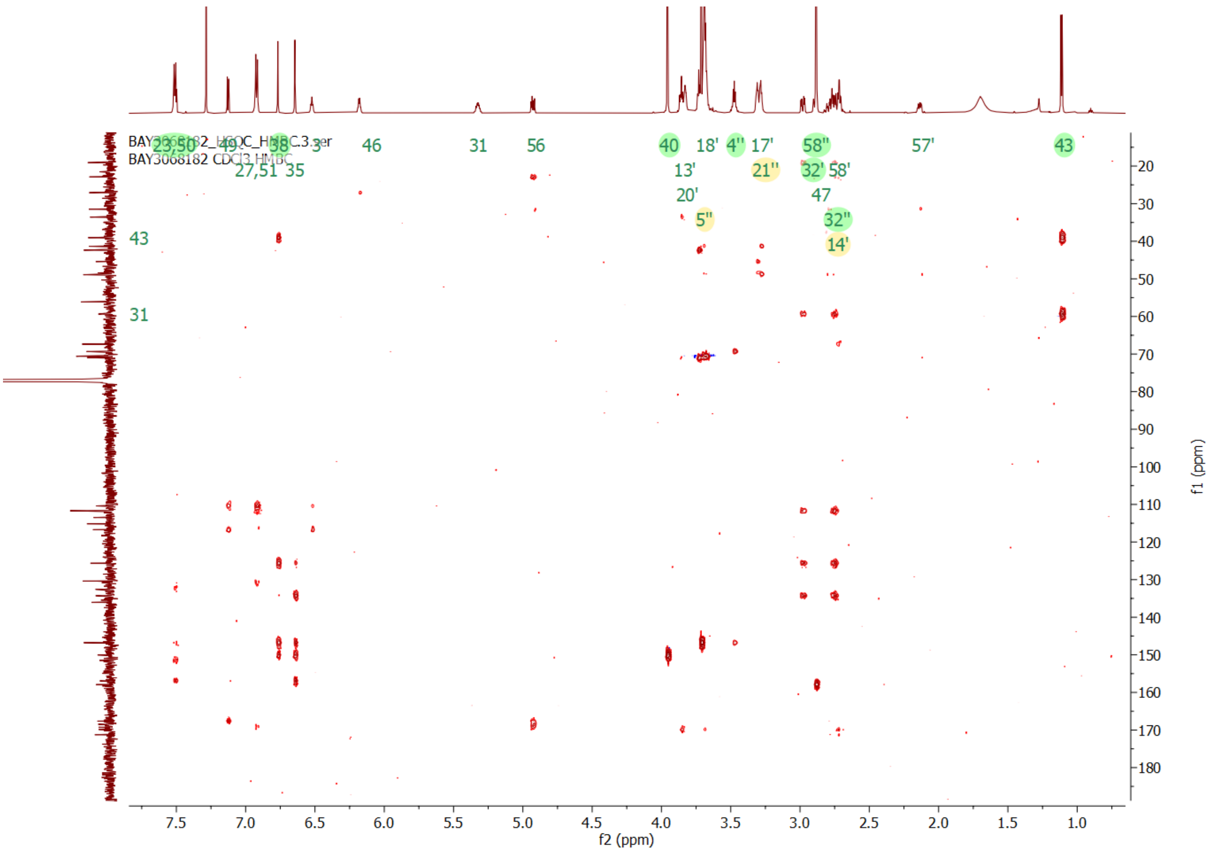

Figure S18. HMBC Spectrum of PROTAC **1** ($CDCl_3$)

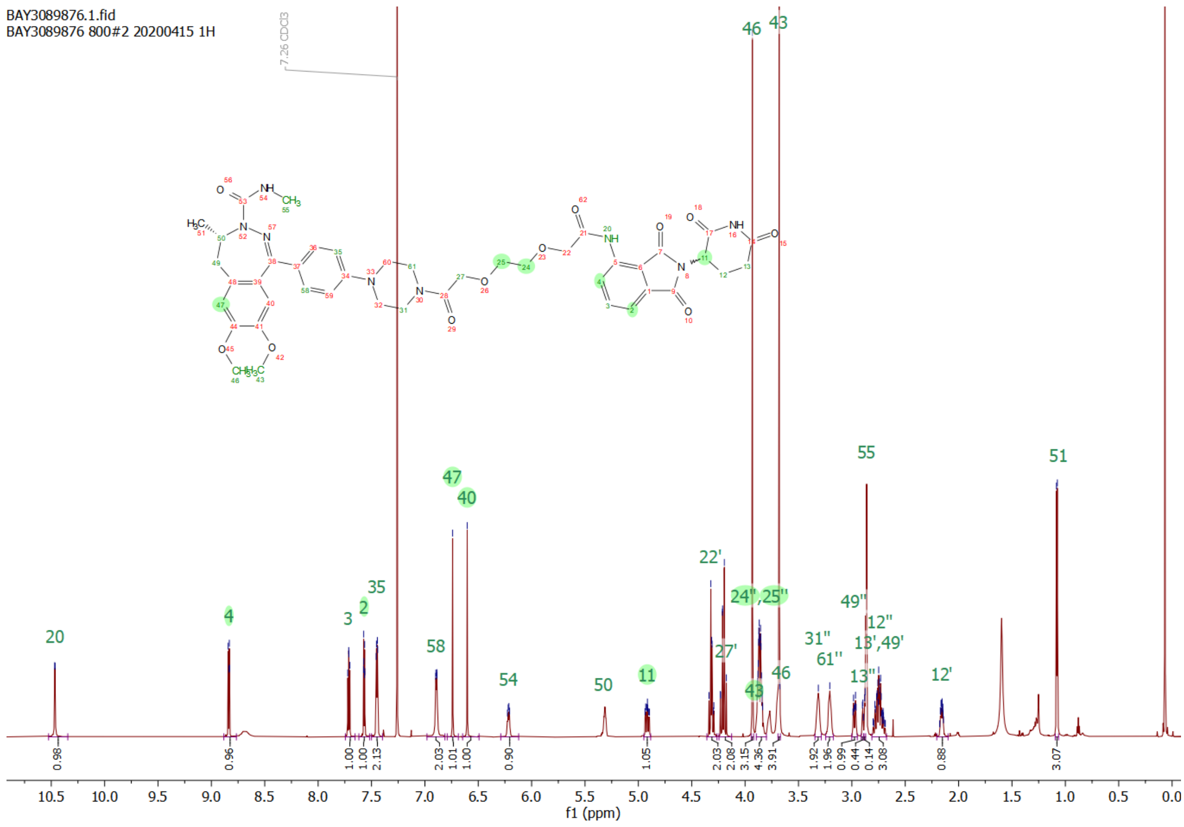

Figure S19. [1]H Spectrum of PROTAC **2** ($CDCl_3$)

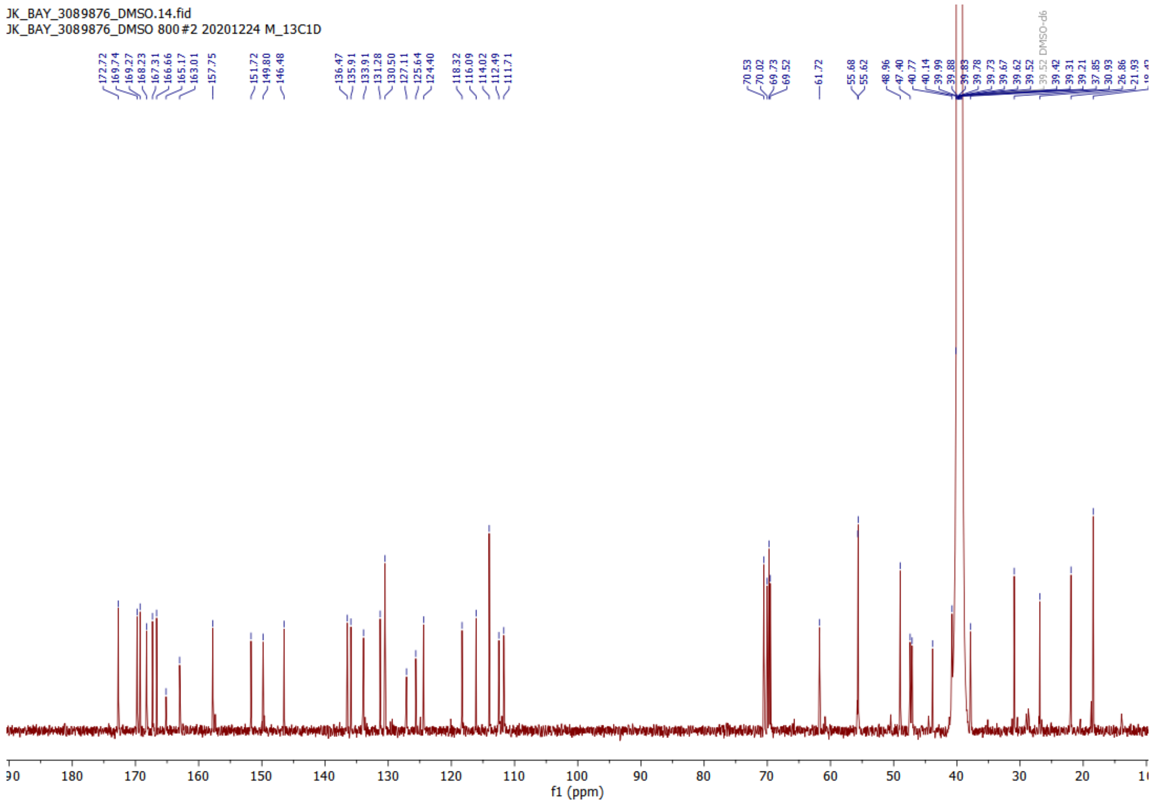

Figure S20. [13]C Spectrum of PROTAC **2** (DMSO-d_6)

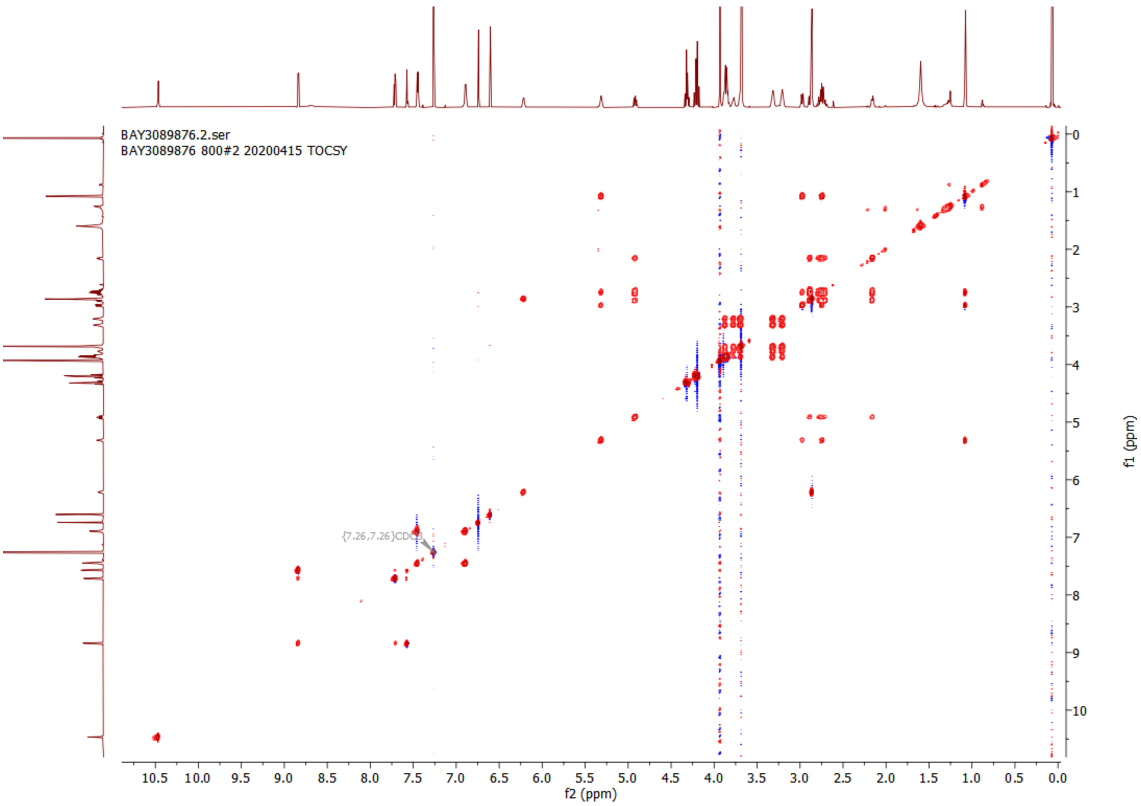

Figure S21. TOCSY Spectrum of PROTAC **2** ($CDCl_3$)

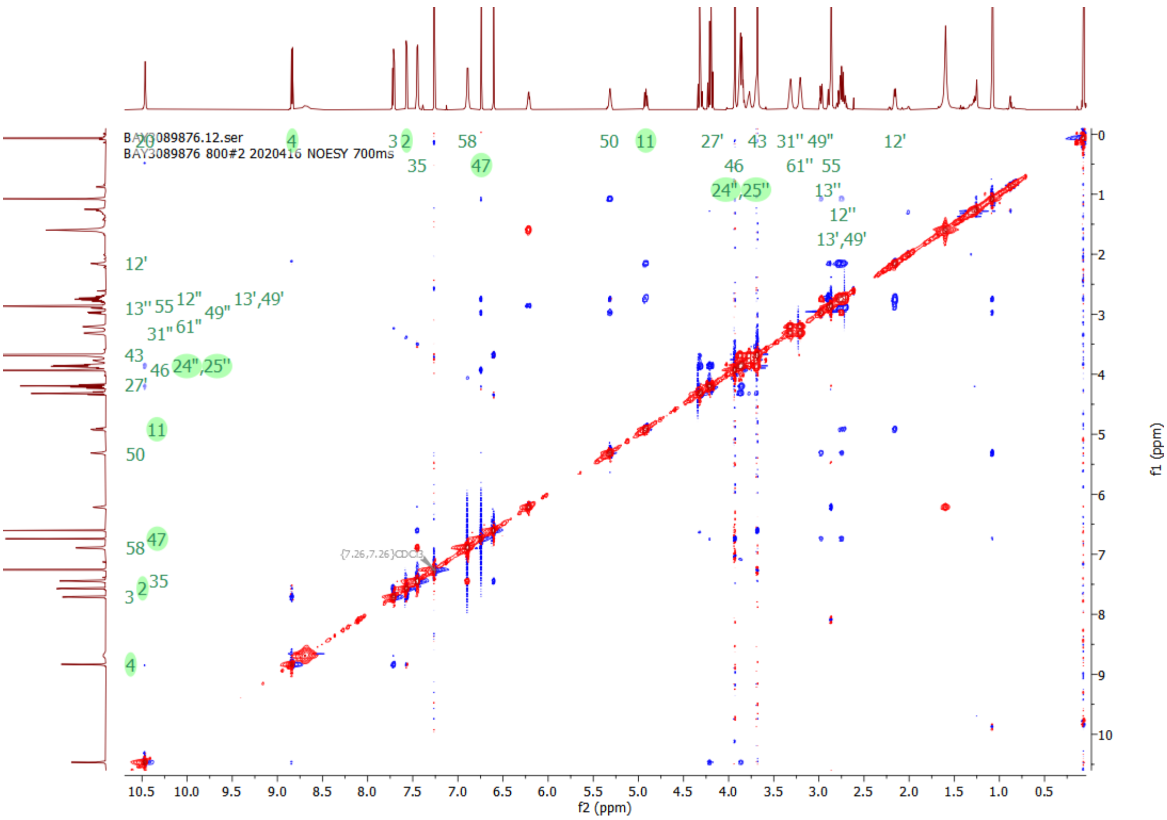

Figure S22. NOESY Spectrum of PROTAC **2** ($CDCl_3$)

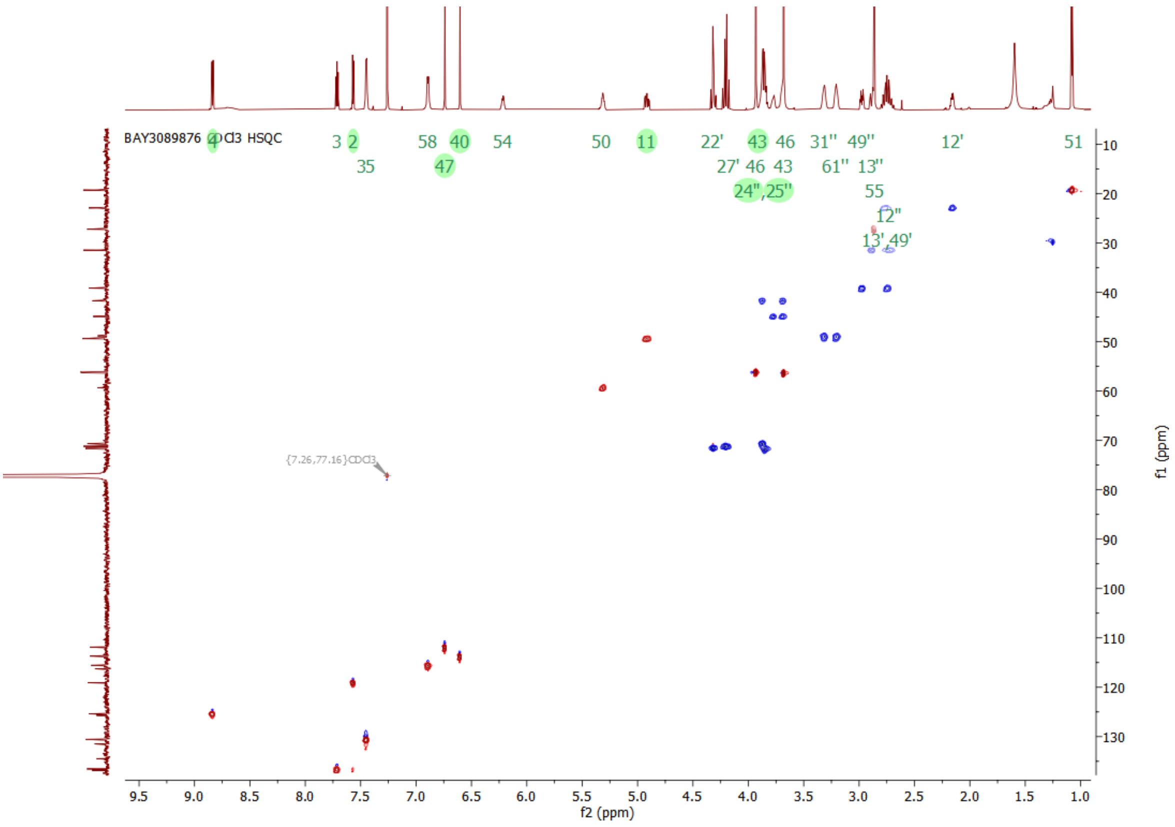

Figure S23. HSQC Spectrum of PROTAC **2** ($CDCl_3$)

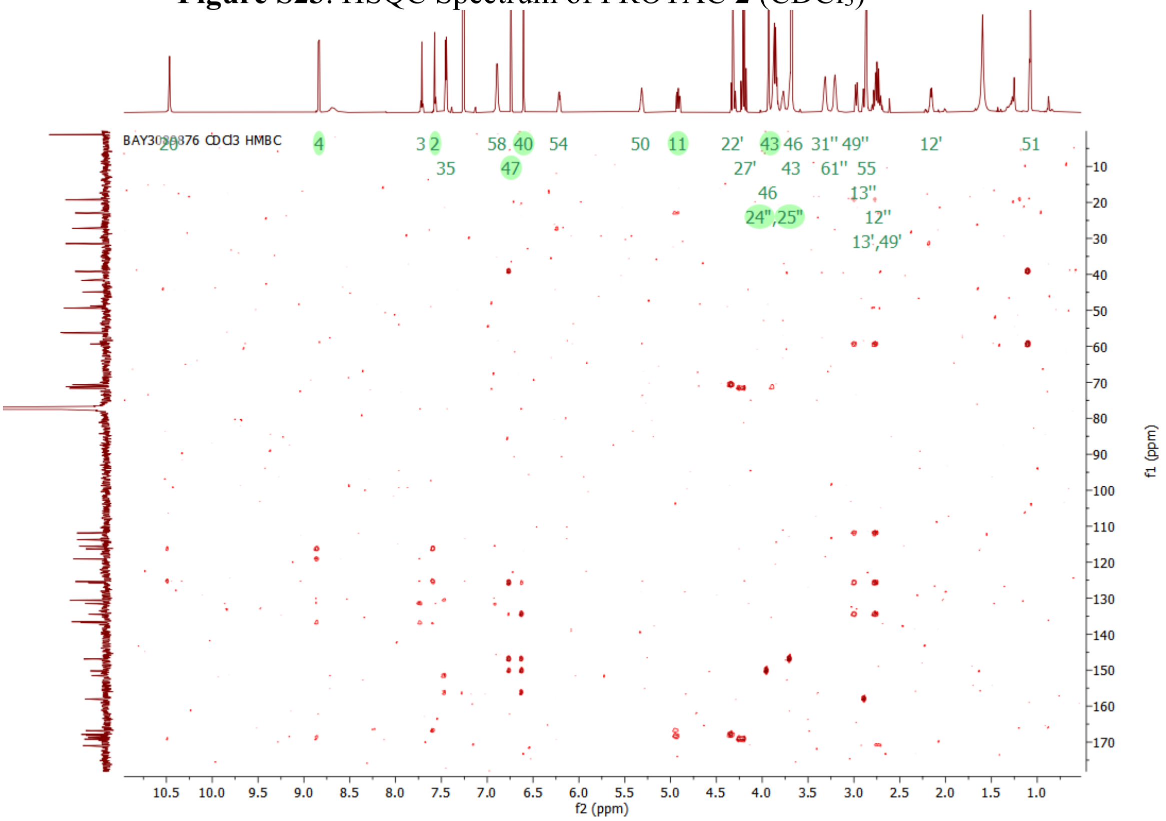

Figure S24. HMBC Spectrum of PROTAC **2** ($CDCl_3$)

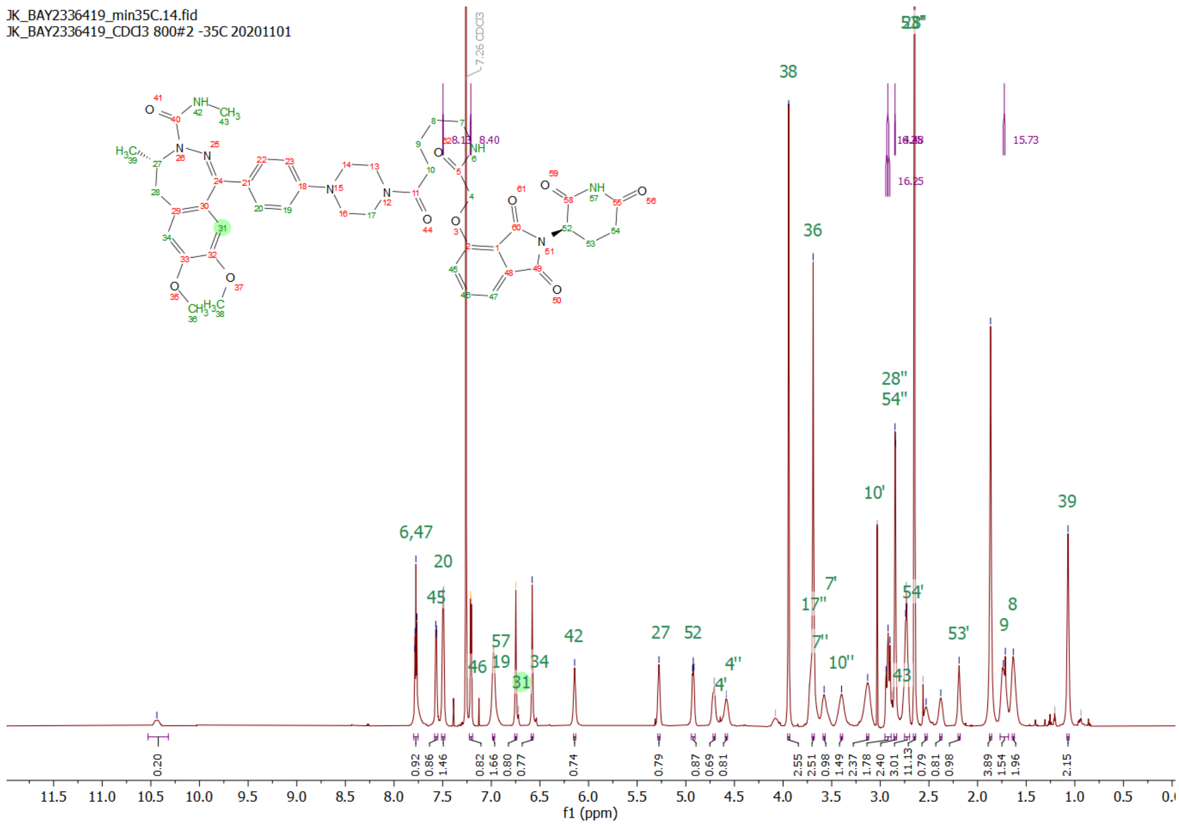

Figure S25. [1]H Spectrum of PROTAC **3** ($CDCl_3$)

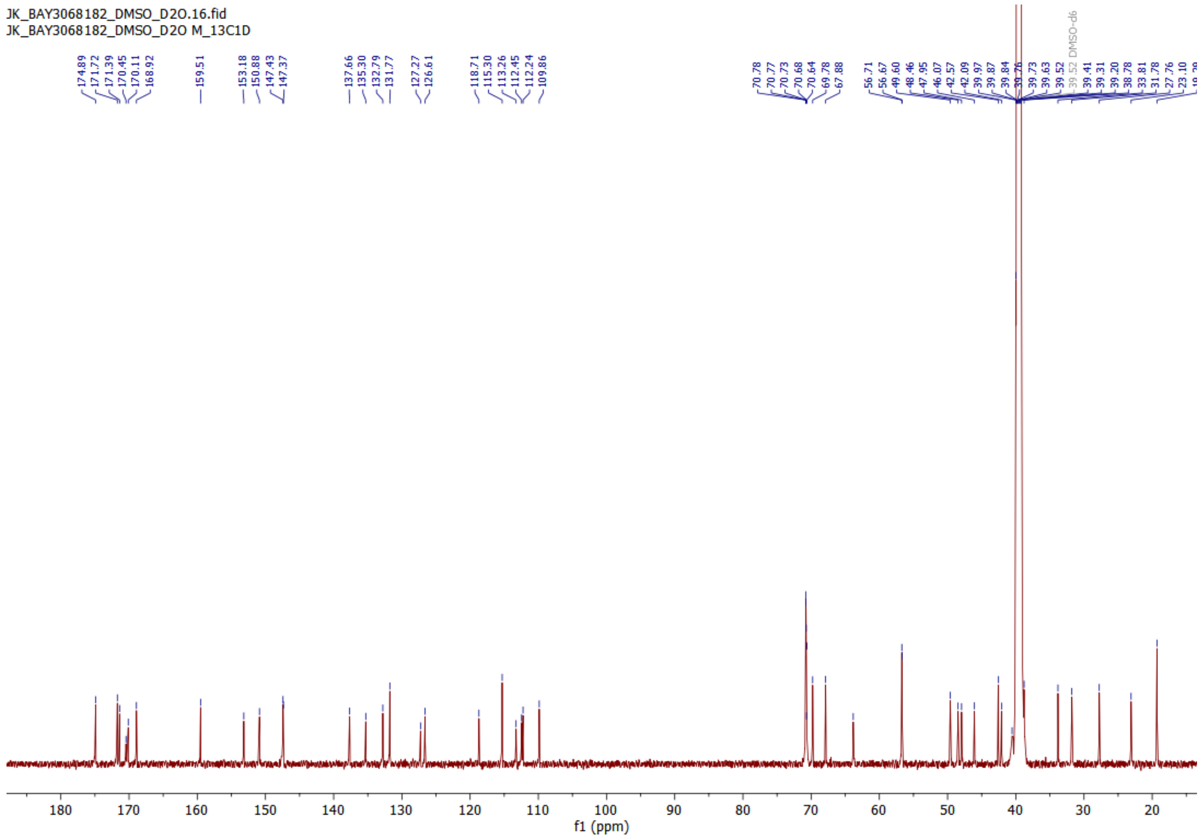

Figure S26. [13]C Spectrum of PROTAC **3** (DMSO-d_6:D_2O 2:1)

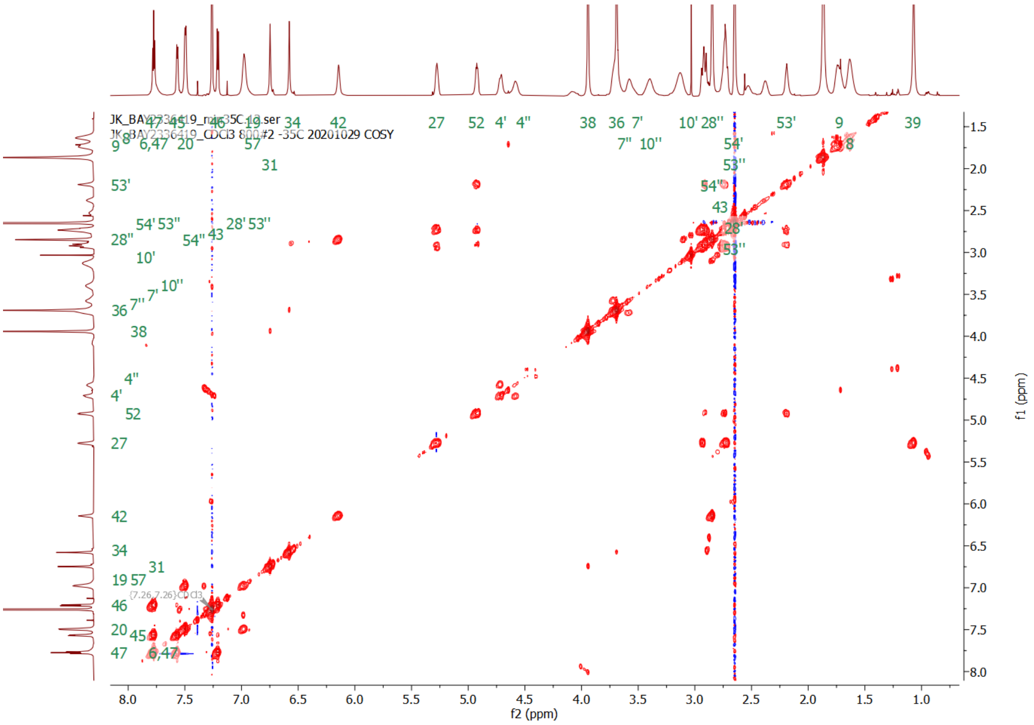

Figure S27. COSY Spectrum of PROTAC **3** ($CDCl_3$)

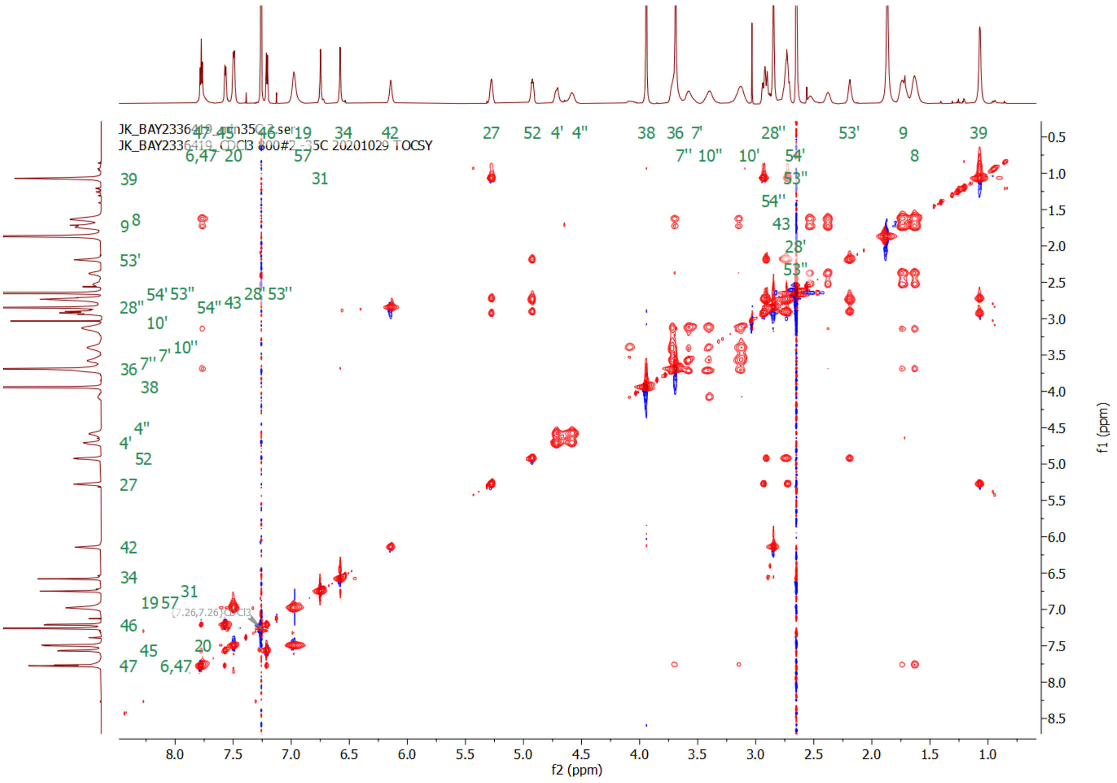

Figure S28. TOCSY Spectrum of PROTAC **3** ($CDCl_3$)

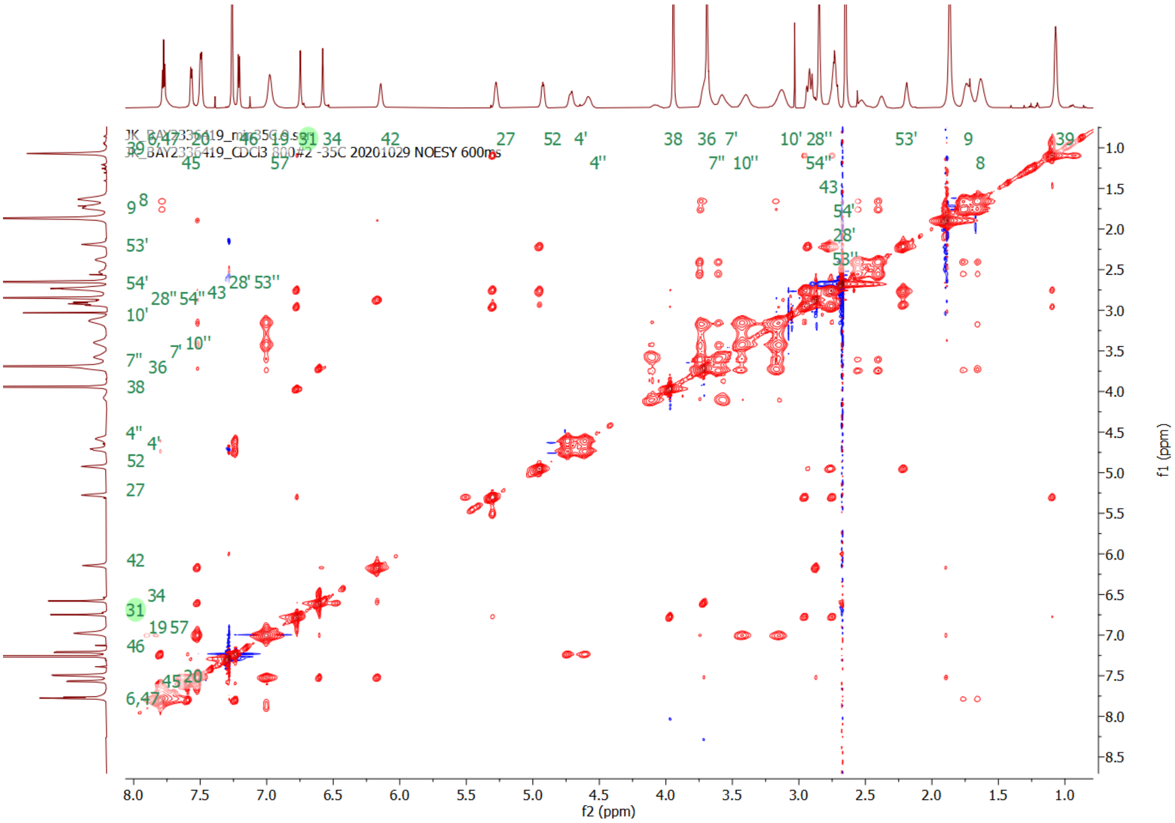

Figure S29. NOESY Spectrum of PROTAC **3** ($CDCl_3$)

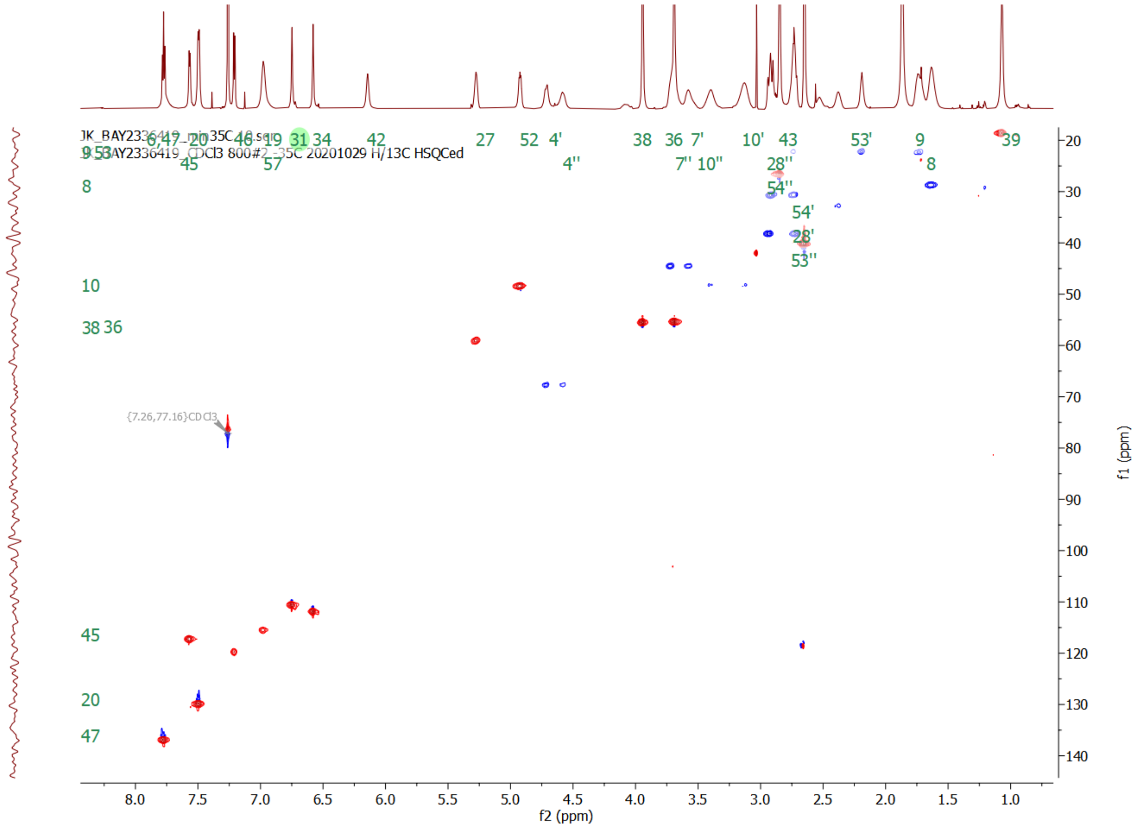

Figure S30. HSQC Spectrum of PROTAC **3** ($CDCl_3$)

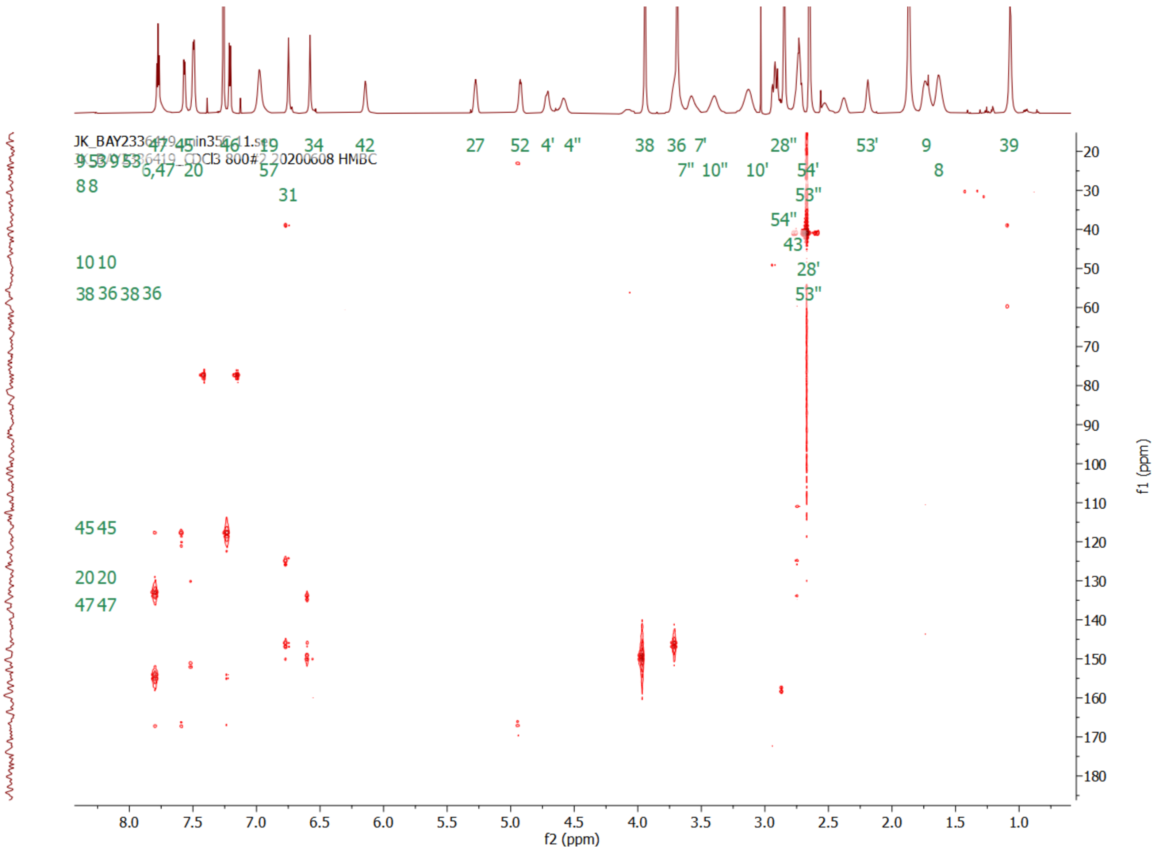

Figure S31. HMBC Spectrum of PROTAC **3** ($CDCl_3$)

6. Computational methods and analyses of MD simulations of 1–3

6.1. Trajectory analysis of conformational space for PROTACs 1-3.

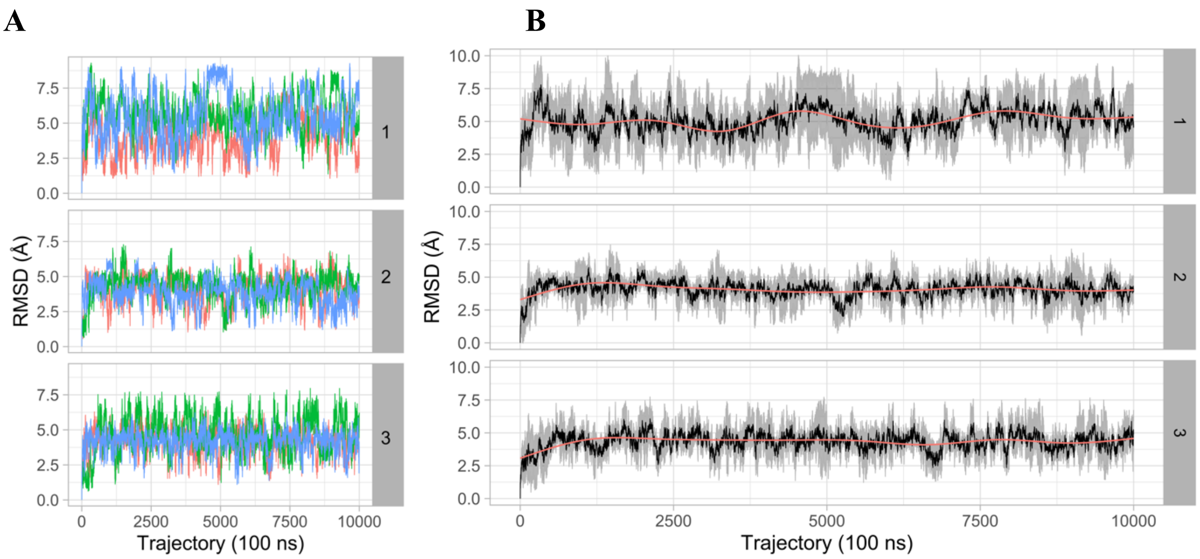

Figure S32. RMSD time series for the MD simulations of PROTACs relative to the initial position. **A)** RMSD series for each of the three independent replicates of PROTACs **1**–**3** [red (replicate 1), green (replicate 2), and blue (replicate 3)]. **B)** Averaged RMSD (black lines) over the three replicates, gray shading represents standard deviations.

6.2. Conformation-dependent molecular property space

Table S20. Summary of intramolecular hydrogen bonding in the 100 ns simulations for PROTACs **1-3.** Averaged over three independent replicates.

1

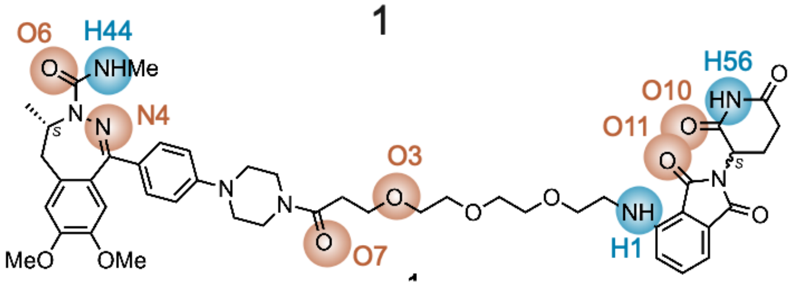

Acceptor	DonorH	Donor	Fraction of HB (%)
O7	H56	N8	35.9
O11	H1	N1	18.2
O6	H56	N8	3.7
O3	H56	N8	0.38
O7	H1	N1	0.06
O10	H44	N6	0.04
N4	H56	N8	0.03

2

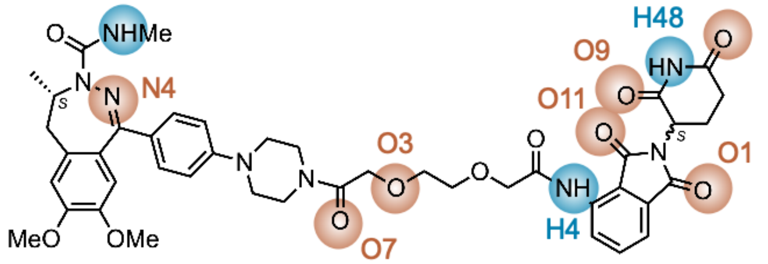

Acceptor	DonorH	Donor	Fraction of HB (%)
O7	H48	N8	28.3
O11	H4	N1	8.65
O7	H4	N1	1.49
N4	H48	N8	0.74
O1	H25	N6	0.75
O10	H25	N6	0.70
O9	H25	N6	0.19
O3	H4	N1	0.12

3

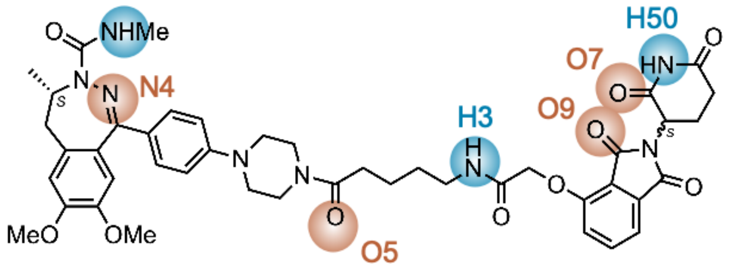

Acceptor	DonorH	Donor	Fraction of HB (%)
O9	H3	N1	11.7
O7	H38	N6	0.6
N4	H50	N8	0.5
O5	H3	N1	0.3

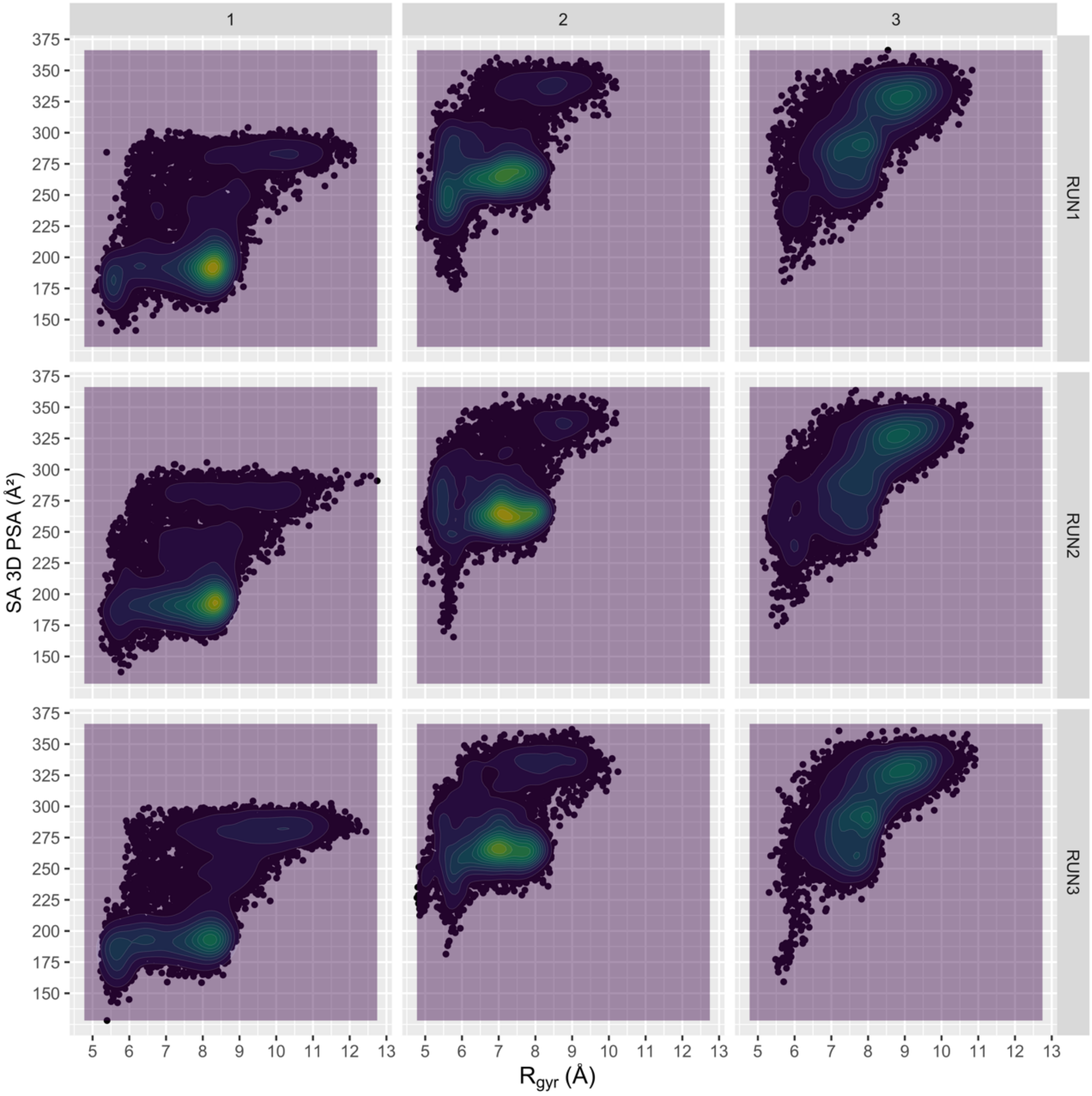

Figure S33. Density properties [SA 3D PSA (Å^2) versus R_{gyr} (Å)] for PROTACs **1**–**3**. Data has been plotted for the three independent replicates of the 100 ns simulations.

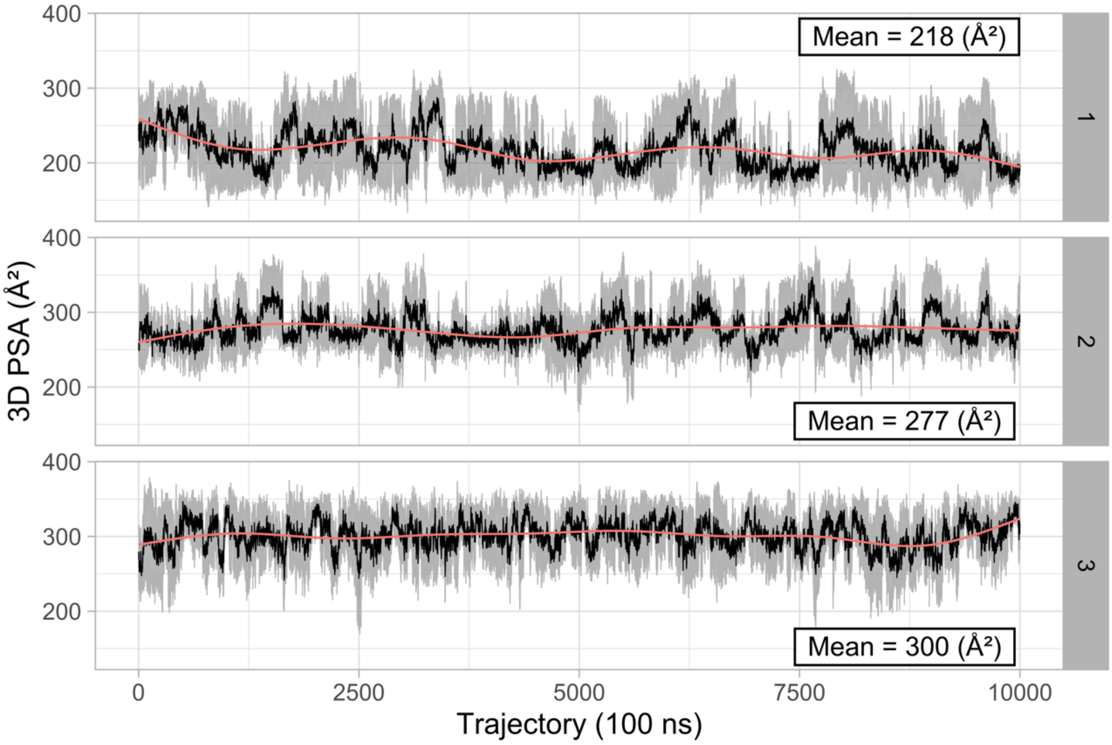

Figure S34. Solvent accessible 3D polar surface area (SA 3D PSA) time series for PROTACs **1**–**3**. Averaged (black lines) over the three independent replicates, gray shading represents standard deviations.

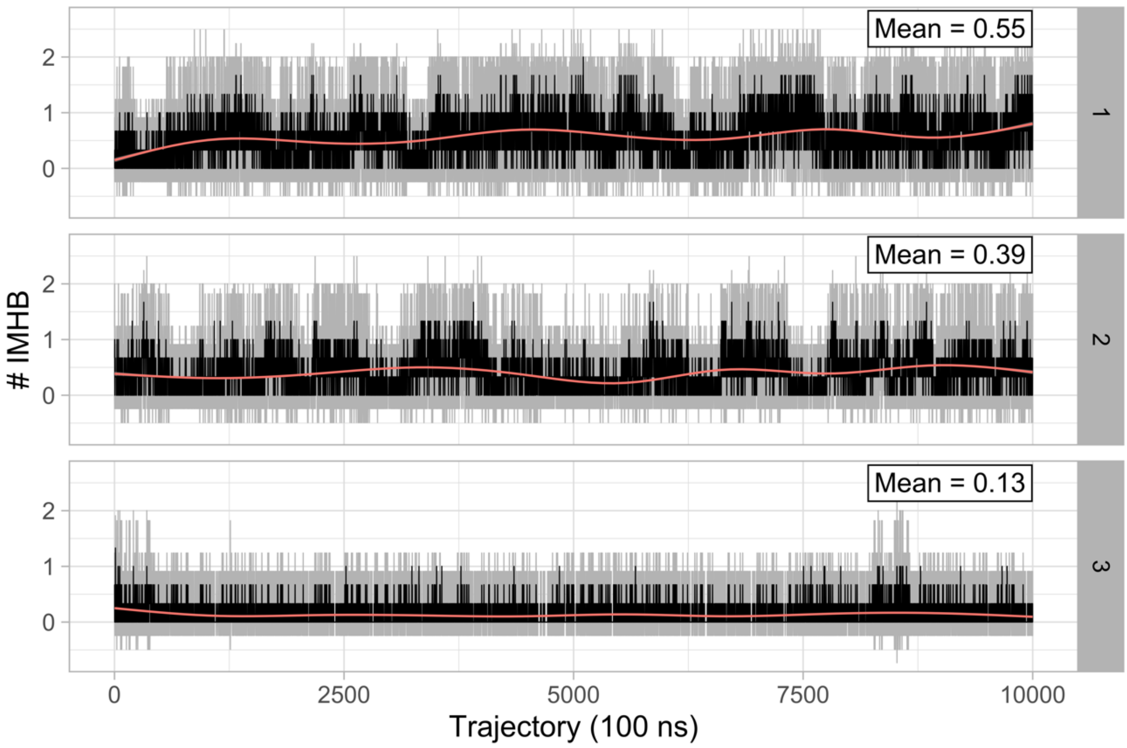

Figure S35. Intra-molecular hydrogen bond (IMHB) time series for PROTACs **1**–**3**. Averaged (black lines) over the three independent replicates, gray shading represents standard deviations.

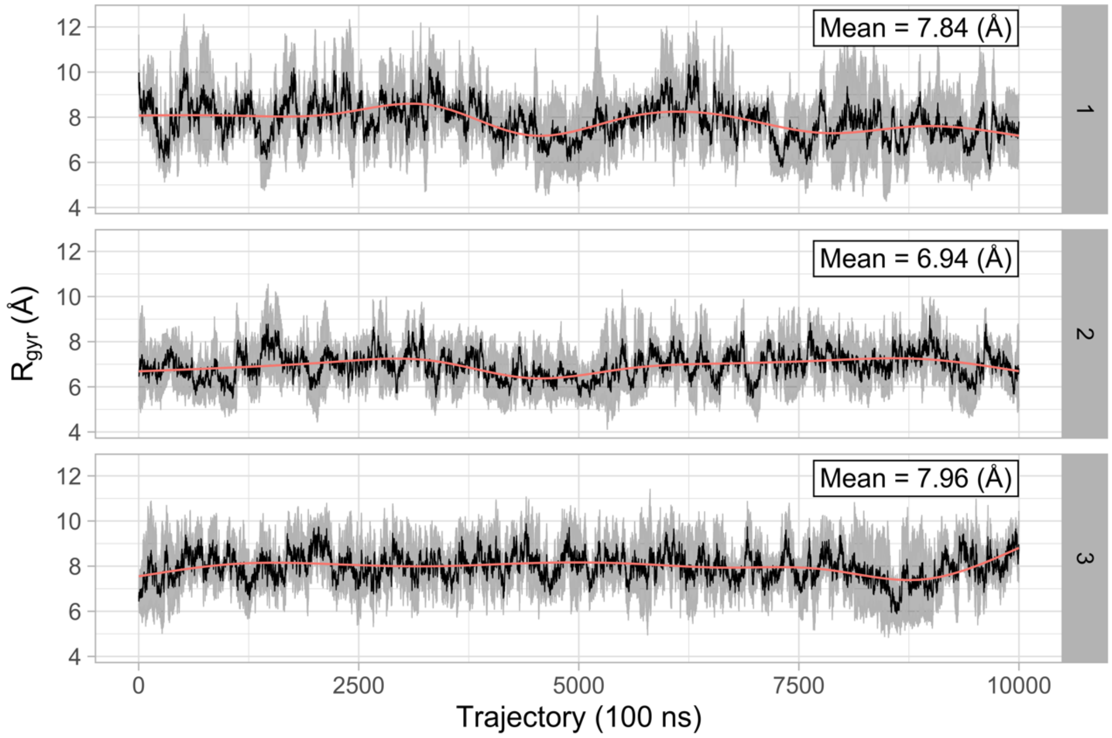

Figure S36. Radius of gyration (R_{gyr}) time series for PROTACs **1**–**3**. Averaged (black lines) over the three independent replicates, gray shading represents standard deviations.

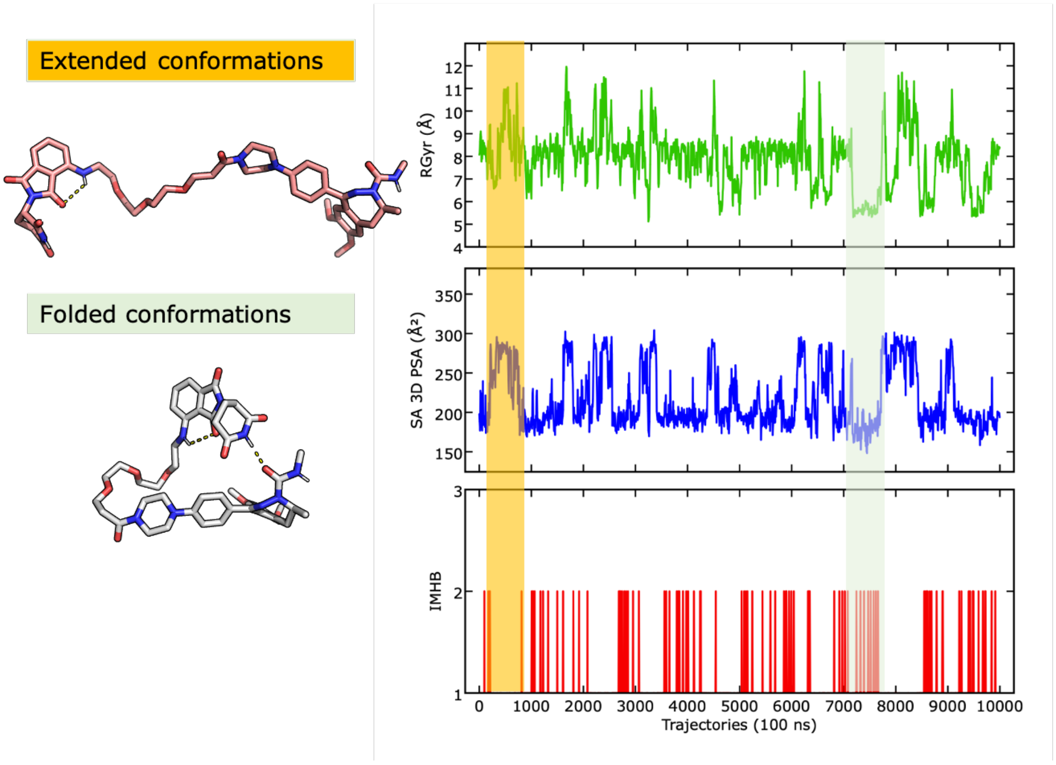

Figure S37. Comparison of molecular properties [R_{gyr} (Å), SA 3D PSA (Å^2), and IMHB] as a function of time (from MD replicate 1) for PROTAC **1.**

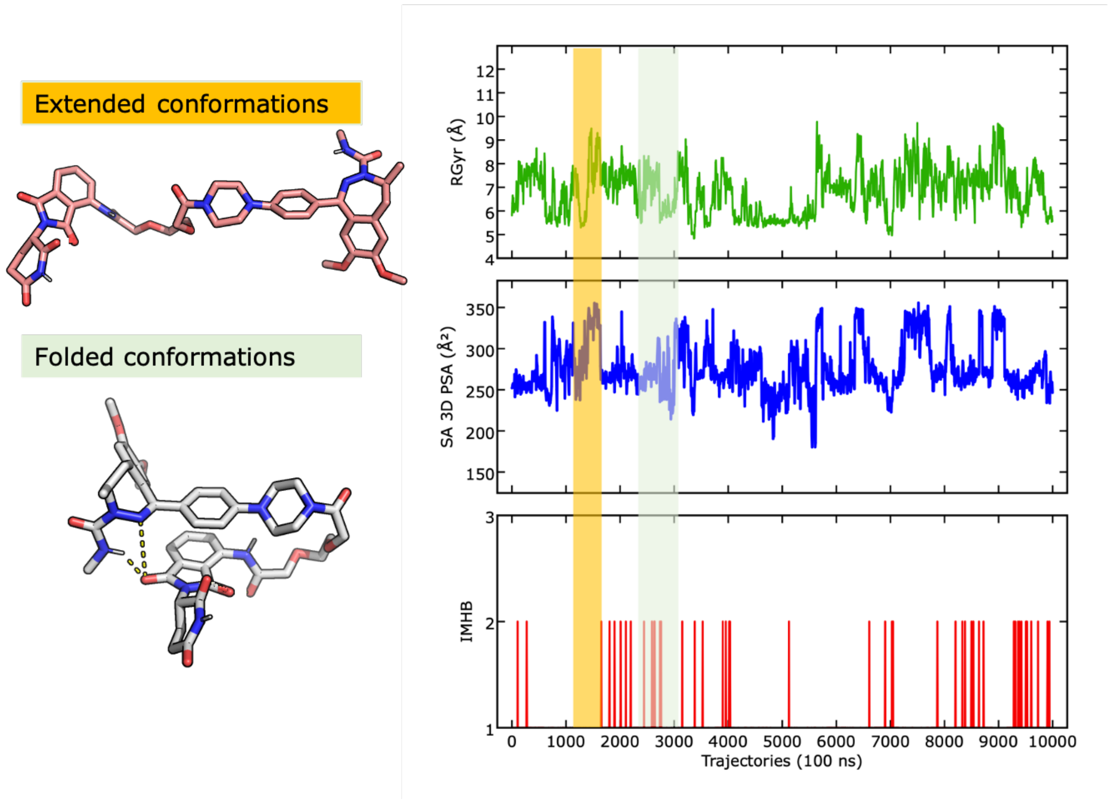

Figure S38. Comparison of molecular properties [R_{gyr} (Å), SA 3D PSA (Å^2), and IMHB] as a function of time (from MD replicate 1) for PROTAC **2.**

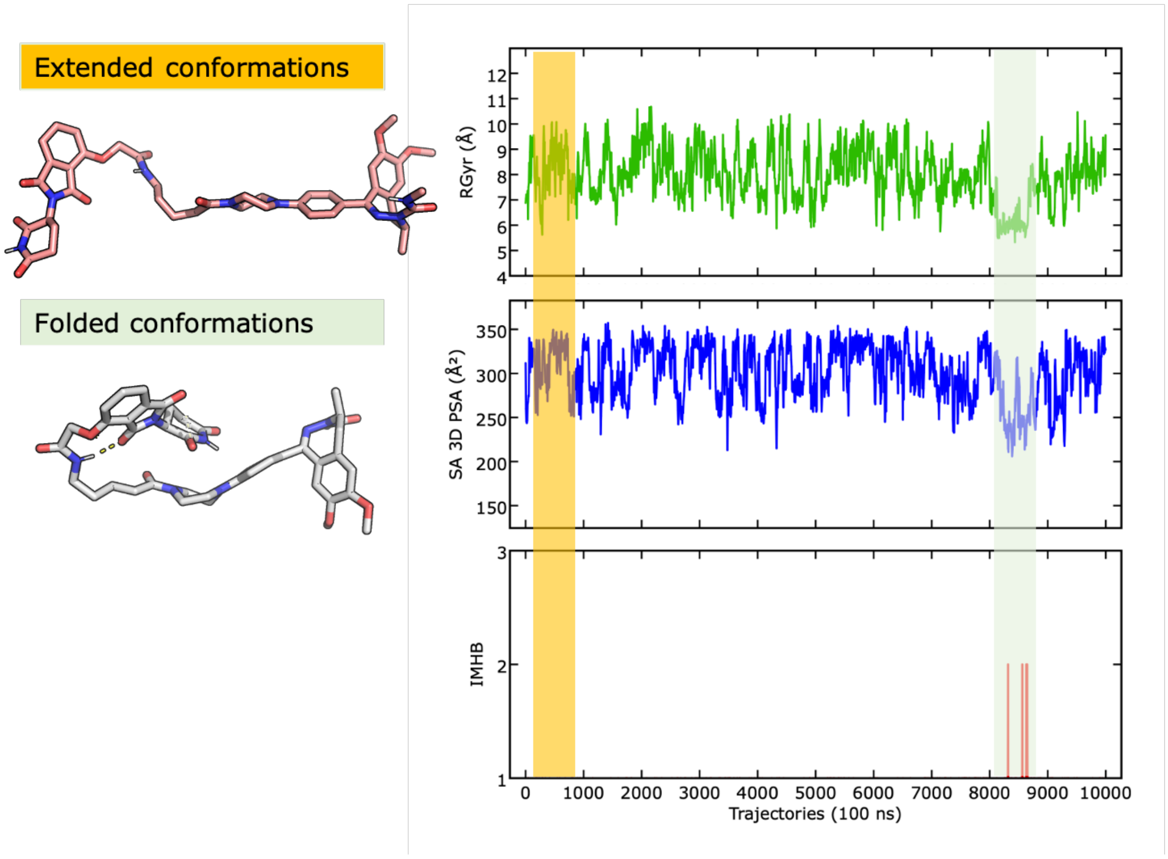

Figure S39. Comparison of molecular properties [R_{gyr} (Å), SA 3D PSA (Å^2), and IMHB] as a function of time (from MD replicate 1) for PROTAC **3.**

6.3. Principal Moments of Inertia (PMI) analysis

Table 21. PMI data for the conformations of **1** and **2** obtained by NAMFIS analysis.

PROTACs	NPR1	NPR2
**S, S-1**	0.65	0.78
	0.31	0.97
	0.36	0.81
	0.39	0.85
	0.51	0.80
**S, S-2**	0.43	0.82
	0.18	0.97
	0.19	0.94
	0.45	0.74
	0.69	0.92
	0.36	0.94
	0.55	0.79

Abbreviations: NPR1: Normalized principal moments of inertia ratio 1; NPR2: Normalized principal moments of inertia ratio 2.

Note: A summary of the PMI data for the conformations from the MD simulations of **1**–**3** is provided as a separate file.

6.4. Principal Component Analysis (PCA) of the conformations from the MD simulations

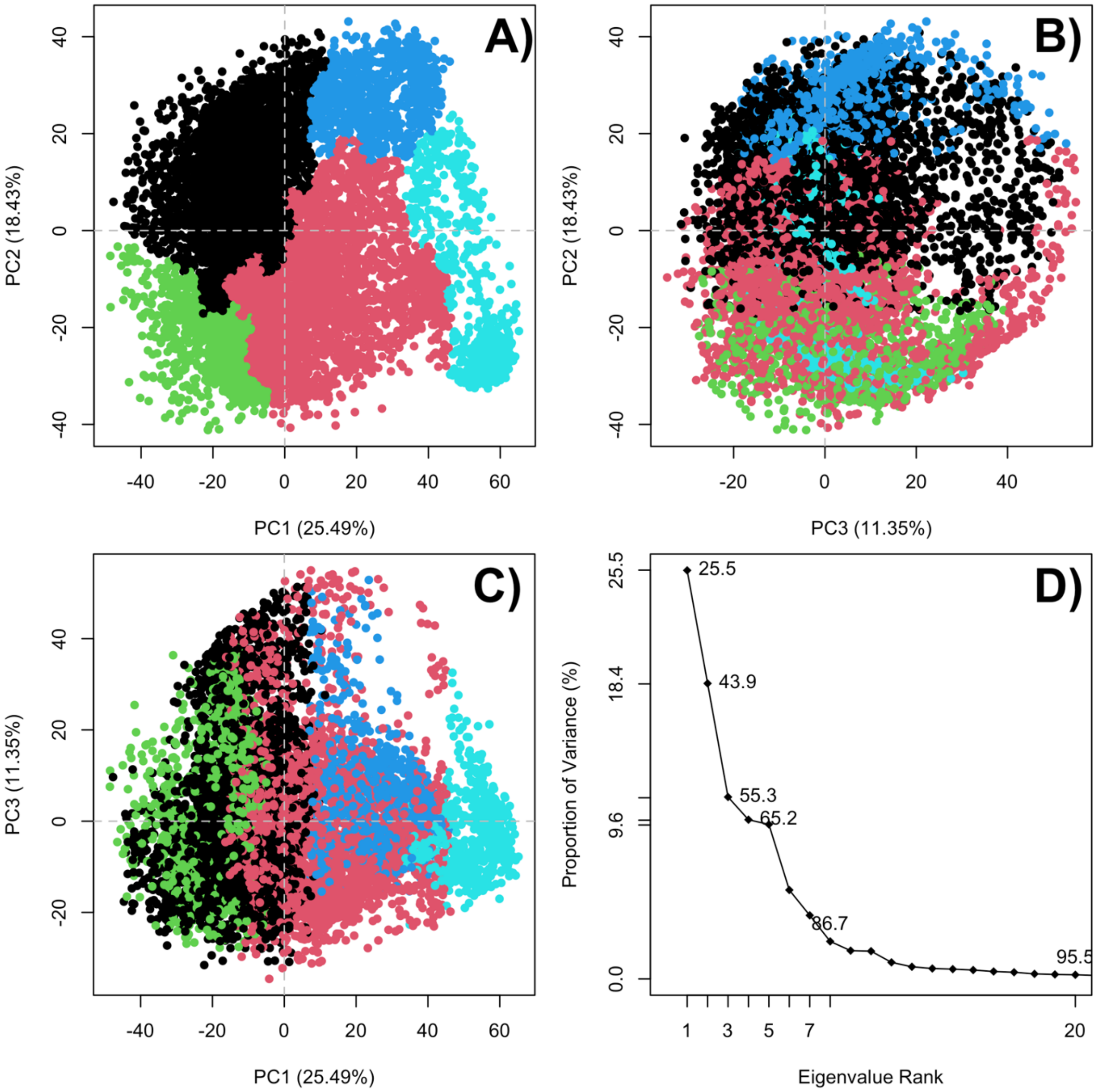

Figure S40. Overview of PCA results for PROTAC **1**. Projections of PC1 vs. PC2 (**A**); PC2 vs. PC3 (**B**); PC1 vs. PC3 (**C**). The percentage variance in each dimension is characterized by their corresponding eigenvalue as shown in panel **D**. Trajectories from the first replicate of the MD simulations.

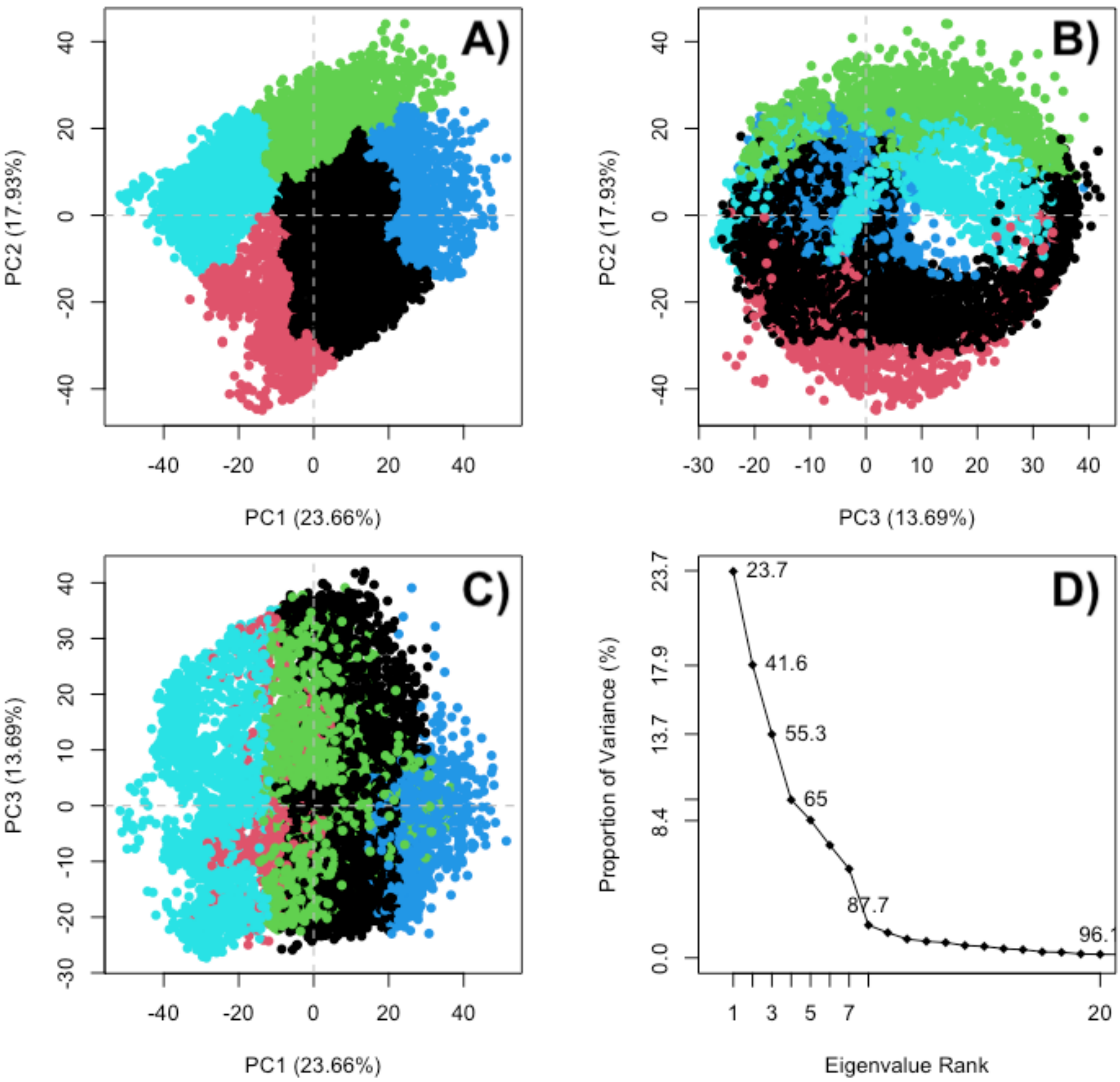

Figure S41. Overview of PCA results for PROTAC **2.** Projections of PC1 vs. PC2 (**A**); PC2 vs. PC3 (**B**); PC1 vs. PC3 (**C**). The percentage variance in each dimension is characterized by their corresponding eigenvalue as shown in panel **D**. Trajectories from the first replicate of the MD simulations.

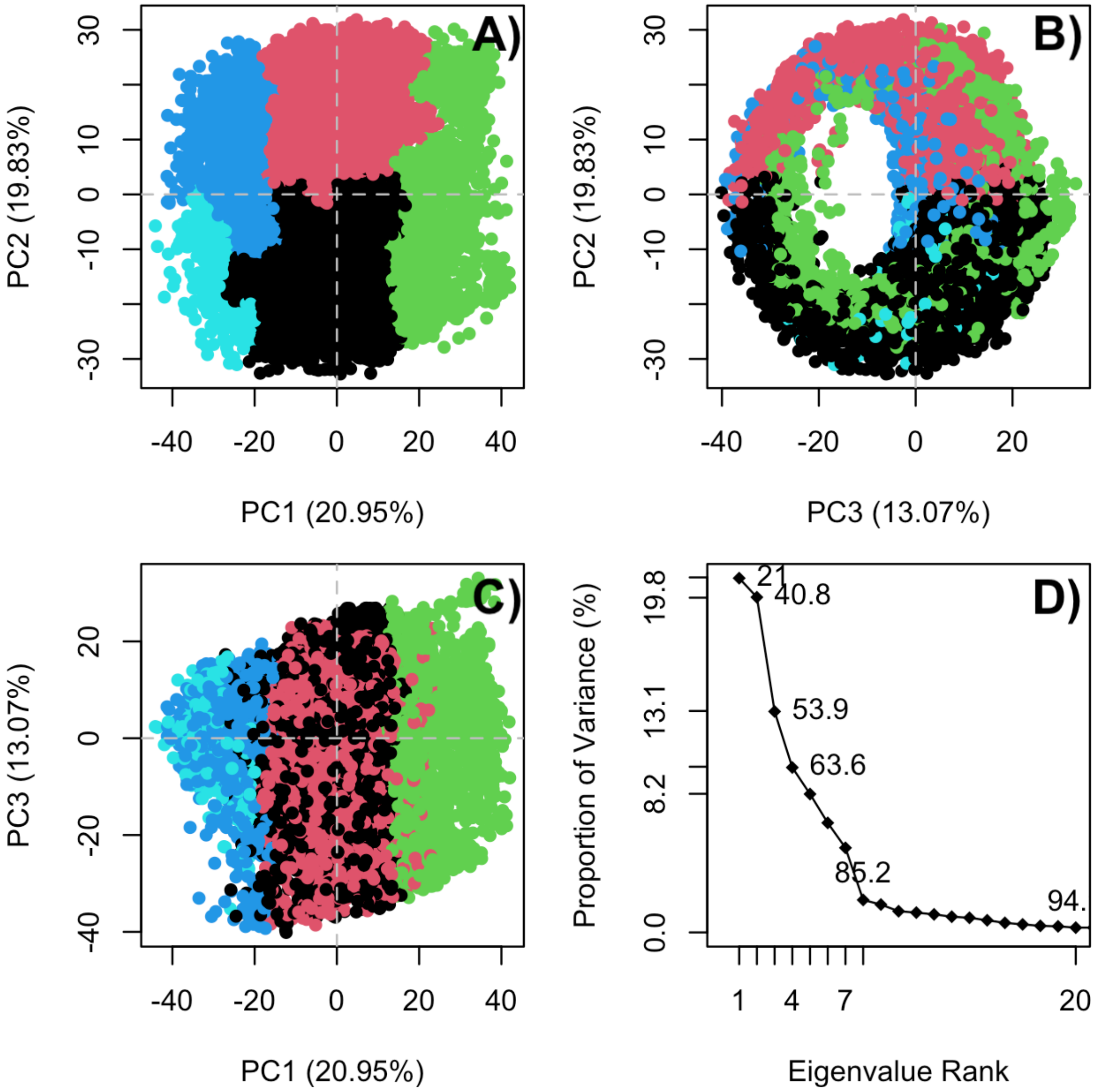

Figure S42. Overview of PCA results for PROTAC **3.** Projections of PC1 vs. PC2 (**A**); PC2 vs. PC3 (**B**); PC1 vs. PC3 (**C**). The percentage variance in each dimension is characterized by their corresponding eigenvalue as shown in panel **D**. Trajectories from the first replicate of the MD simulations.

6.5. Conformation subset selection

To further investigate the folding nature of the conformations in each of the five clusters, a subset of 26 conformations from each cluster was chosen using the *Diverse Subset* tool from the *Molecular Operating Environment* suite.[9] Principal components (PC1 and PC2) from a PCA were chosen as optional descriptors during the subset selection. Each conformation was manually analysed and classified into one of the following categories; folded, semi-folded or linear (Figure S43).

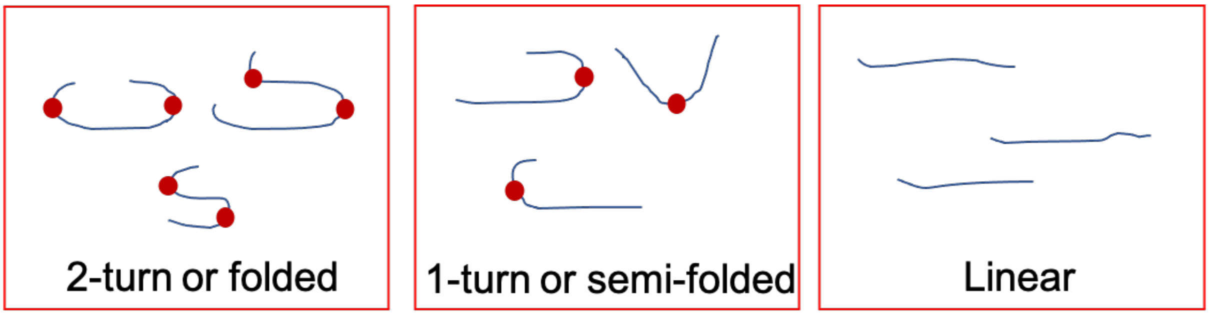

Figure S43. Schematic illustration of the three conformer classes.

Table S22. Summary of conformation classification in the five clusters of PROTACs **1–5**.

PROTAC	Class	Cluster 1	Cluster 2	Cluster 3	Cluster 4	Cluster 5
**1**	Folded	8	10	11	10	8
	Semi-folded	13	12	10	9	12
	Linear	5	4	5	7	6
**2**	Folded	7	24	2	0	25
	Semi-folded	18	2	17	12	1
	Linear	1	0	21	14	0
**3**	Folded	0	1	5	0	0
	Semi-folded	7	10	19	1	0
	Linear	19	15	2	25	26

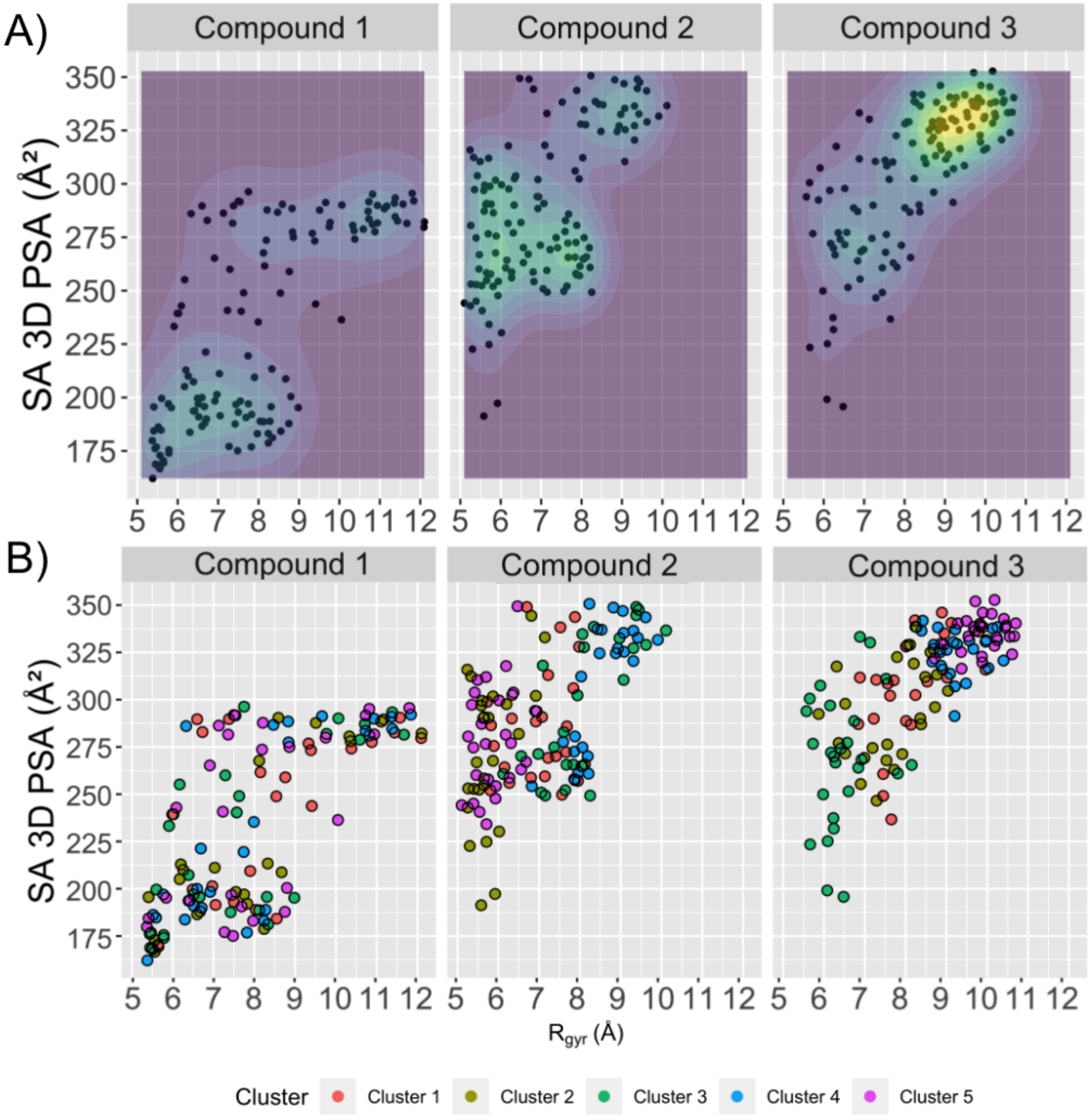

Figure S44. (**A**) Overview of the distribution of the 26 conformations in the five structural clusters of each PROTAC in chemical property space defined by the radius of gyration (R_{gyr}) and solvent accessible 3D polar surface area (SA 3D PSA). Regions of chemical space that display greater structural diversity, i.e. that are more densely populated by the selected conformations, are indicated by green-yellow color and by the contour lines. (**B**) Distribution of conformations from the five clusters in property space. Note that some conformations populate identical property space and are therefore superimposed in the panels A and B.

6.6. RMSF analysis

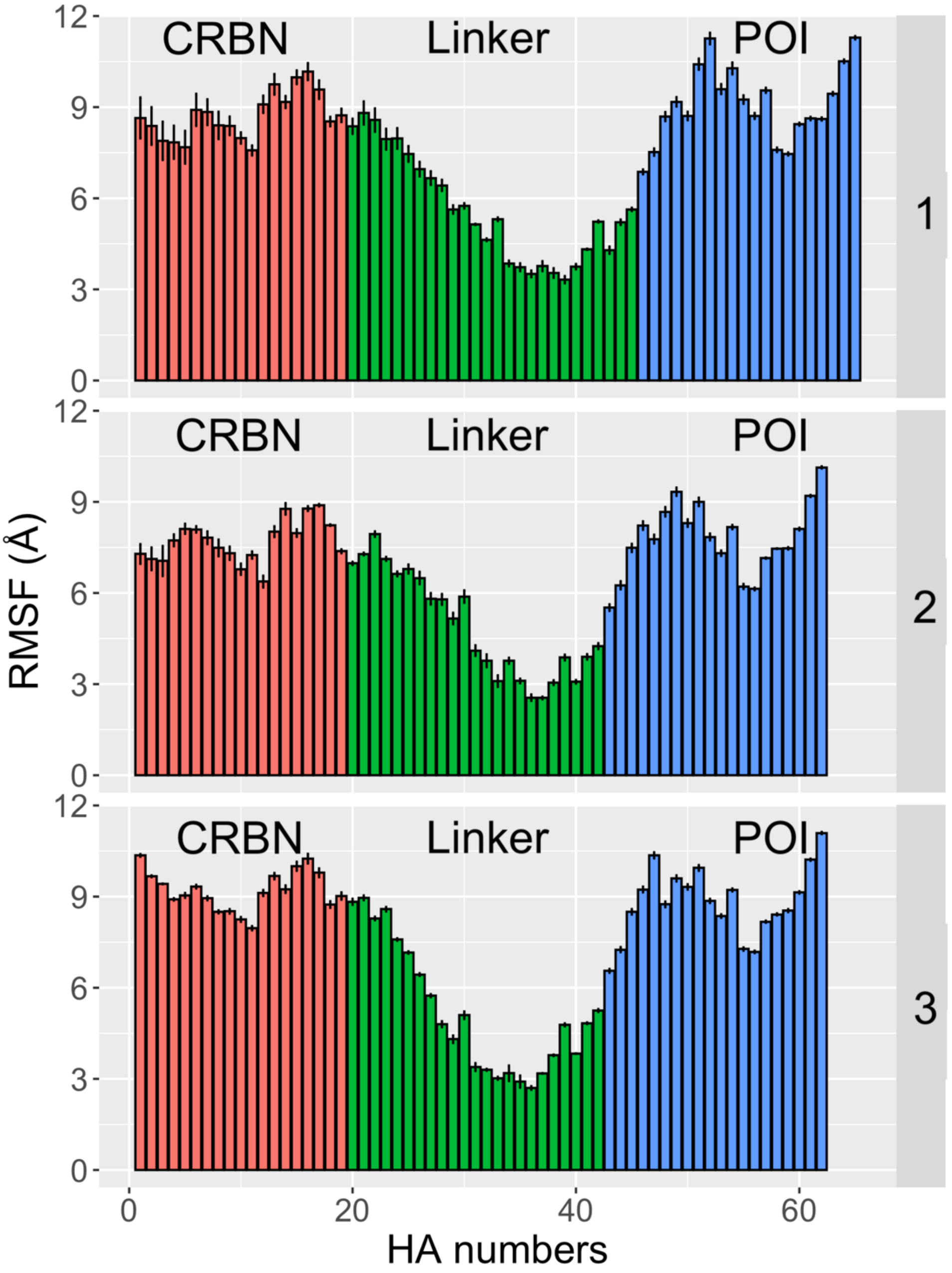

Figure S45. Plots of the root-mean-square-fluctuation (RMSF) of non-hydrogen atoms (HA) over the time from a reference position (starting coordinate) for the three parts of PROTACs **1-3.** Averaged (bar) over three independent replicates, lines represent standard deviations.

7. References

1. *Maestro Schrödinger Release 2019, Schrödinger, LLC, New York, NY, 2019.*
2. Cicero, D. O.; Barbato, G.; Bazzo, R., NMR Analysis of Molecular Flexibility in Solution: A New Method for the Study of Complex Distributions of Rapidly Exchanging Conformations. Application to a 13-Residue Peptide with an 8-Residue Loop. *Journal of the American Chemical Society* **1995,** *117* (3), 1027-1033.
3. Nevins, N.; Cicero, D.; Snyder, J. P., A Test of the Single-Conformation Hypothesis in the Analysis of NMR Data for Small Polar Molecules: A Force Field Comparison. *The Journal of Organic Chemistry* **1999,** *64* (11), 3979-3986.
4. Johnson, E. R.; Keinan, S.; Mori-Sanchez, P.; Contreras-Garcia, J.; Cohen, A. J.; Yang, W., Revealing noncovalent interactions. *J Am Chem Soc* **2010,** *132* (18), 6498-506.
5. *Jmol: an open-source Java viewer for chemical structures in 3D. http://www.jmol.org/*.
6. https://www.ch.ic.ac.uk/rzepa/cub2nci/. (accessed 10, July 2022).
7. Akaike, H., A new look at the statistical model identification. *IEEE Transactions on Automatic Control* **1974,** *19* (6), 716-723.
8. Stevens, E. S.; Sugawara, N.; Bonora, G. M.; Toniolo, C., Conformational analysis of linear peptides. 3. Temperature dependence of NH chemical shifts in chloroform. *Journal of the American Chemical Society* **1980,** *102* (23), 7048-7050.
9. *Molecular Operating Environment (MOE)*, 2019.01; Chemical Computing Group ULC: 1010 Sherbooke St. West, Suite #910, Montreal, QC, Canada, H3A 2R7, 2022., 2022.
